# Safety and efficacy of the FAKHRAVAC compared with BBIBP-Corv2 against SARS-CoV-2 in adults: a non-inferiority multi-center trial

**DOI:** 10.1186/s12985-023-02121-z

**Published:** 2023-07-18

**Authors:** Masoud Solaymani-Dodaran, Pouria Basiri, Milad Moradi, Kimiya Gohari, Ali Sheidaei, Mohammadreza Ahi, Farzad Ghafoori Naeeni, Akram Ansarifar, Zahra Rahimi, Fatemeh Gholami, Ahmad Karimi Rahjerdi, Ramin Hamidi Farahani, Kosar Naderi saffar, Soheil Ghasemi, Ali Shooshtari, Mohsen Honari, Ali Mozafari, Samane Khodaverdloo, Mohsen Forooghizadeh

**Affiliations:** 1 Clinical Trial Center (CTC) of Iran University of Medical Sciences, Tehran, Iran; 2 Stem Cell Technology Research Center (STRC), Tehran, Iran; 3 Milad Daro Noor Pharmaceutical (MDNP) Company, Tehran, Iran; 4 AJA University of Medical Sciences, Tehran, Iran; 5 Malek Ashtar University of Technology, Tehran, Iran

**Keywords:** Inactivated Covid-19 vaccine, Phase III non-inferiority trial, Fakhravac, BBIBP-Corv2

## Abstract

**Background:**

We compared Fakhravac and BBIBP-Corv2 vaccines in a phase III trial.

**Method:**

We conducted a multicenter, parallel-group, active-control, non-inferiority clinical trial with pragmatic considerations assessing the safety and efficacy of Fakhravac and BBIBP-Corv2 vaccines. We started with two randomized double-blind arms and added two non-randomized open-label arms (based on participant preference) because of slow recruitment. The adult population received 0.5 ml (10 µg per dose) intramuscular injections of Fakhravac or BBIBP-Corv-2 vaccines 21 days apart. The primary outcome was the occurrence of PCR-positive symptomatic Covid-19 disease 14 days or more after the second injection. A 10% non-inferiority margin to the reported 72.8% efficacy of BBIBP-Corv2 was assumed. Cox proportional hazard modeling was used to estimate hazard ratios and their 95% confidence intervals.

**Result:**

We enrolled 24,056 adults in four groups (randomized-Fakhravac: 824, randomized-BBIBP-Corv2: 832; Non-randomized-Fakhravac: 19,429, Non-randomized-BBIBP-Corv2: 2971). All observed local and systemic adverse reactions were generally self-limited and resolved completely. We observed similar Serious Adverse Event (SAE) rates in the BBIBP-Corv2 (2.57, 95% CI 1.33–4.49) and Fakhravac (2.25, 95% CI 1.72–2.89) groups; none of which were related to the vaccines received. We recorded 9815 Medically Attendant Adverse Events (MAAE), 736 of which were categorized as somehow related. The rate of related MAAE in the Fakhravac was similar to the BBIBP-Corv2 groups (0.31 and 0.26 per 1000 person-day) in the randomized and considerably higher (0.24 and 0.07 per 1000 person-day) in the non-randomized arms. We observed 129 (35% of the 365 required by target sample size) events of PCR + symptomatic Covid-19 during four months of active follow-up in the randomized arm, demonstrating that those receiving the Fakhravac vaccine were significantly less likely (HR = 0.69; 95% CI 0.49–0.98) to be diagnosed with PCR + symptomatic Covid-19 compared with those receiving BBIBP-Corv2 vaccine. After adjusting for type I error using the O’Brien Fleming method, the Fakhravac vaccine was non-inferior to the BBIBP-Corv2 (assuming a 10% non-inferiority margin to the reported 72.8% BBIBP-Corv2 vaccine efficacy; HR < 1.35) (One-way test: HR = 0.66; 99.8% CI 0.38–1.15). In the non-randomized arm, the results were inconclusive (HR = 1.23; 95% CI 0.96–1.61). We observed 5 cases of hospitalized Covid-19 in the randomized arm, none of which occurred in the Fakhravac vaccine group. Those receiving the Fakhravac vaccine were four times less likely to go to the hospital because of a Covid-19 diagnosis (HR = 0.24; 95% CI 0.10–0.60). The vaccine efficacy of the Fakhravac vaccine is estimated to be 81.5% (95% CI 81–82.4%).

**Conclusion:**

Fakhravac inactivated SARS-CoV-2 vaccine has comparable safety and efficacy to the BBIBP-Corv2 vaccine.

*Trial registration* This study was registered with the Iranian Registry of Clinical Trials (www.irct.ir: IRCT20210206050259N3).

**Supplementary Information:**

The online version contains supplementary material available at 10.1186/s12985-023-02121-z.

## Background

Fakhravac is an inactivated SARS-CoV-2 vaccine based on the SARS-CoV-2 IR-sb2-01 strain cultured in a Vero cell line. The IR-sb2-01 strain was isolated from hospitalized patients who had severe clinical illnesses. The virus was inactivated by formaldehyde and emulsified with aluminum hydroxide adjuvant to formulate the final vaccine product [1].

The inactivated vaccines are the oldest and have been used for almost a century. They are safe and can be easily stored and shipped at 2–8 °C, making them suitable for many low-income countries and places with limited cold-storage capacity [2]. The early ones that entered the WHO emergency use listing and became available outside their manufacturing countries were BBIBP-Corv2 and Sinovac [3]. The inactivated BBIBP-Corv2 was used for mass vaccination of the general Iranian population against Covid-19 in the second half of 2021.

The phase I and II trial of Fakhravac compared to the placebo was conducted in early 2021. The studies showed that it is generally safe and induces strong specific antibody responses against SARS-CoV-2 spike antigens in adults 18 years and older. Given the availability of a licensed vaccine, the use of a placebo was no longer justified [4]. We have therefore conducted a multi-center, phase III, non-inferiority trial to assess the efficacy and safety of Fakhravac against BBIBP-Corv2 in adults.

## Method

### Study design

We conducted a multicenter, parallel-group, active-control, non-inferiority clinical trial with pragmatic considerations comparing the safety and efficacy of Fakhravac and BBIBP-Corv2 vaccines. It started with two randomized and double-blind arms. We added two additional non-randomized and open-label arms of Fakhravac and BBIBP-Corv2 because of slow recruitment (Comprehensive cohort study design). Participants who declared unwillingness to randomization were retained in the study, but their vaccine choice was based on their preference [5]. Written informed consent was obtained from all participants before enrollment, with slight differences based on their manner of allocation (randomized or not randomized).

This study was conducted in collaboration with the Iran University of Medical Science (IUMS) clinical trial center as the academic contract research organization (CRO). The National Research Ethics Committee approved the study protocol (IR. NREC.1400.006, 24th August 2021).

### Participants

Volunteer enrollment was managed via the study web-based software. They could read the informed consent and go through an initial self-administered screening questionnaire. Those who passed this stage were invited to attend the clinical trial centers for face-to-face screening by the study officers and signing the written informed consent form. People aged 18 or older were included. Major exclusion criteria were as follows: pregnancy and lactation; History of receiving Covid-19 vaccine; Close contact with a definite case of Covid-19 up to two weeks before the day of receiving the first dose; Acute febrile illness; Current acute or chronic symptomatic illness that requires ongoing medical or surgical care; History of allergic diseases such as angioedema or anaphylactic reactions following the use of drugs, vaccines or food; History of long-term use (14 successive days) of immunosuppressive drugs or systemic corticosteroids in the last four months leading up to the study; History of diagnosis or treatment for HIV; Current drug or alcohol abuse (addiction); Chronic diseases that are not listed as exclusion criteria but are considered unstable within the last four weeks. Detailed inclusion and exclusion criteria are provided in the protocol (for more details please see the protocol).

### Randomization and masking

The study epidemiologist created the random allocation sequence to assign the participants to the BBIBP-CorV or Fakhravac vaccine groups (in a 1:1 ratio) in the randomized arms using a variable-sized (4 and 6) block randomization method. Stata software was used for this purpose. We concealed the random allocation sequence using nine-character unique codes and embedded them into the study software. The software allocated the codes to the participants once their eligibility was confirmed. The person responsible for blinding delivered the assigned vaccine for administration by the vaccinator, who was unaware of the type of vaccine used. Participants and the rest of the research team remained masked to the type of vaccine. All vaccine doses had the same volume and color and were prepared in identical syringes.

### Procedures

We monitored immediate reactions and vital signs following vaccine injections for half an hour at the clinical trial centers. All collected information was recorded in the web-based study software.

We asked the participants to report their daily local (pain, tenderness, swelling, and redness) and systemic (nausea/vomiting, diarrhea, headache, fatigue, and myalgia) adverse reactions in a mobile application during the week after each injection. In the same session, the mobile application was installed and activated on the participants' mobile phones and was an integral part of the web-based study software. Participants were also asked to report weekly any visits to local surgery or hospital A&E (Accidents and Emergency) on their mobile application during the four months of active follow-up. Missing two successive weekly reports triggered a notification for the follow-up team to contact the participant. They could also phone at any time (24/7) study follow-up center where they could consult a resident physician or report their illness or any other matter of concern. Any report of illness or seeking medical advice in the mobile application or direct contact with the follow-up center opened an electronic adverse event file that was followed up until complete resolution.

### Outcomes

The primary outcome was the occurrence of PCR-positive symptomatic Covid-19 disease 14 days or more after the second injection. A SARS-CoV-2 RT-PCR test was requested for all participants with clinical symptoms suspicious of SARS-CoV-2 infection, including fever (temperature ≥ 38 °C), chills, cough, shortness of breath, myalgia, headache, sore throat, diarrhea, anosmia, or hyposmia. All suspected or confirmed cases were followed up daily until all symptoms were resolved.

The secondary outcomes were severe cases of Covid-19 disease that had been hospitalized or died 14 days or more after the second injection, immediate reactions including anaphylaxis following vaccine injections, local and systemic adverse reactions within the first-week post-vaccination, and any Medically Attended Adverse Events (MAAEs) including Serious Adverse Events/Reactions (SAEs) and Suspected Unexpected Serious Adverse Reactions (SUSARs) occurring within the four months active follow-up. Severe Covid-19 cases were defined by one or more of the following criteria: respiratory rate above 30 per minute; heart rate at or exceeding 125 beats per minute; oxygen saturation at 93% or less while the participant was breathing ambient air at sea level or a ratio of the partial pressure of oxygen to the fraction of inspired oxygen below 300 mm Hg; respiratory failure; acute respiratory distress syndrome; evidence of shock (systolic blood pressure < 90 mm Hg, diastolic blood pressure < 60 mm Hg, or a need for vasopressors); clinically significant acute renal, hepatic, or neurologic dysfunction; admission to an intensive care unit; or death.

### Statistical analysis

We needed 365 events of the primary outcome to demonstrate the non-inferiority of Fakhravac based on a 10% non-inferiority margin of the reported 72.8% efficacy of BBIBP-Corv-2 [6]. The calculated hazard ratio for the lower margin of the non-inferiority was 1.35. by using conservative forecast figures of Covid-19 incidence over six months, a target sample size of 41,128 was estimated to reach the needed 365 events of PCR-positive symptomatic Covid-19 disease (for more details, see the protocol). We used the O’Brien Fleming method to correct the *p* values and estimated confidence intervals in the interim analyses not to exceed the overall amount of 0.05 conventional type I error in a 2-sided statistical test [7, 8] (equivalent to α = 0.025 in one-sided statistical test). Analysis of both randomized and non-randomized arms was conducted separately and in parallel. In the interim analyses, the results were also pooled using the stratified Cox proportional hazard model.

We used frequencies (percentages) for categorical variables and mean (SD) for continuous variables to compare baseline values in the study groups and report adverse events/reactions. All those who received the standard first dose made the safety and the intention-to-treat (ITT) efficacy populations. The ITT population was used for sensitivity analysis. The modified ITT efficacy population included participants who received both vaccine doses.

We used Kaplan Meier survival analysis and log-rank test to compare the occurrence of the outcomes in the study groups. Cox proportional hazard modeling was used to estimate hazard ratios and their 95% confidence intervals. Model assumptions were checked using proportional hazard graphs, Schoenfeld residuals against time plots, and formal proportional hazard tests. We used the Stata 11 and R 4.3.2 software.

An eight-member data and safety monitoring board (DSMB) oversaw the conduct of the study and received periodic safety reports. The DSMB consisted of one person from each of the following specialties: clinical pharmacotherapist, epidemiologist, cardiologist, infectious disease specialist, oncologist, and representatives from the regularity authorities (FDO), the National Ethics Committee, and the Ministry of Health’s Center for Disease Control.

This study was registered with the Iranian Registry of Clinical Trials (www.irct.ir: IRCT20210206050259N3).

### Role of the funding source

The funder of the study had no role in study design, data collection, data analysis, data interpretation, or writing of the report.

## Results

**Fig. 1:**
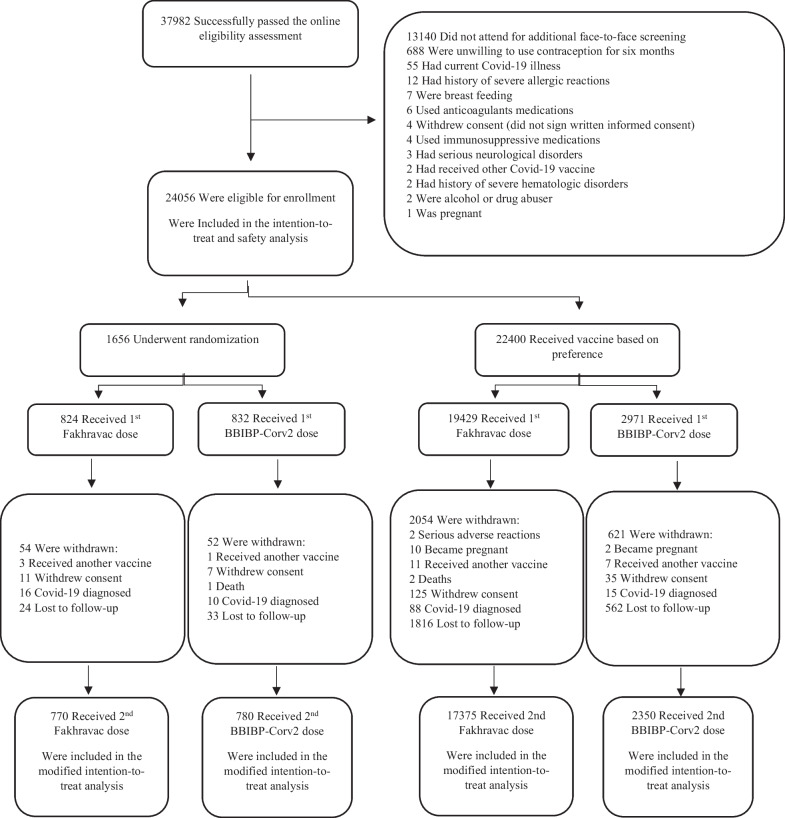
Flow of participants in a study of non-inferiority evaluation of efficacy, safety, and immunogenicity of Fakhravac versus BBIBP-Corv2

Between September 1 and December 31, 2021, 24,056 adults were enrolled in four clinical trial centers, which made our intention-to-treat population. We randomly allocated 1656 participants to receive Fakhravac (824) or BBIBP-Corv2 (832) vaccines. The remaining 22,400 participants who declared their unwillingness for random allocation received either Fakhravac (19,429) or BBIBP-Corv2 (2971) vaccines based on their preference. We delivered the second dose to 21,303 participants (1553 in the randomized arm and 19,750 in the non-randomized arm), constituting our modified intention-to-treat population (Fig. 1).

Baseline characteristics in the randomized arm were the same in the BBIBP-Corv2 and Fakhravac groups. In the non-randomized arm, there was a tendency to receive the Fakhravac vaccine in women with higher educational achievements. Age, BMI, and comorbidities were the same among the participants of both vaccine groups regardless of being in the randomized or non-randomized arms (Table 1).

**Table 1:** Baseline characteristics in the safety and intention-to-treat population

Characteristic	Fakhravac random N = 824	BBIBP-Corv2 random N = 832	Fakhravac non-random N = 19,429	BBIBP-Corv2 non-random N = 2971	Fakhravac overall N = 20,253	BBIBP-Corv2 overall N = 3803	Overall N = 24,056
Sex n (%)							
Female	306 (37%)	310 (37%)	9216 (47%)	1267 (43%)	9522 (47%)	1577 (41%)	11,099 (46%)
Male	518 (63%)	522 (63%)	10,210 (53%)	1702 (57%)	10,728 (53%)	2224 (59%)	12,952 (54%)
Age mean (SD)	30.9 (8.7)	31.3 (9)	34 (11.1)	34.1 (11.3)	35.9 (10.9)	35.5 (10.8)	33.8 (11)
18–30 n (%)	297 (36%)	309 (37%)	5763 (30%)	932 (31%)	6060 (30%)	1241 (33%)	7301 (30%)
30–40 n (%)	351 (43%)	334 (40%)	6982 (36%)	982 (33%)	7333 (36%)	1316 (35%)	8649 (36%)
40–50 n (%)	152 (18%)	153 (18%)	4447 (23%)	703 (24%)	4599 (23%)	856 (23%)	5455 (23%)
50–60 n (%)	23 (2.8%)	31 (3.7%)	1647 (8.5%)	244 (8.2%)	1670 (8.2%)	275 (7.2%)	1945 (8.1%)
60+ n (%)	1 (0.1%)	5 (0.6%)	584 (3.0%)	108 (3.6%)	585 (2.9%)	113 (3.0%)	698 (2.9%)
BMI mean (SD)	26.1 (5)	25.8 (4.6)	25.7 (4.5)	25.1 (3.8)	25.7 (4.5)	25.3 (4)	25.7 (4.5)
Education n (%)							
Elementary	17 (2.1%)	18 (2.2%)	543 (2.8%)	147 (5.0%)	560 (2.8%)	165 (4.3%)	725 (3.0%)
Below diploma	78 (9.5%)	70 (8.4%)	2487 (13%)	531 (18%)	2565 (13%)	601 (16%)	3166 (13%)
Diploma	282 (34%)	290 (35%)	6708 (35%)	1070 (36%)	6990 (35%)	1360 (36%)	8350 (35%)
Above diploma	74 (9.0%)	87 (10%)	1746 (9.0%)	224 (7.5%)	1820 (9.0%)	311 (8.2%)	2131 (8.9%)
Bachelor	262 (32%)	267 (32%)	6003 (31%)	774 (26%)	6265 (31%)	1041 (27%)	7306 (30%)
Master	91 (11%)	84 (10%)	1667 (8.6%)	172 (5.8%)	1758 (8.7%)	256 (6.7%)	2014 (8.4%)
Doctoral and above	19 (2.3%)	16 (1.9%)	272 (1.4%)	51 (1.7%)	291 (1.4%)	67 (1.8%)	358 (1.5%)
Comorbidities n (%)							
High blood pressure	14 (1.7%)	21 (2.5%)	541 (2.8%)	72 (2.4%)	555 (2.7%)	93 (2.4%)	648 (2.7%)
Chronic heart disease	5 (0.6%)	10 (1.2%)	135 (0.7%)	20 (0.7%)	140 (0.7%)	30 (0.8%)	170 (0.7%)
Chronic non-asthma lung dis	1 (0.1%)	2 (0.2%)	102 (0.5%)	13 (0.4%)	103 (0.5%)	15 (0.4%)	118 (0.5%)
Asthma	6 (0.7%)	6 (0.7%)	118 (0.6%)	14 (0.5%)	124 (0.6%)	20 (0.5%)	144 (0.6%)
Chronic kidney disease	1 (0.1%)	2 (0.2%)	59 (0.3%)	14 (0.5%)	60 (0.3%)	16 (0.4%)	76 (0.3%)
Moderate/severe liver dis	1 (0.1%)	0 (0%)	42 (0.2%)	5 (0.2%)	43 (0.2%)	5 (0.1%)	48 (0.2%)
(Fatty liver) Mild liver disease	27 (3.3%)	36 (4.3%)	453 (2.3%)	30 (1.0%)	480 (2.4%)	66 (1.7%)	546 (2.3%)
Chronic neurological disease	6 (0.7%)	9 (1.1%)	146 (0.8%)	26 (0.9%)	152 (0.8%)	35 (0.9%)	187 (0.8%)
Diabetes, no complications	7 (0.8%)	10 (1.2%)	340 (1.8%)	46 (1.5%)	347 (1.7%)	56 (1.5%)	403 (1.7%)
Diabetes with complications	0 (0%)	1 (0.1%)	54 (0.3%)	10 (0.3%)	54 (0.3%)	11 (0.3%)	65 (0.3%)
Chronic blood disease	6 (0.7%)	5 (0.6%)	127 (0.7%)	11 (0.4%)	133 (0.7%)	16 (0.4%)	149 (0.6%)
Rheumatic diseases	1 (0.1%)	3 (0.4%)	81 (0.4%)	12 (0.4%)	82 (0.4%)	15 (0.4%)	97 (0.4%)
Dementia	0 (0%)	0 (0%)	26 (0.1%)	2 (< 0.1%)	26 (0.1%)	2 (< 0.1%)	28 (0.1%)
History of covid-19 n (%)							
No	804 (97.6%)	809 (97.2%)	17,162 (88.4%)	2814 (94.8%)	17,966 (88.8%)	3623 (95.3%)	21,589 (89.8%)
Yes	20 (2.4%)	23 (2.8%)	2253 (11.6%)	154 (5.2%)	2273 (11.2%)	177 (4.7%)	2450 (10.2%)

**Fig. 2:**
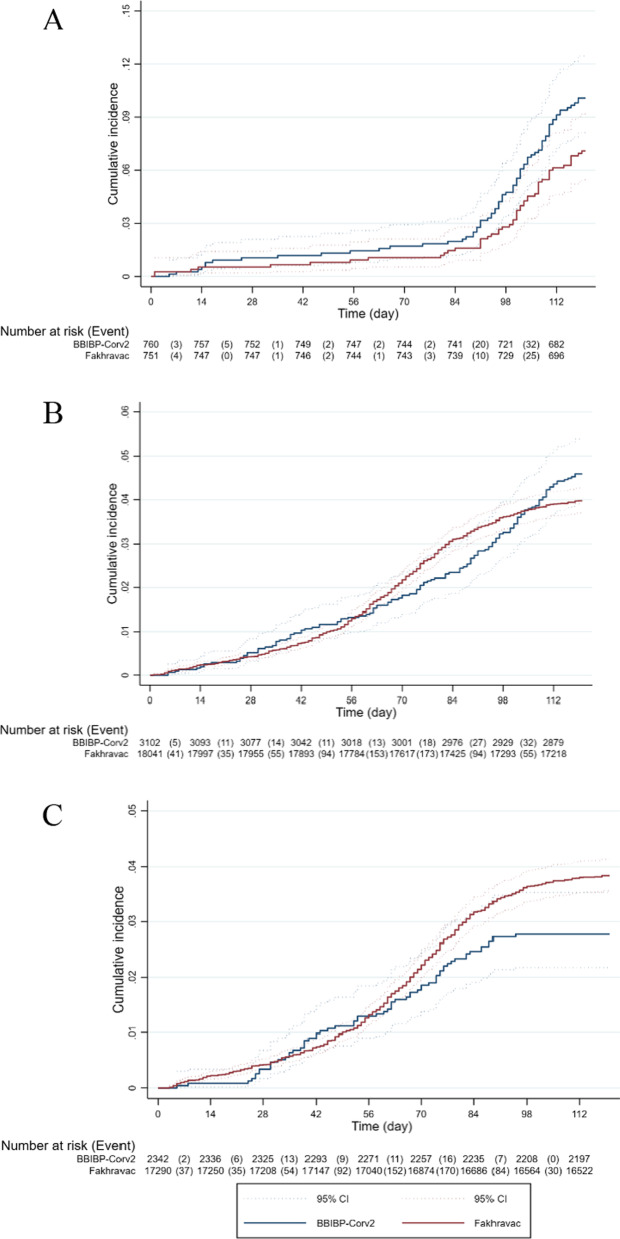
Kaplan–Meier curve of PCR-positive Covid-19 disease events 14 days after the second injection in the randomized arm (**a**), in the non-randomized arm (**b**), and in total (**c**)

We identified 855 PCR + symptomatic Covid-19 cases diagnosed two weeks after the second injection in total of 2,480,583 person-days of follow-up. In the randomized arm, the number of events reached 129, which was 35% of the required 365 based on the sample size calculations. The incidence of PCR + symptomatic Covid-19 was lower in the Fakhravac (Inc. = 0.60 per 1000; 95% CI 0.46–0.78) compared with the BBIBP-Corv2 (Inc. = 0.86 per 1000; 95% CI 0.69–1.08) vaccine groups. In a Cox proportional hazard model, those receiving the Fakhravac vaccine were significantly less likely (HR = 0.69; 97.5% CI 0.49–0.98) to be diagnosed with PCR + symptomatic Covid-19 compared with those receiving BBIBP-Corv2 vaccine. Adjusting the type I error for 35% information fraction using the O’Brien Fleming method and calculating a 99.8% confidence interval (one-way CI = 99.9%) increased the upper limit of the confidence interval of the hazard ratio to 1.20 which is within our calculated 1.35 non-inferiority margin (see protocol for details). This likelihood remained relatively unchanged after adjusting for age, sex, education, history of Covid-19 diagnosis, and calendar week of receiving the first injection (One-way test: HR = 0.67; 97.5% CI 0.47–0.96; 99.9% CI 0.39–1.17). Based on results from the randomized arm, we estimated vaccine efficacy for Fakhravac at 81.5% (95% CI 0.81, 0.82) using a network meta-analysis approach. In comparison with the randomized arm, the incidences of PCR + symptomatic Covid-19 diagnosed two weeks after the second injection in the non-randomized arm were considerably lower in both vaccine groups, particularly in the BBIBP-Corv2 vaccine recipients. In the Cox proportional hazard model, after adjusting for age, sex, education, history of Covid-19 diagnosis, and calendar week of receiving the first injection, the results were inconclusive (HR = 1.23; 97.5% CI 0.96–1.61). There were no serious violations of the proportional hazard assumption in all models used (Table 2 and Fig. 2).

**Table 2:** Hazard ratios and 95% CI resulted from Cox proportional hazard regression modeling for PCR + symptomatic Covid-19 cases diagnosed two weeks after the second injection in the study groups

Study arms	Random arm			Non-random arm		Total^a^	
Vaccine groups	BBIBP-Corv2	Fakhravac (CI: 97.5%)	Fakhravac^b^ (CI: 99.9%)	BBIBP-Corv2	Fakhravac	BBIBP-Corv2	Fakhravac
Person time, days	88,453	88,415		272,252	2,031,463	360,705	2,119,878
Number of events	76	53		64	662	140	715
Incidence (95% CI)^c^	0.86 (0.69,1.08)	0.60 (0.46,0.78)		0.24 (0.18,0.30)	0.33 (0.30,0.35)	0.39 (0.33,0.46)	0.34 (0.31,0.36)
Unadjusted HR	Ref	0.69 (0.49,0.98)	0.69 (0.40,1.20)	Ref	1.38 (1.07,1.78)	Ref	1.3 (0.98, 1.72)
Adjusted HR^d^	Ref	0.67 (0.47,0.96)	0.67 (0.39,1.17)	Ref	1.32 (1.01,1.69)	Ref	0.84 (0.7, 1.01)
Adjusted HR^e^	Ref	0.68 (0.48,0.96)	0.68 (0.39,1.18)	Ref	1.25 (0.97,1.61)	Ref	0.9 (0.75, 1.09)
Adjusted HR^f^	Ref	0.67 (0.47,0.96)	0.67 (0.39,1.17)	Ref	1.23 (0.96,1.61)	Ref	1.02 (0.74, 1.43)
Adjusted HR^g^	Ref	0.67 (0.47,0.96)	0.67 (0.39,1.17)	Ref	1.30 (0.99,1.70)	Ref	0.9 (0.75, 1.06)

^a^Results from stratified cox proportional hazard regression, combining random and non-random arms

^b^Confidence interval after adjusting for α error using O’Brien Fleming method in one sided statistical test

^c^Incidence per 1000

^d^Adjusted for age, sex, and education

^e^Adjusted for age, sex, education, and calendar week of the 1st injection

^f^Adjusted for age, sex, education, calendar week of the 1st injection, and history of covid-19

^g^Adjusted for age, sex, education, calendar week of the 1st injection, restricting the analysis to participants without a history of covid-19

**Fig. 3:**
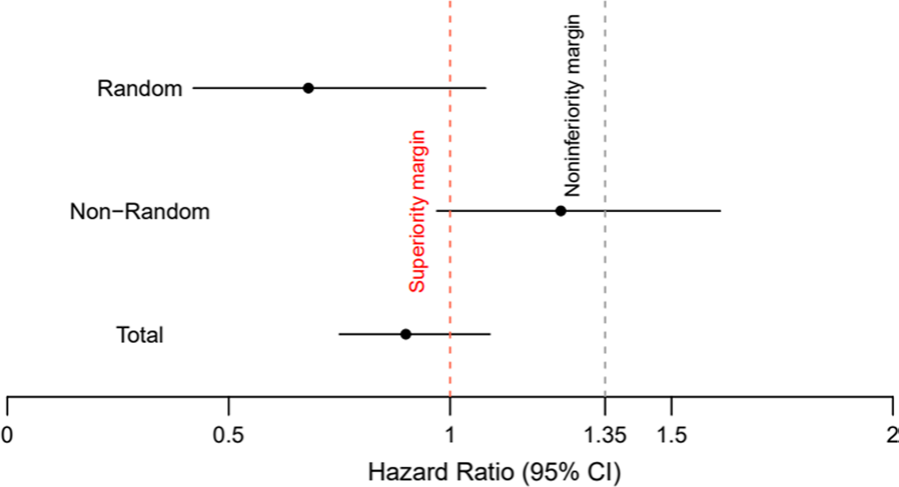
The hazard ratio of the Fakhravac compared to the BBIBP-Corv2 in PCR-positive Covid-19 cases (**a**) and hospitalized cases (**b**)

We observed 5 cases of hospitalized Covid-19 in the randomized arm, none of which occurred in the Fakhravac vaccine group. This number was 14 in the non-randomized arm (Table 3). Overall, those receiving the Fakhravac vaccine were four times less likely to go to a hospital because of Covid-19 (HR = 0.24; 95% CI 0.10–0.60) (Fig. 3).

**Table 3:** Hazard ratios and 95% CI resulted from Cox proportional hazard regression modeling for hospitalized Covid-19 cases diagnosed two weeks after the second injection in study groups

Study arms	Total^a^	
Vaccine groups	BBIBP-Corv2	Fakhravac
Person time, days	360,705	2,119,878
Number of events	8	11
Incidence^e^ (95% CI)	0.22 (0.11,0.44)	0.05 (0.03,0.09)
Unadjusted HR (95% CI)	Ref	0.23 (0.09, 0.58)
Adjusted HR^b^ (95% CI)	Ref	0.23 (0.09, 0.57)
Adjusted HR^c^ (95% CI)	Ref	**0.24 (0.1, 0.6)**
Adjusted HR^d^ (95% CI)	Ref	0.34 (0.13, 0.87)

^a^Results from stratified cox proportional hazard regression, combining random and non-random arms

^b^Adjusted for age, sex, and education

^c^Adjusted for age, sex, education, and calendar week of the 1st injection

^d^Adjusted for age, sex, education, calendar week of the 1st injection, and history of covid-19

^e^Incidence per 1000

All observed local reactions were self-limited and resolved completely. No grade IV local reactions were seen. The most common local adverse reaction was mild tenderness (grade I and II) after injection, which occurred in 40% and 13% of the participants after the first injection in the Fakhravac and BBIBP-Corv2 groups. These figures were 29% and 8% after the second injection. Grade I and II (mild to moderate) local pain was reported by 24% and 10% of the participants after the first injection in the Fakhravac and BBIBP-Corv2 groups. The figures following the second injection were 18% and 7%. On 71 occasions, the injection site pain resulted in taking narcotic painkillers (grade III); all but three occurred in the non-randomized arm receiving the Fakhravac vaccine and resolved spontaneously. The occurrence of other local reactions, such as induration and redness, was rare in both groups.

**Fig. 4:**
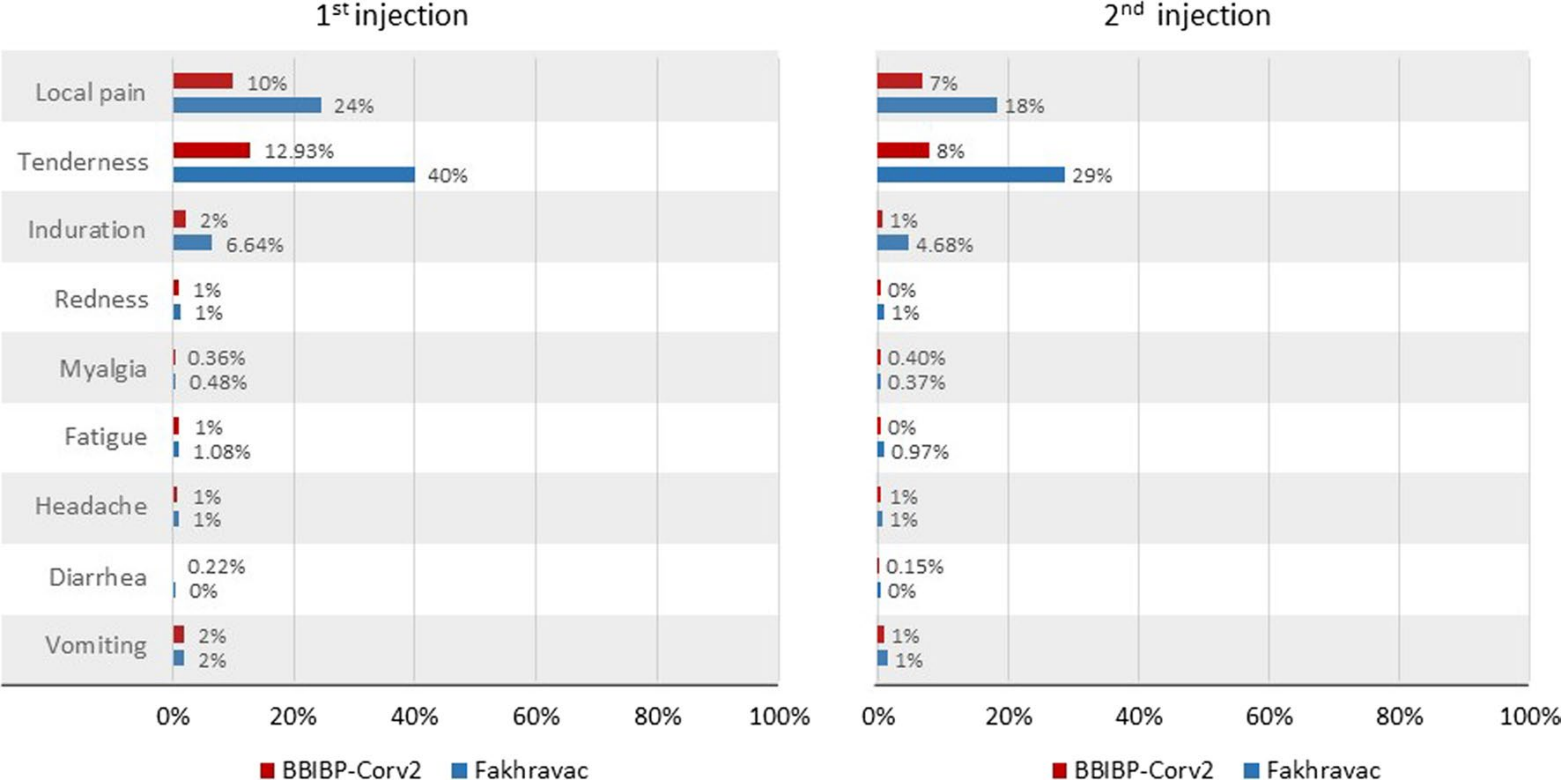
Local and systemic adverse events. This scheme contains grade 1 and grade 2 adverse events. Grade 3 and grade 4 adverse events have been reported as Serious Adverse Events (SAE)

The most common systemic adverse reaction was mild to moderate vomiting (grade I or II), which occurred in 2% of the participants after the first injection of either of the vaccines. The figure for the second injection was 1%. The only grade IV vomiting appeared in the context of an anaphylactic reaction which was referred to hospital A&E and discharged the same day. The second most prevalent systemic adverse reaction was mild to moderate fatigue, which occurred in about 1% or less of the participants in both vaccines and after both injections. The occurrence of other systemic reactions, such as myalgia, headache, and diarrhea, was rare in both groups (Fig. 4). We observed 3 cases of headaches that required narcotic painkillers (grade III) and three more that were referred for inpatient assessments (grade IV). Additional work-up was carried out on these patients, including brain CT-Scan, brain MRI, EEG, and Transcranial Doppler (TCD). All the results were normal, and the only one whose symptoms lasted more than a week was diagnosed with a migraine.

We recorded 9815 Medically Attendant Adverse Events (MAAEs) during four months of active follow-up. In the randomized arm, the rates of MAAE in the Fakhravac were slightly lower than the BBIBP-Corv2 groups (3.5 and 4 per 1000 person-day). The non-randomized arm, this rate was lower in the BBIBP-Corv2 group (3.2 and 1.9 per 1000 person-day). All MMAEs were followed, and their relationship to the vaccine injections was examined. The research team categorized 736 MAAEs as somehow related to the vaccines received. In the randomized arm, the rate of related MAAEs in the Fakhravac and BBIBP-Corv2 groups were 0.31 and 0.26 per 1000 person-day. The corresponding figures for the non-randomized arm were 0.24 and 0.07, which is lower in the BBIBP-Corv2 group. We observed 23 allergic events in the total follow-up period with a rate of 0.77 and 0.43 per 100,000 in the Fakhravac and BBIBP-Corv2 groups.

There were 73 Serious Adverse Events (SAEs), none of which were deemed to be related to the vaccines received. We observed slightly lower SAE rates in the Fakhravac (2.25, 95% CI 1.72–2.89) compared with the BBIBP-Corv2 (2.57, 95% CI 1.33–4.49) groups. We recorded eight deaths during the four-month active follow-up period because of intracranial hemorrhage, insulin poisoning, car accident (one each), and five heart attacks. Death due to heart attack in the study cohort was not significantly different from the general Iranian population [9] (SMR = 1.89, 95% CI 0.71–5.03 in the Fakhravac group and SMR = 1.97, 95% CI 0.82–4.74 in the BBIBP-Corv2).

## Discussion

We found that the Fakhravac inactivated SARS-CoV-2 vaccine is similar in safety and efficacy to the BBIBP-Corv2 vaccine. All observed local and systemic adverse reactions were generally self-limited and resolved completely. We observed a slightly higher occurrence of solicited local and systemic adverse reactions and a slightly lower rate of MAAEs and SAEs in the Fakhravac compared to BBIBP-Corv2 vaccine groups in the randomized arm. With 81.5% estimated efficacy, the Fakhravac vaccine was non-inferior to the reported 72.8% vaccine efficacy of BBIBP-Corv2 within the 10% non-inferiority margin after taking into account the 35% information fraction achieved during the study. In the non-randomized arm, the results were inconclusive. Overall, non-inferiority of the efficacy of the Fakhravac vaccine was demonstrated after adjusting for all possible confounding.

The non-randomized arm is prone to confounding and bias. Recruitment for this trial started in September 2021, when the BBIBP-Corv2 vaccine became available to the general Iranian population as part of the national Covid-19 vaccination program. This seriously impacted the willingness of the people to participate in the study, undergo randomization and remain unaware of the type of vaccine received. To overcome the slow recruitment, a change in design to allow the participants to choose the type of vaccine they receive became inevitable. Non-randomized and unblinded participants had less incentive to fill out the mobile application and inform the follow-up center about any change in the state of their health. The considerably lower incidence of symptomatic PCR + Covid-19 in both groups in the non-randomized arm reflects this less cooperative population, particularly in those receiving the BBIBP-Corv2 vaccine.

A changing pattern of the pandemic curve further complicated the recruitment and follow-up in the non-randomized arm [10]. It coincided with the decline in the epidemic curve of the Delta and the rising curve of the Omicron variants in Iran. Contrary to the randomized arm, where enrolment in the Fakhravac and BBIBP-Corv2 vaccine group occurred in blocks and therefore were time-matched, enrollment in the non-randomized arm did not follow a constant pattern and tended to be in clusters. Thus, the incidence of Covid-19 cases in the study groups in the non-randomized arm was greatly affected by their time of enrollment. We tried to overcome this important confounding factor by adjusting for the calendar week of receiving the first vaccine injection. However, any conclusion drawn from the non-randomized arm is likely to be affected by this limitation.

We can draw a non-inferiority efficacy conclusion for the Fakhravac vaccine just from our randomized arm. The number of events of the primary outcome in the calculated sample size was 365. Based on our conservative estimates of Covid-19 incidence in Iran, we needed to recruit 40,000 and follow them for six months to reach this number. However, the actual occurrence rates of Covid-19 were much higher than predicted, and we observed 855 cases, 129 (35% of 365 required by target sample size) of which occurred in the randomized arm. After adjusting the α error to 0.002 and confidence intervals (one-way) to 99.9% for 35% information fraction using the conservative O’Brien Fleming method, we were able to demonstrate the non-inferiority of the Fakhravac vaccine within the 10% margin from the reported 72.8% efficacy of the BBIBP-Corv2 vaccine (Table 2).

We found that those who received the Fakhravac vaccine are four times less likely to be hospitalized due to Covid-19 than the BBIBP-Corv2 vaccine, which agrees with our findings about symptomatic PCR + Covid-19 in the randomized arm. Given the telephone follow-up of those who had missed two successive weekly self-reports on their mobile application, we believe that the ascertainment of hospitalization due to Covid-19 is not affected by the degree of cooperation of the study participants and is likely to be valid.

We observed higher rates of grade I and II local and systemic adverse reactions in the Fakhravac compared to the BBIBP-Corv2 vaccine groups; however, they were all self-limited and were of little clinical value. The higher rates of solicited local and systemic reactions in the Fakhravac vaccine group could be partly attributed to the underreporting of these reactions in the unblinded, non-random BBIBP-Corv2 vaccine recipients. Furthermore, the figures in our study were generally lower than those reported for the BBIBP-Corv2 vaccine [6] (17% in the currents study versus 44.2% originally reported for BBIBP-Corv2). Other vaccine platforms also reported higher rates of adverse reactions [11, 12]. Some rare grade III and IV local and systemic adverse reactions were detected and resolved completely.

We observed a slightly lower rate of MAAEs and SAEs in the Fakhravac compared to the BBIBP-Corv2 vaccine groups. The standardized Mortality Ratio (SMR) for cardiovascular deaths was not significantly different from the general Iranian population. The details of all SAEs are included in the Additional file 1.

In summary, our study shows that the efficacy of the Fakhravac vaccine is non-inferior to the BBIBP-Corv2 vaccine, and the safety profile of both vaccines is similar. Therefore, Fakhravac is a reliable and safe addition to our global arsenal of vaccines against Covid-19.

## Limitation

First of all, the study was initially designed as an RCT, but due to the dissatisfaction of the volunteers with the blindness and randomness of the study, the study design was changed to a comprehensive cohort study. Second, the study did not include pregnant women or those younger than 18 years; thus, the efficacy and safety of the inactivated vaccines in these groups remain unknown. Third, the trial was mainly conducted on generally healthy Iranian people. There was insufficient power to test the efficacy among those with chronic diseases, older adults, and those in other geographic populations. Fourth, there were only two severe cases of Covid-19, so we cannot make conclusions about preventing severe cases.

## Conclusion

Fakhravac vaccine is safe and effective as the Sinopharm vaccine in preventing symptomatic PCR-positive covid-19 disease. In another way, we could prove the non-inferiority of Fakhravac to the BBIBP-Corv2 vaccine. However, the Fakhravac vaccine is significantly more effective than the Sinopharm vaccine in preventing hospitalization due to Covid19 symptomatic disease.

## Supplementary Information

**Additional file 1.** SMR analysis for death due to heart attack is included in the supplementary file.

## Data Availability

The datasets used and/or analysed during the current study are available from the corresponding author on reasonable request.
