# Supplementary material for: Safety and efficacy of the FAKHRAVAC compared with BBIBP-Corv2 against SARS-CoV-2 in adults: a non-inferiority multi-center trial
Source: Virol J. 2023 Jul 18;20:154. doi: 10.1186/s12985-023-02121-z (PMC10355035; doi:10.1186/s12985-023-02121-z)
Supplement: Supplementary file 1 — Additional file 1. SMR analysis for death due to heart attack is included in the supplementary file. [file 12985_2023_2121_MOESM1_ESM.docx]

Supplement

Tables and figures of the final clinical report

Safety and efficacy of the FAKHRAVAC compared with BBIBP-Corv2 against SARS-CoV-2 in adults: a non-inferiority multi-center trial

[Index of tables 2](#_Toc135502751)

[Index of figures 4](#_Toc135502752)

[Participants’ flow in the study 6](#_Toc135502753)

[Exclusions in the online screening 7](#_Toc135502754)

[Withdrawals before the second injection 7](#_Toc135502755)

[Baseline comparisons 8](#_Toc135502756)

[Comparison of the participants’ demographic characteristics 8](#_Toc135502757)

[Comparison of the participants’ vital signs before injection 10](#_Toc135502758)

[Comparison of the participants’ comorbidities 11](#_Toc135502759)

[Results: Efficacy outcomes 12](#_Toc135502760)

[The non-inferiority margin for the primary outcome 12](#_Toc135502761)

[Symptomatic Covid-19 with positive PCR 12](#_Toc135502762)

[Symptomatic Covid-19 with positive PCR in Random arm 13](#_Toc135502763)

[Symptomatic Covid-19 with positive PCR in Non-Random arm 18](#_Toc135502764)

[Symptomatic Covid-19 with positive PCR – Total 22](#_Toc135502765)

[Schematic summary of the survival analysis results of symptomatic Covid-19 with positive PCR 24](#_Toc135502766)

[Symptomatic Covid-19 leading to hospitalization 25](#_Toc135502767)

[Symptomatic Covid-19 with positive PCR leading to hospitalization in Random arm 26](#_Toc135502768)

[Symptomatic Covid-19 with positive PCR leading to hospitalization in Non-Random arm 29](#_Toc135502769)

[Results: Safety outcomes 31](#_Toc135502770)

[Serious Adverse Event (SAE): Deaths 31](#_Toc135502771)

[SMR analysis for death due to heart attack 31](#_Toc135502772)

[Serious Adverse Event (SAE): Hospitalizations 33](#_Toc135502773)

[Serious Adverse Event (SAE) - hospitalizations due to Covid-19 33](#_Toc135502774)

[Serious Adverse Event (SAE) - hospitalizations due to surgery 34](#_Toc135502775)

[Serious Adverse Event (SAE) - hospitalizations due to medical disorders 35](#_Toc135502776)

[Serious Adverse Event (SAE) - hospitalizations due to cardiovascular disorders 36](#_Toc135502777)

[Solicited local adverse reactions 38](#_Toc135502778)

[Solicited systemic adverse reactions 50](#_Toc135502779)

[List of all adverse events during four month active follow-up 69](#_Toc135502780)

[Withdrawal from the study after receiving the vaccine 91](#_Toc135502781)

## Index of tables

[Table 1 Numbers and reasons for exclusions in the online screening 7](#_Toc116718153)

[Table 2 Reason for withdrawals before the second injection 7](#_Toc116718154)

[Table 3 Demographic characteristics of the participants 8](#_Toc116718155)

[Table 4 Comparison of demographic characteristics in the study arms 9](#_Toc116718156)

[Table 5 Comparison of age groups in study arms 10](#_Toc116718157)

[Table 6 Vital signs before injection 10](#_Toc116718158)

[Table 7 Comparison of the participants’ comorbidities 11](#_Toc116718159)

[Table 8 Examining the severity of symptomatic Covid-19 with positive PCR from 14 days after the second injection by study groups 12](#_Toc116718160)

[Table 9 Logrank test for occurrence of symptomatic Covid-19 with positive PCR 14 days after the second injection in Random arm 13](#_Toc116718161)

[Table 10 Assessment of PH assumption 14 days after the second injection in Random arm 15](#_Toc116718162)

[Table 11 Cox regression for occurrence of symptomatic Covid-19 with positive PCR 14 days after the second injection in Random arm 15](#_Toc116718163)

[Table 12 Cox regression for occurrence of symptomatic Covid-19 with positive PCR 14 days after the second injection (participants with Covid-19 history eliminated) in Random arm 16](#_Toc116718164)

[Table 13 Logrank test for occurrence of symptomatic Covid-19 with positive PCR 14 days after the second injection in Non-Random arm 17](#_Toc116718165)

[Table 14 Assessment of PH assumption 14 days after the second injection in Non-Random arm 19](#_Toc116718166)

[Table 15 Cox regression for occurrence of symptomatic Covid-19 with positive PCR 14 days after the second injection in Non-Random arm 19](#_Toc116718167)

[Table 16 Cox regression for occurrence of symptomatic Covid-19 with positive PCR 14 days after the second injection (participants with Covid-19 history eliminated) in Non-Random arm 20](#_Toc116718168)

[Table 17 Logrank test for occurrence of symptomatic Covid-19 with positive PCR 14 days after the second injection – Total 21](#_Toc116718169)

[Table 18Cox regression for occurrence of symptomatic Covid-19 with positive PCR 14 days after the second injection - Total 22](#_Toc116718170)

[Table 19Cox regression for occurrence of symptomatic Covid-19 with positive PCR 14 days after the second injection (participants with Covid-19 history eliminated) - Total 22](#_Toc116718171)

[Table 20 Examining the severity of symptomatic Covid-19 with positive PCR that leading to hospitalization from 14 days after the second injection by study groups 24](#_Toc116718172)

[Table 21 Logrank test for occurrence of symptomatic Covid-19 with positive PCR leading to hospitalization 14 days after the second injection in Random arm 25](#_Toc116718173)

[Table 22. Assessment of PH assumption for occurrence of symptomatic Covid-19 with positive PCR leading to hospitalization 14 days after the second injection in Random arm 27](#_Toc116718174)

[Table 23 Logrank test for occurrence of symptomatic Covid-19 with positive PCR leading to hospitalization 14 days after the second injection in NON-Random arm 28](#_Toc116718175)

[Table 24 Cox regression for occurrence of symptomatic Covid-19 with positive PCR leading to hospitalization 14 days after the second injection in Non-Random arm 29](#_Toc116718176)

[Table 25 Cox regression for occurrence of symptomatic Covid-19 with positive PCR leading to hospitalization (participants with Covid-19 history eliminated) 14 days after the second injection in Non-Random arm 29](#_Toc116718177)

[Table 26 List of participants died during the four month active follow-up 30](#_Toc116718178)

[Table 27 Comparison of observed and expected number of deaths from heart attack in the study arms 30](#_Toc116718179)

[Table 28 Classification of Serious Adverse Events (SAEs) by cause 32](#_Toc116718180)

[Table 29 List of hospitalizations due to Covid-19 32](#_Toc116718181)

[Table 30 List of hospitalizations due to surgery 33](#_Toc116718182)

[Table 31 List of hospitalizations due to medical disorders 34](#_Toc116718183)

[Table 32 List of hospitalizations due to cardiovascular disorders 35](#_Toc116718184)

[Table 33 Pain local reaction during first week after first injection 37](#_Toc116718185)

[Table 34 Pain local reaction during first week after second injection 39](#_Toc116718186)

[Table 35 Tenderness local reaction during first week after first injection 41](#_Toc116718187)

[Table 36 Tenderness local reaction during first week after second injection 43](#_Toc116718188)

[Table 37 Redness local reaction during first week after first injection 45](#_Toc116718189)

[Table 38 Redness local reaction during first week after second injection 46](#_Toc116718190)

[Table 39 Induration local reaction during first week after first injection 47](#_Toc116718191)

[Table 40 Induration local reaction during first week after second injection 48](#_Toc116718192)

[Table 41 Vomiting systemic reaction during first week after first injection 49](#_Toc116718193)

[Table 42 Vomiting systemic reaction during first week after second injection 51](#_Toc116718194)

[Table 43 Diarrhea systemic reaction during first week after first injection 52](#_Toc116718195)

[Table 44 Diarrhea systemic reaction during first week after second injection 54](#_Toc116718196)

[Table 45 Headache systemic reaction during first week after first injection 56](#_Toc116718197)

[Table 46 Headache systemic reaction during first week after second injection 58](#_Toc116718198)

[Table 47 Fatigue systemic reaction during first week after first injection 60](#_Toc116718199)

[Table 48 Fatigue systemic reaction during first week after second injection 62](#_Toc116718200)

[Table 49 Muscle pain systemic reaction during first week after first injection 64](#_Toc116718201)

[Table 50 Muscle pain systemic reaction during first week after second injection 66](#_Toc116718202)

[Table 51 Final classification of adverse events that are judged by the follow-up team to be somehow related to the vaccines recieved during four month active follow-up in study groups 68](#_Toc116718203)

[Table 52 All allergic related adverse events during four month active follow-up in study groups 69](#_Toc116718204)

[Table 53 Final classification of all adverse events by the first three letters of the ICD-10 code during four month active follow-up in study groups 70](#_Toc116718205)

[Table 54 Final classification of all adverse events during four month active follow-up in study groups 78](#_Toc116718206)

[Table 55 Reasons for withdrawal from the study by study arm 90](#_Toc116718207)

## Index of figures

[Figure 1 Participants’ flow diagram (has been included in the manuscript) 6](file:///C:\Users\DELL\Desktop\Articles%202\submit%20Phase%20III\Supplementary.docx#_Toc116718141)

[Figure 2 Kaplan-Meier curve for occurrence of symptomatic Covid-19 with positive PCR 14 days after the second injection in Random arm 13](#_Toc116718142)

[Figure 3 Checking proportional hazard assumption 14 days after the second in Random arm 14](#_Toc116718143)

[Figure 4 Schonefeld residual against time 14 days after the second injection in Random arm 15](#_Toc116718144)

[Figure 5 Kaplan-Meier curve for occurrence of symptomatic Covid-19 with positive PCR 14 days after the second injection in Non-Random arm 17](#_Toc116718145)

[Figure 6 Checking proportional hazard assumption 14 days after the second in Non-Random arm 18](#_Toc116718146)

[Figure 7 Schonefeld residual against time 14 days after the second injection in Non-Random arm 19](#_Toc116718147)

[Figure 8 Kaplan-Meier curve for occurrence of symptomatic Covid-19 with positive PCR 14 days after the second injection – Total 21](#_Toc116718148)

[Figure 9 The hazard ratio of the Fakhravac compared to the BBIBP-Corv2 in PCR-positive Covid-19 cases (has been included in the manuscript) 23](#_Toc116718149)

[Figure 10 Kaplan-Meier curve for occurrence of symptomatic Covid-19 with positive PCR leading to hospitalization 14 days after the second injection in Random arm 25](#_Toc116718150)

[Figure 11 Checking proportional hazard assumption for occurrence of symptomatic Covid-19 with positive PCR leading to hospitalization 14 days after the second injection in Random arm 26](#_Toc116718151)

[Figure 12 Kaplan-Meier curve for occurrence of symptomatic Covid-19 with positive PCR leading to hospitalization 14 days after the second injection in Non-Random arm 28](#_Toc116718152)

# Participants’ flow in the study

Figure 1 Participants’ flow diagram (has been included in the manuscript)

37982 Successfully passed the online eligibility assessment

13140 Did not attend for additional face-to-face screening

688 Were unwilling to use contraception for six months

55 Had current Covid-19 illness

12 Had history of severe allergic reactions

7 Were breast feeding

6 Used anticoagulants medications

4 Withdrew consent (did not sign written informed consent)

4 Used immunosuppressive medications

3 Had serious neurological disorders

2 Had received other Covid-19 vaccine

2 Had history of severe hematologic disorders

2 Were alcohol or drug abuser

1 Was pregnant

1656 Underwent randomization

22400 Received vaccine based on preference

24056 Were eligible for enrollment

Were Included in the intention-to-treat and safety analysis

2054 Were withdrawn:

2 Serious adverse reactions

10 Became pregnant

11 Received another vaccine

2 Deaths

125 Withdrew consent

88 Covid-19 diagnosed

1816 Lost to follow-up

2350 Received 2nd BBIBP-Corv2 dose

Were included in the modified intention-to-treat analysis

17375 Received 2nd Fakhravac dose

Were included in the modified intention-to-treat analysis

780 Received 2^nd^ BBIBP-Corv2 dose

Were included in the modified intention-to-treat analysis

770 Received 2^nd^ Fakhravac dose

Were included in the modified intention-to-treat analysis

54 Were withdrawn:

3 Received another vaccine

11 Withdrew consent

16 Covid-19 diagnosed

24 Lost to follow-up

52 Were withdrawn:

1 Received another vaccine

7 Withdrew consent

1 Death

10 Covid-19 diagnosed

33 Lost to follow-up

621 Were withdrawn:

2 Became pregnant

7 Received another vaccine

35 Withdrew consent

15 Covid-19 diagnosed

562 Lost to follow-up

2971 Received 1^st^ BBIBP-Corv2 dose

19429 Received 1^st^ Fakhravac dose

824 Received 1^st^ Fakhravac dose

832 Received 1^st^ BBIBP-Corv2 dose

## Exclusions in the online screening

Table 1 Numbers and reasons for exclusions in the online screening

| N | Reason for exclusion | N | Reason for exclusion |
| --- | --- | --- | --- |
| 1005 | History of severe bleeding after injection | 15982 | Receiving another Covid-19 vaccine |
| 2109 | History of long-time use of Corticosteroid | 491 | Blood transfusion |
| 890 | History of long-time use of Anticoagulantss | 350 | HIV infection |
| 867 | Addiction to Opium or Alcohol | 883 | Cancer |
| 3266 | Severe allergic reactions to drugs and vaccines | 942 | History of uncontrolled severe psychiatric disorders |
| 6842 | Tending to pregnancy | 2588 | History of hematologicc disorders |

## Withdrawals before the second injection

Table 2 Reason for withdrawals before the second injection

| Total | BBIBP-Corv2 Non-Random | Fakhravac Non-Random | BBIBP-Corv2 Random | Fakhravac Random | Reasons for withdrawals |
| --- | --- | --- | --- | --- | --- |
| 2 | 0 | 2 | 0 | 0 | Serious reaction to vaccine injection (Systemic urticaria) |
| 12 | 2 | 10 | 0 | 0 | Became pregnant |
| 22 | 7 | 11 | 1 | 3 | Received another vaccine |
| 178 | 35 | 125 | 7 | 11 | Withdrew consent |
| 2435 | 562 | 1816 | 33 | 24 | Lost to follow-up |
| 3 | 0 | 2 | 1 | 0 | Death |
| 129 | 15 | 88 | 10 | 16 | Covid-19 diagnosed |
| 2781 | **621** | **2054** | **52** | **54** | **Total** |

# Baseline comparisons

## Comparison of the participants’ demographic characteristics

Table 3 Demographic characteristics of the participants

| Characteristic | Overall, N = 24,056^1^ |
| --- | --- |
| **Sex** |  |
| Female | 11,099 (46%) |
| Male | 12,952 (54%) |
| **Age (Year)** |  |
| 18 - 30 Years | 7,301 (30%) |
| 30 - 40 Years | 8,649 (36%) |
| 40 - 50 Years | 5,455 (23%) |
| 50 - 60 Years | 1,945 (8.1%) |
| 60+ Years | 698 (2.9%) |
| **BMI** | 25.3 (22.7, 28.3) |
| **Education** |  |
| Elementary | 725 (3.0%) |
| Under Diploma | 3,166 (13%) |
| Diploma | 8,350 (35%) |
| Diploma Plus | 2,131 (8.9%) |
| Bachelor | 7,306 (30%) |
| Master | 2,014 (8.4%) |
| Doctoral and above | 358 (1.5%) |
| ^1^n (%); Median (IQR) |  |

Table 4 Comparison of demographic characteristics in the study arms

| Characteristic | Fakhra, Random,  N = 824^1^ | BBIBP-Corv2, Random,  N = 832^1^ | Fakhravac,  Non-Random,  N = 19,429^1^ | BBIBP-Corv2,  Non-Random,  N = 2,971^1^ |
| --- | --- | --- | --- | --- |
| **Sex** |  |  |  |  |
| Female | 306 (37%) | 310 (37%) | 9,216 (47%) | 1,267 (43%) |
| Male | 518 (63%) | 522 (63%) | 10,210 (53%) | 1,702 (57%) |
| **Age (Year)** |  |  |  |  |
| 18 - 30 Years | 297 (36%) | 309 (37%) | 5,763 (30%) | 932 (31%) |
| 30 - 40 Years | 351 (43%) | 334 (40%) | 6,982 (36%) | 982 (33%) |
| 40 - 50 Years | 152 (18%) | 153 (18%) | 4,447 (23%) | 703 (24%) |
| 50 - 60 Years | 23 (2.8%) | 31 (3.7%) | 1,647 (8.5%) | 244 (8.2%) |
| 60+ Years | 1 (0.1%) | 5 (0.6%) | 584 (3.0%) | 108 (3.6%) |
| **BMI** | 25.6 (22.9, 29.1) | 25.2 (22.8, 28.4) | 25.4 (22.7, 28.4) | 24.6 (22.9, 27.2) |
| **Education** |  |  |  |  |
| Elementary | 17 (2.1%) | 18 (2.2%) | 543 (2.8%) | 147 (5.0%) |
| Under Diploma | 78 (9.5%) | 70 (8.4%) | 2,487 (13%) | 531 (18%) |
| Diploma | 282 (34%) | 290 (35%) | 6,708 (35%) | 1,070 (36%) |
| Diploma Plus | 74 (9.0%) | 87 (10%) | 1,746 (9.0%) | 224 (7.5%) |
| Bachelor | 262 (32%) | 267 (32%) | 6,003 (31%) | 774 (26%) |
| Master | 91 (11%) | 84 (10%) | 1,667 (8.6%) | 172 (5.8%) |
| Doctoral and above | 19 (2.3%) | 16 (1.9%) | 272 (1.4%) | 51 (1.7%) |
| **History of Covid-19** | |  |  |  |
| Yes | 11 (1.34%) | 14 (1.68%) | 1702 (8.76%) | 55 (1.85%) |
| No | 812 (98.66%) | 818 (98.32%) | 17723 (91.24%) | 2914 (98.15%) |

Table 5 Comparison of age groups in study arms

| Age group | Fakhravac | BBIBP-Corv2 | Total |
| --- | --- | --- | --- |
| 18-30 | 6060 | 1261 | 7321 |
|  | 29.93 | 32.99 | 30.41 |
|  |  |  |  |
| 30-40 | 7333 | 1316 | 8649 |
|  | 36.21 | 34.43 | 35.93 |
|  |  |  |  |
| 40-50 | 4601 | 857 | 5458 |
|  | 22.72 | 22.42 | 22.67 |
|  |  |  |  |
| 50-60 | 1670 | 275 | 1945 |
|  | 8.25 | 7.20 | 8.08 |
|  |  |  |  |
| 60+ | 585 | 113 | 698 |
|  | 2.89 | 2.96 | 2.90 |
|  |  |  |  |
| Total | 20249 | 3822 | 24071 |
|  | 100 | 100 | 100 |

## Comparison of the participants’ vital signs before injection

Table 6 Vital signs before injection

| Characteristic | Overall,  N = 24,056^1^ | Fakhravac, Random,  N = 824^1^ | BBIBP-Corv2, Random,  N = 832^1^ | Fakhravac,  Non-Random,  N = 19,429^1^ | BBIBP-Corv2,  Non-Random,  N = 2,971^1^ |
| --- | --- | --- | --- | --- | --- |
| **Body Temprature** | 36.60 (36.40, 36.70) | 36.60 (36.30, 36.75) | 36.50 (36.30, 36.70) | 36.60 (36.40, 36.70) | 36.60 (36.50, 36.70) |
| **Diastolic Blood Pressure** | 81 (76, 89) | 86 (78, 95) | 86 (77, 96) | 82 (76, 89) | 80 (77, 87) |
| **Systolic Blood Pressure** | 120 (113, 130) | 120 (111, 130) | 121 (111, 132) | 120 (112, 130) | 120 (116, 128) |
| **Respiratory Rate** | 16 (14, 18) | 18 (16, 20) | 18 (16, 20) | 16 (14, 17) | 16 (15, 18) |
| **Heart Rate** | 80 (74, 85) | 80 (74, 86) | 80 (74, 86) | 80 (73, 85) | 80 (75, 83) |
| ^1^Median (IQR) | | | | | |

## Comparison of the participants’ comorbidities

Table 7 Comparison of the participants’ comorbidities

| Characteristic | Overall,  N = 24,056 | Fakhravac, Random,  N = 824 | BBIBP-Corv2, Random,  N = 832 | Fakhravac,  Non-Random,  N = 19,429 | BBIBP-Corv2,  Non-Random,  N = 2,971 |
| --- | --- | --- | --- | --- | --- |
| **High blood pressure** | 648 (2.7%, N: 24,041) | 14 (1.7%, N: 824) | 21 (2.5%, N: 831) | 541 (2.8%, N: 19,418) | 72 (2.4%, N: 2,968) |
| **Chronic heart disease** | 170 (0.7%, N: 24,041) | 5 (0.6%, N: 824) | 10 (1.2%, N: 831) | 135 (0.7%, N: 19,418) | 20 (0.7%, N: 2,968) |
| **Chronic non-asthma lung disease** | 118 (0.5%, N: 24,041) | 1 (0.1%, N: 824) | 2 (0.2%, N: 831) | 102 (0.5%, N: 19,418) | 13 (0.4%, N: 2,968) |
| **Asthma** | 144 (0.6%, N: 24,041) | 6 (0.7%, N: 824) | 6 (0.7%, N: 831) | 118 (0.6%, N: 19,418) | 14 (0.5%, N: 2,968) |
| **Chronic kidney disease** | 76 (0.3%, N: 24,041) | 1 (0.1%, N: 824) | 2 (0.2%, N: 831) | 59 (0.3%, N: 19,418) | 14 (0.5%, N: 2,968) |
| **Moderate / severe liver disease** | 48 (0.2%, N: 24,041) | 1 (0.1%, N: 824) | 0 (0%, N: 831) | 42 (0.2%, N: 19,418) | 5 (0.2%, N: 2,968) |
| **(Fatty liver) Mild liver disease** | 546 (2.3%, N: 24,041) | 27 (3.3%, N: 824) | 36 (4.3%, N: 831) | 453 (2.3%, N: 19,418) | 30 (1.0%, N: 2,968) |
| **Chronic neurological disease** | 187 (0.8%, N: 24,041) | 6 (0.7%, N: 824) | 9 (1.1%, N: 831) | 146 (0.8%, N: 19,418) | 26 (0.9%, N: 2,968) |
| **Diabetes without omplications** | 403 (1.7%, N: 24,041) | 7 (0.8%, N: 824) | 10 (1.2%, N: 831) | 340 (1.8%, N: 19,418) | 46 (1.5%, N: 2,968) |
| **Diabetes with complications** | 65 (0.3%, N: 24,041) | 0 (0%, N: 824) | 1 (0.1%, N: 831) | 54 (0.3%, N: 19,418) | 10 (0.3%, N: 2,968) |
| **Chronic blood disease** | 149 (0.6%, N: 24,041) | 6 (0.7%, N: 824) | 5 (0.6%, N: 831) | 127 (0.7%, N: 19,418) | 11 (0.4%, N: 2,968) |
| **Rheumatic diseases** | 97 (0.4%, N: 24,041) | 1 (0.1%, N: 824) | 3 (0.4%, N: 831) | 81 (0.4%, N: 19,418) | 12 (0.4%, N: 2,968) |
| **Dementia** | 28 (0.1%, N: 24,041) | 0 (0%, N: 824) | 0 (0%, N: 831) | 26 (0.1%, N: 19,418) | 2 (<0.1%, N: 2,968) |

# Results: Efficacy outcomes

## The non-inferiority margin for the primary outcome

We used a 10% non-inferiority margin to the reported BBIBP-Corv2 vaccine efficacy of 72.8% (i.e., a minimum 62.8% vaccine efficacy for Fakhravac) which was equivalent to assuming an upper boundary of 1.35 for the hazard ratio of acquiring symptomatic PCR+ covid-19 in Fakhravac recipients compared to BBIBP-Corv2 vaccine recipients.

The details of how a 10% non-inferiority margin to 72.8% Sinopharm vaccine efficacy translated to a hazard ratio of 1.35 are as following:

$$\boldsymbol{VE=1-HR \to HR=1-VE}$$

$$\boldsymbol{VE}\left( \mathbf{BBIBP} \right)\mathbf{=1}\mathbf{-}\mathbf{0.728=0.282}\boldsymbol{\to}\mathbf{HR}\left( \frac{\mathbf{Hazard BBIBP}}{\mathbf{Hazard Placebo}} \right)\mathbf{=0.282}$$

$$\boldsymbol{VE}\left( \mathbf{Fakhravac} \right)\mathbf{=1}\mathbf{-}\mathbf{0.628=0.382}\boldsymbol{\to}\mathbf{HR}\left( \frac{\mathbf{Hazard Fakhravac}}{\mathbf{Hazard Placebo}} \right)\mathbf{=0.382}$$

$$\boldsymbol{HR}\left( \frac{\mathbf{Hazard Fakhravac}}{\mathbf{Hazard BBIBP}} \right)\boldsymbol{=}\frac{\boldsymbol{0.382}}{\boldsymbol{0.282}}\boldsymbol{=1.354}$$

## Symptomatic Covid-19 with positive PCR

Table 8 Examining the severity of symptomatic Covid-19 with positive PCR from 14 days after the second injection by study groups

|  | Fakhravac, Random  (N: 53) | BBIBP-Corv2, Random  (N: 76) | Fakhravac,  Non-Random  (N: 662) | BBIBP-Corv2, Non-Random  (N: 64) | Fakhravac  (N: 715) | BBIBP-Corv2  (N: 140) |
| --- | --- | --- | --- | --- | --- | --- |
| Fever | 12 (22.64%) | 41 (53.95%) | 195 (29.46%) | 28 (43.75%) | 207 (28.95%) | 69 (49.29%) |
| Cough | 27 (50.94%) | 51 (67.11%) | 369 (55.74%) | 45 (70.31%) | 396 (55.38%) | 96 (68.57%) |
| Shortness Breath | 3 (5.66%) | 19 (25%) | 49 (7.4%) | 14 (21.88%) | 52 (7.27%) | 33 (23.57%) |
| Fatigue | 21 (39.62%) | 45 (59.21%) | 326 (49.24%) | 29 (45.31%) | 347 (48.53%) | 74 (52.86%) |
| Muscule Pain | 19 (35.85%) | 36 (47.37%) | 273 (41.24%) | 29 (45.31%) | 292 (40.84%) | 65 (46.43%) |
| Headache | 11 (20.75%) | 35 (46.05%) | 199 (30.06%) | 22 (34.38%) | 210 (29.37%) | 57 (40.71%) |
| Anosmia | 4 (7.55%) | 14 (18.42%) | 75 (11.33%) | 9 (14.06%) | 79 (11.05%) | 23 (16.43%) |
| SoreT hroat Coryza Congestion | 43 (81.13%) | 53 (69.74%) | 497 (75.08%) | 41 (64.06%) | 540 (75.52%) | 94 (67.14%) |
| Nausea/ Vomiting/ Diarrhea | 5 (9.43%) | 12 (15.79%) | 28 (4.23%) | 6 (9.38%) | 33 (4.62%) | 18 (12.86%) |
| Outpatient Refferal | 13 (24.53%) | 25 (32.89%) | 135 (20.39%) | 20 (31.25%) | 148 (20.7%) | 45 (32.14%) |
| Hospitalization | 0 (0%) | 5 (6.58%) | 11 (1.66%) | 3 (4.69%) | 11 (1.54%) | 8 (5.71%) |
| ICU Admission | 0 (0%) | 0 (0%) | 2 (0.3%) | 0 (0%) | 2 (0.28%) | 0 (0%) |
| UsingVentilator | 0 (0%) | 2 (2.63%) | 5 (0.76%) | 1 (1.56%) | 5 (0.7%) | 3 (2.14%) |
| MultiOrganFailure | 0 (0%) | 0 (0%) | 2 (0.3%) | 1 (1.56%) | 2 (0.28%) | 1 (0.71%) |
| AKI | 0 (0%) | 0 (0%) | 3 (0.45%) | 0 (0%) | 3 (0.42%) | 0 (0%) |
| AcuteLiverFailure | 1 (1.89%) | 0 (0%) | 3 (0.45%) | 1 (1.56%) | 4 (0.56%) | 1 (0.71%) |
| AcuteLVRVFailure | 1 (1.89%) | 0 (0%) | 3 (0.45%) | 1 (1.56%) | 4 (0.56%) | 1 (0.71%) |
| SepticShock | 2 (3.77%) | 0 (0%) | 3 (0.45%) | 0 (0%) | 5 (0.7%) | 0 (0%) |
| IschemicHemorrhagicStroke | 0 (0%) | 1 (1.32%) | 5 (0.76%) | 0 (0%) | 5 (0.7%) | 1 (0.71%) |
| AMIDVTPE | 0 (0%) | 2 (2.63%) | 9 (1.36%) | 0 (0%) | 9 (1.26%) | 2 (1.43%) |
| Roommate | 1 (1.89%) | 8 (10.53%) | 36 (5.44%) | 11 (17.19%) | 37 (5.17%) | 19 (13.57%) |
| Mean O2 Saturation | 95.83 | 95.64 | 95.96 | 96 | 96 | 96 |
| Lung Infection Severity: n(mean%) | 4 (15%) | 8 (9%) | 17 (12%) | 1 (0%) | 21 (12%) | 9 (8%) |

### Symptomatic Covid-19 with positive PCR in Random arm


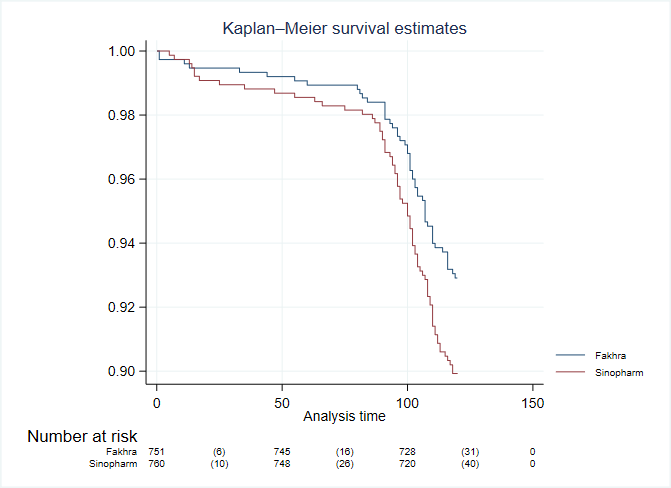


Figure 2 Kaplan-Meier curve for occurrence of symptomatic Covid-19 with positive PCR 14 days after the second injection in Random arm

Table 9 Logrank test for occurrence of symptomatic Covid-19 with positive PCR 14 days after the second injection in Random arm

| P-Value log rank | Incidence Rate per 1,000  (95 % CI) | Expected event | Event | person-time | Study groups |
| --- | --- | --- | --- | --- | --- |
| 0.0379 | 0.60  (0.46 - 0.78) | 64.78 | 53 | 88415 | Fakhravac |
|  | 0.86  (0.69 - 1.08) | 64.22 | 76 | 88453 | BBIBP-Corv2 |


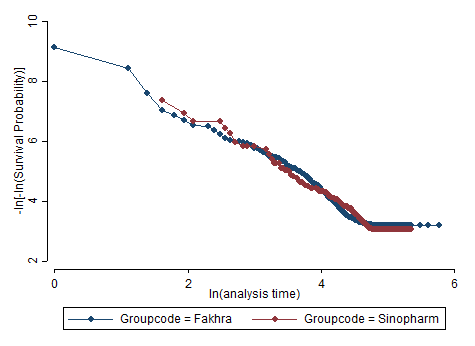


Figure 3 Checking proportional hazard assumption 14 days after the second in Random arm


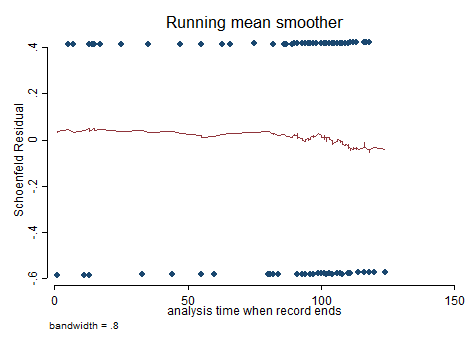


Figure 4 Schonefeld residual against time 14 days after the second injection in Random arm

Table 10 Assessment of PH assumption 14 days after the second injection in Random arm

| P value | df | Chi 2 |  |
| --- | --- | --- | --- |
| 0.66 | 1 | 0.19 | PH assumption |

Table 11 Cox regression for occurrence of symptomatic Covid-19 with positive PCR 14 days after the second injection in Random arm

| Adjusted HR**  (95.0% CI) | Adjusted HR*  (95.0% CI) | Unadjusted HR  (95.0 % CI) | Study groups |
| --- | --- | --- | --- |
| 1 | **1** | 1 | Fakhravac |
| 1.52  (1.07 – 2.17) | **1.48**  **(1.04 – 2.11)** | 1.45  (1.02 – 2.05) | BBIBP-Corv2 |

*Adjusted for age, sex and education

**Adjusted for age, sex, education and history of covid-19

Table 12 Cox regression for occurrence of symptomatic Covid-19 with positive PCR 14 days after the second injection (participants with Covid-19 history eliminated) in Random arm

| Adjusted HR**  (95.0% CI) | Adjusted HR*  (95.0% CI) | Unadjusted HR  (95.0 % CI) | Study groups |
| --- | --- | --- | --- |
| 1 | 1 | 1 | Fakhravac |
| 0.78  (0.60 – 1.01) | 0.74  (0.57 – 0.96) | 0.69  (0.53 – 0.90) | BBIBP-Corv2 |

*Adjusted for age, sex and education

**Adjusted for age, sex, education and week

### Symptomatic Covid-19 with positive PCR in Non-Random arm


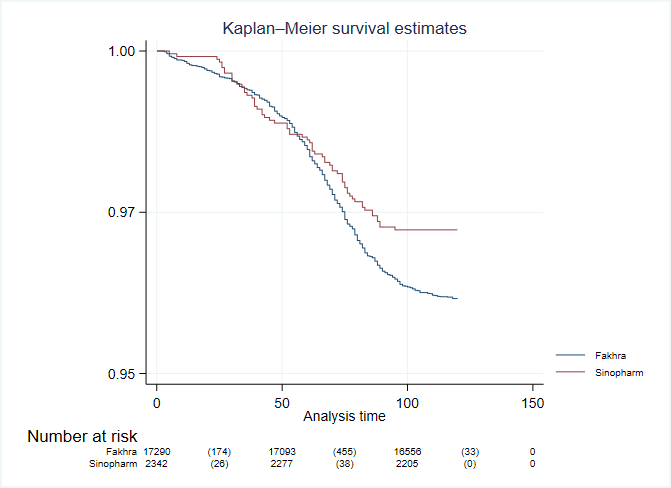


Figure 5 Kaplan-Meier curve for occurrence of symptomatic Covid-19 with positive PCR 14 days after the second injection in Non-Random arm

Table 13 Logrank test for occurrence of symptomatic Covid-19 with positive PCR 14 days after the second injection in Non-Random arm

| P-Value log rank | Incidence Rate per 1,000  (95 % CI) | Expected event | Event | person-time | Study groups |
| --- | --- | --- | --- | --- | --- |
| 0.0125 | 0.33  (0.30 - 0.35) | 640.29 | 662 | 2031463 | Fakhra |
|  | 0.24  (0.18 - 0.30) | 85.71 | 64 | 272252 | BBIBP-Corv2 |


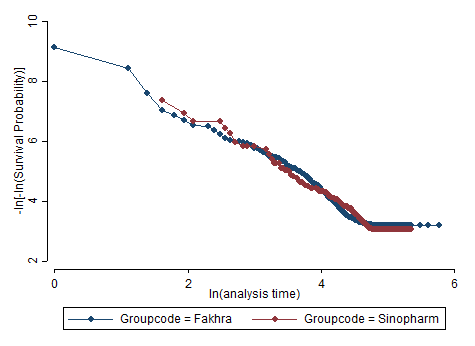


Figure 6 Checking proportional hazard assumption 14 days after the second in Non-Random arm


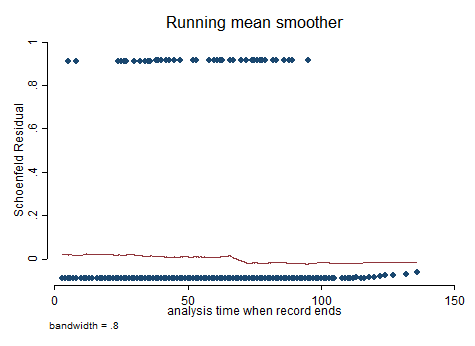


Figure 7 Schonefeld residual against time 14 days after the second injection in Non-Random arm

Table 14 Assessment of PH assumption 14 days after the second injection in Non-Random arm

| P value | df | Chi 2 |  |
| --- | --- | --- | --- |
| 0.0253 | 1 | 5 | PH assumption |

Table 15 Cox regression for occurrence of symptomatic Covid-19 with positive PCR 14 days after the second injection in Non-Random arm

| Adjusted HR***  (95.0% CI) | Adjusted HR**  (95.0% CI) | Adjusted HR*  (95.0% CI) | Unadjusted HR  (95.0 % CI) | Study groups |
| --- | --- | --- | --- | --- |
| 1 | **1** | 1 | 1 | Fakhravac |
| 0.81  (0.62 – 1.04) | **0.80**  **(0.62 – 1.03)** | 0.76  (0.59 – 0.99) | 0.72  (0.56 – 0.93) | BBIBP-Corv2 |

*Adjusted for age, sex and education

**Adjusted for age, sex, education and week

***Adjusted for age, sex, education, week and history of covid-19

Table 16 Cox regression for occurrence of symptomatic Covid-19 with positive PCR 14 days after the second injection (participants with Covid-19 history eliminated) in Non-Random arm

| Adjusted HR**  (95.0% CI) | Adjusted HR*  (95.0% CI) | Unadjusted HR  (95.0 % CI) | Study groups |
| --- | --- | --- | --- |
| 1 | 1 | 1 | Fakhravac |
| 0.78  (0.60 – 1.01) | 0.74  (0.57 – 0.96) | 0.69  (0.53 – 0.90) | BBIBP-Corv2 |

*Adjusted for age, sex and education

**Adjusted for age, sex, education and week

### Symptomatic Covid-19 with positive PCR – Total


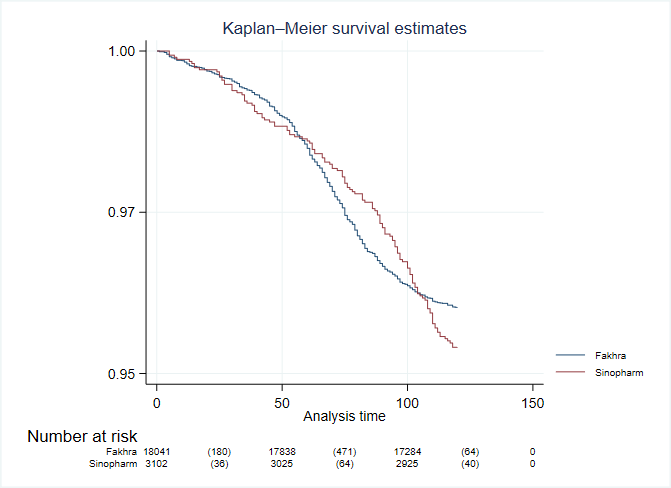


Figure 8 Kaplan-Meier curve for occurrence of symptomatic Covid-19 with positive PCR 14 days after the second injection – Total

Table 17 Logrank test for occurrence of symptomatic Covid-19 with positive PCR 14 days after the second injection – Total

| P-Value log rank | Incidence Rate per 1,000  (95 % CI) | Expected event | Event | person-time | Study groups |
| --- | --- | --- | --- | --- | --- |
| 0.1278 | 0.34  (0.31 – 0.36) | 730.69 | 715 | 2119878 | Fakhravac |
|  | 0.39  (0.33 - 0.46) | 124.31 | 140 | 360705 | BBIBP-Corv2 |

Table 18Cox regression for occurrence of symptomatic Covid-19 with positive PCR 14 days after the second injection - Total

| Adjusted HR****  (95.0% CI) | Adjusted HR***  (95.0% CI) | Adjusted HR**  (95.0% CI) | Adjusted HR*  (95.0% CI) | Unadjusted HR  (95.0 % CI) | Study groups |
| --- | --- | --- | --- | --- | --- |
| 1 | 1 | **1** | 1 | 1 | Fakhravac |
| 0.99  (0.71 – 1.36) | 0.98  (0.70 – 1.35) | **1.11**  **(0.92 – 1.33)** | 1.19  (0.99 – 1.42) | 1.15  (0.96 – 1.37) | BBIBP-Corv2 |

*Adjusted for age, sex and education

**Adjusted for age, sex, education and week

***Adjusted for age, sex, education and week stratified on random/non-random groups

****Adjusted for age, sex, education, week stratified on random/non-random groups and history of covid-19

Table 19Cox regression for occurrence of symptomatic Covid-19 with positive PCR 14 days after the second injection (participants with Covid-19 history eliminated) - Total

| Adjusted HR***  (95.0% CI) | Adjusted HR**  (95.0% CI) | Adjusted HR*  (95.0% CI) | Unadjusted HR  (95.0 % CI) | Study groups |
| --- | --- | --- | --- | --- |
| 1 | 1 | 1 | 1 | Fakhravac |
| 0.97  (0.68 – 1.37) | 1.11  (0.92 – 1.33) | 1.18  (0.98 – 1.42) | 1.15  (0.96 – 1.39) | BBIBP-Corv2 |

*Adjusted for age, sex and education

**Adjusted for age, sex, education and week

***Adjusted for age, sex, education and week stratified on random/non-random groups

### Schematic summary of the survival analysis results of symptomatic Covid-19 with positive PCR

Figure 9 The hazard ratio of the Fakhravac compared to the BBIBP-Corv2 in PCR-positive Covid-19 cases (has been included in the manuscript)


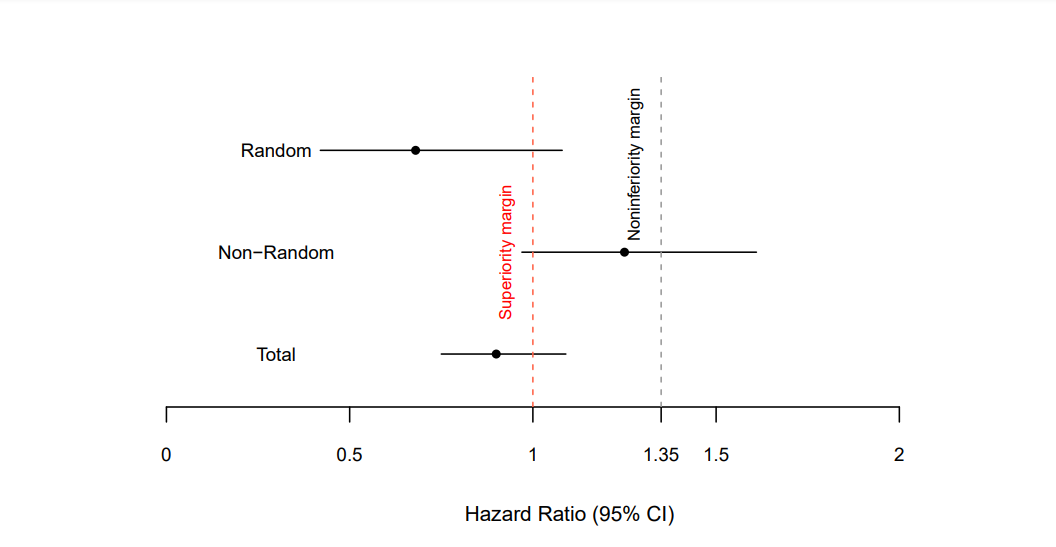


## Symptomatic Covid-19 leading to hospitalization

Table 20 Examining the severity of symptomatic Covid-19 with positive PCR that leading to hospitalization from 14 days after the second injection by study groups

|  | Fakhravac, Random n = 0 | BBIBP-Corv2, Random n = 5 | Fakhravac, Non-Random n = 11 | BBIBP-Corv2, Non-Random n = 3 |
| --- | --- | --- | --- | --- |
| Fever |  | 4 (80%) | 5 (45.45%) | 2 (66.67%) |
| Cough |  | 4 (80%) | 6 (54.55%) | 2 (66.67%) |
| Shortness of breath |  | 4 (80%) | 7 (63.64%) | 2 (66.67%) |
| Fatigue |  | 3 (60%) | 8 (72.73%) | 2 (66.67%) |
| Muscule Pain |  | 1 (20%) | 5 (45.45%) | 1 (33.33%) |
| Headache |  | 4 (80%) | 1 (9.09%) | 2 (66.67%) |
| Anosmia |  | 1 (20%) | 3 (27.27%) | 1 (33.33%) |
| Sore throat/Coryza/Congestion |  | 2 (40%) | 9 (81.82%) | 2 (66.67%) |
| Nausea/Vomiting/Diarrhea |  | 2 (40%) | 3 (27.27%) | 0 (0%) |
| Outpatient refferal |  | 1 (20%) | 6 (54.55%) | 2 (66.67%) |
| ICU admission |  |  | 2 (18.18%) |  |
| Intubation |  |  | 1 (9.09%) |  |
| Multi-organ failure |  |  |  |  |
| Acute kidney failure |  |  |  |  |
| Acute liver failure |  |  | 1 (9.09%) |  |
| Acute left/right ventricular failure |  |  | 1 (9.09%) |  |
| Septic shock |  |  |  |  |
| Ischemic/hemorrhagic stroke |  |  |  |  |
| AMI/DVT/PE* |  |  | 2 (18.18%) |  |
| Mean of minimum O2 Saturation measured by pulse oximeter |  | 95 | 93.27 | 94.67 |
| Possitive CT scan |  |  | 6 (54.55%) |  |
| Lung involved area in CT: n (mean%) |  |  | 6 (23.33%) |  |

### Symptomatic Covid-19 with positive PCR leading to hospitalization in Random arm


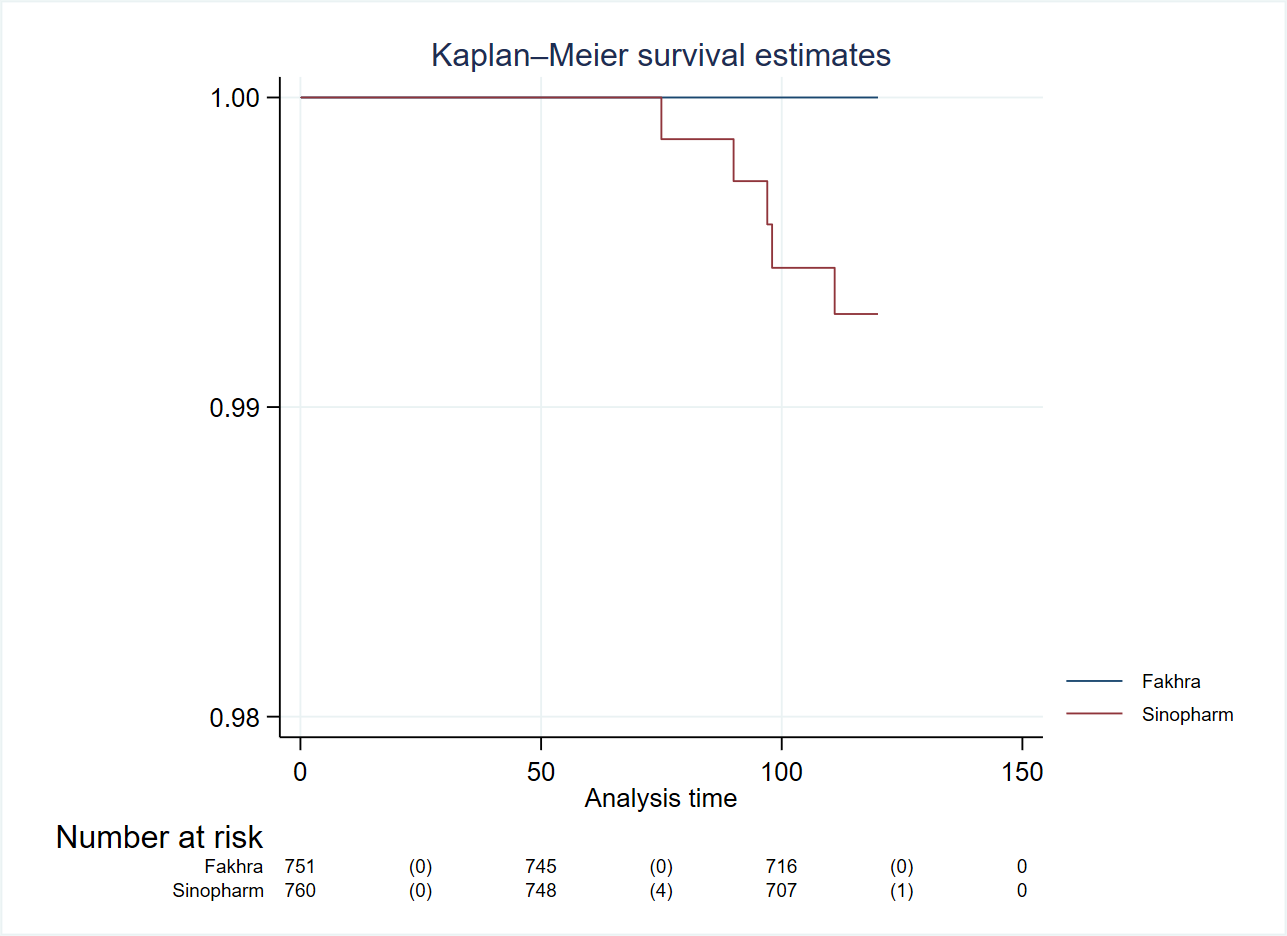


Figure 10 Kaplan-Meier curve for occurrence of symptomatic Covid-19 with positive PCR leading to hospitalization 14 days after the second injection in Random arm

Table 21 Logrank test for occurrence of symptomatic Covid-19 with positive PCR leading to hospitalization 14 days after the second injection in Random arm

| P-Value log rank | Expected event | Event | person-time | Study groups |
| --- | --- | --- | --- | --- |
| 0.0248 | 2.51 | 0 | 88415 | Fakhravac |
|  | 2.49 | 5 | 88453 | BBIBP-Corv2 |


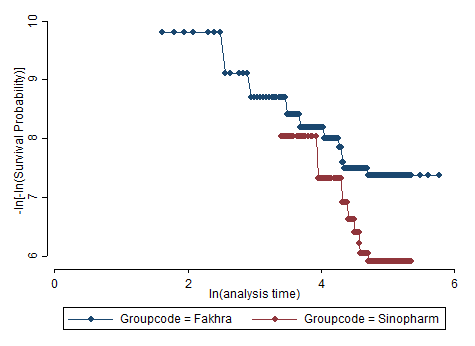


Figure 11 Checking proportional hazard assumption for occurrence of symptomatic Covid-19 with positive PCR leading to hospitalization 14 days after the second injection in Random arm


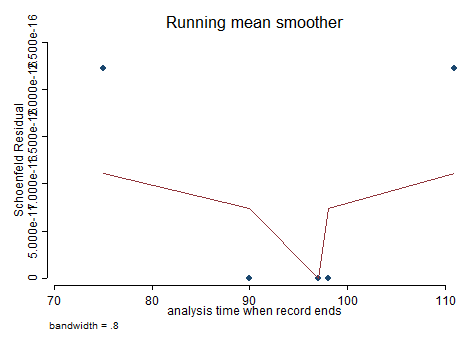


Table 22. Assessment of PH assumption for occurrence of symptomatic Covid-19 with positive PCR leading to hospitalization 14 days after the second injection in Random arm

| P value | df | Chi 2 |  |
| --- | --- | --- | --- |
| 1.00 | 1 | 0.00 | PH assumption |

### Symptomatic Covid-19 with positive PCR leading to hospitalization in Non-Random arm


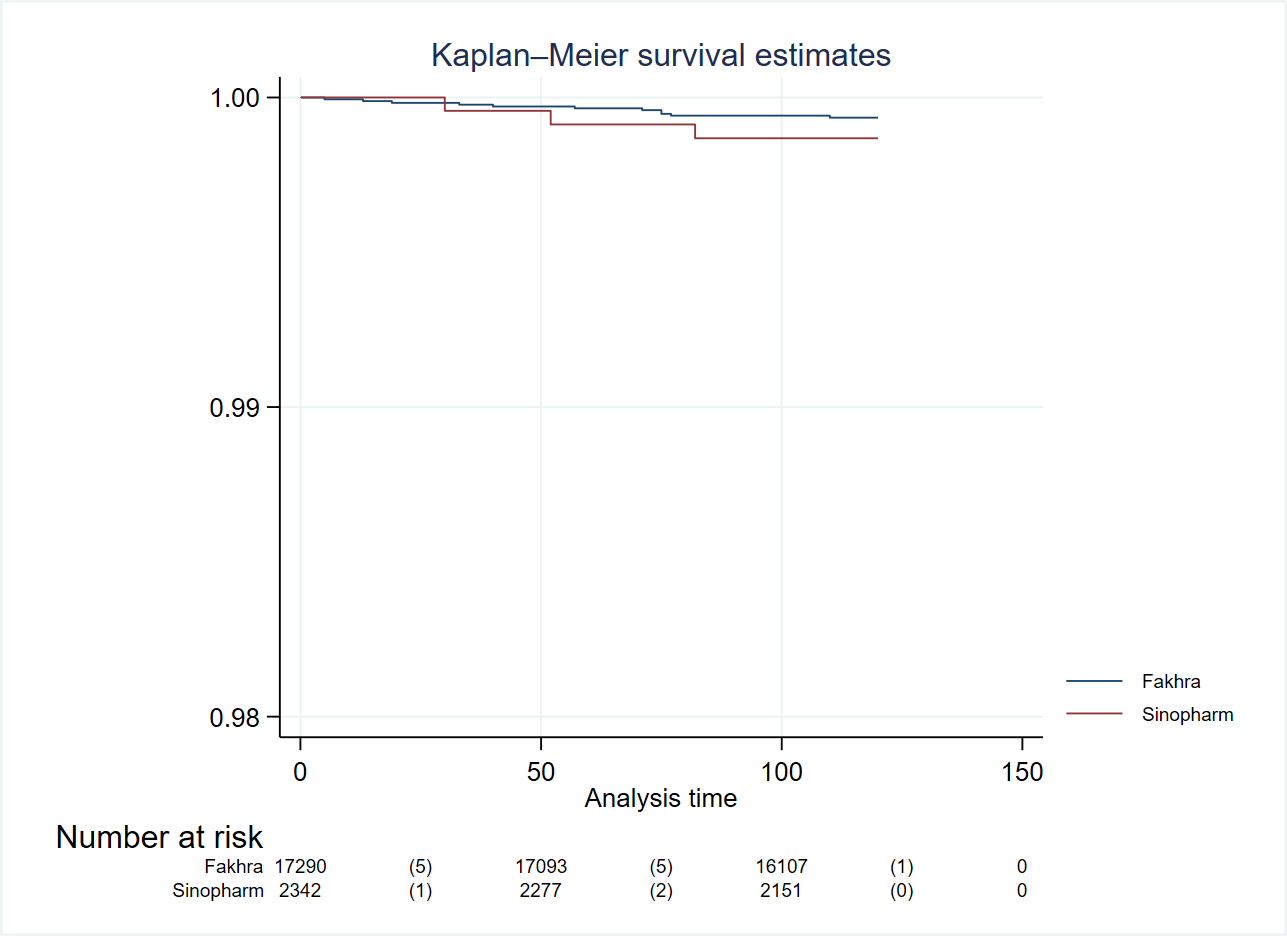


Figure 12 Kaplan-Meier curve for occurrence of symptomatic Covid-19 with positive PCR leading to hospitalization 14 days after the second injection in Non-Random arm

Table 23 Logrank test for occurrence of symptomatic Covid-19 with positive PCR leading to hospitalization 14 days after the second injection in NON-Random arm

| P-Value log rank | Expected event | Event | person-time | Study groups |
| --- | --- | --- | --- | --- |
| 0.2663 | 12.34 | 11 | 2031463 | Fakhravac |
|  | 1.66 | 3 | 272252 | BBIBP-Corv2 |

Table 24 Cox regression for occurrence of symptomatic Covid-19 with positive PCR leading to hospitalization 14 days after the second injection in Non-Random arm

| Adjusted HR***  (95.0% CI) | Adjusted HR**  (95.0% CI) | Adjusted HR*  (95.0% CI) | Unadjusted HR  (95.0 % CI) | Study groups |
| --- | --- | --- | --- | --- |
| 1 | **1** | 1 | 1 | Fakhravac |
| 1.84  (0.40 – 8.50) | **2.02**  **(0.56 – 7.29)** | 2.19  (0.60 – 7.94) | 2.03  (0.57 – 7.28) | BBIBP-Corv2 |

*Adjusted for age, sex and education

**Adjusted for age, sex, education and week

***Adjusted for age, sex, education, week and history of covid-19

Table 25 Cox regression for occurrence of symptomatic Covid-19 with positive PCR leading to hospitalization (participants with Covid-19 history eliminated) 14 days after the second injection in Non-Random arm

| Adjusted HR**  (95.0% CI) | Adjusted HR*  (95.0% CI) | Unadjusted HR  (95.0 % CI) | Study groups |
| --- | --- | --- | --- |
| 1 | 1 | 1 | Fakhravac |
| 1.95  (0.53 – 7.19) | 1.96  (0.54 – 7.13) | 1.88  (0.52 – 6.73) | BBIBP-Corv2 |

*Adjusted for age, sex and education

**Adjusted for age, sex, education and week

# Results: Safety outcomes

## Serious Adverse Event (SAE): Deaths

Table 26 List of participants died during the four month active follow-up

| Vaccine Group | First Injection date | Second Injection date | Death date | Cause of death |
| --- | --- | --- | --- | --- |
| BBIBP-Corv2 | 2021/9/15 | 2021/10/7 | 2021/11/21 | Intra Cranial Hemorrhage |
| Fakhravac | 2021/10/1 | 2021/10/29 | 2021/11/6 | Hypoglycemia |
| Fakhravac | 2021/11/3 |  | 2021/10/2 | Accident |
| Fakhravac | 2021/10/17 | 2021/11/5 | 2021/12/2 | Ischemic heart disease |
| Fakhravac | 2021/10/31 |  | 2021/11/8 | Ischemic heart disease |
| Fakhravac | 2021/12/18 | 2021/12/30 | 2022/3/19 | Ischemic heart disease |
| Fakhravac | 2021/10/31 | 2021/11/24 | 2022/2/19 | Ischemic heart disease |
| BBIBP-Corv2 | 2021/9/18 |  | 2021/9/28 | Ischemic heart disease |

Number of participants receiving BBIBP-Corv2: 3,803

Number of participants reciecing Fakhravac: 20,253

### SMR analysis for death due to heart attack

Table 27 Comparison of observed and expected number of deaths from heart attack in the study arms

| **Group** | **Person Time** | **Observed failures** | **Expected failures** | **SMR** | **[95% conf. interval]** |
| --- | --- | --- | --- | --- | --- |
| **Fakhravac** | | | | | |
| (15 – 20] | 266.76797 | 0 | 0.009011 | 0 |  |
| (20 – 25] | 713.67556 | 0 | 0.03512 | 0 |  |
| (25 – 30] | 795.48802 | 0 | 0.055031 | 0 |  |
| (30 – 35] | 879.51266 | 0 | 0.110119 | 0 |  |
| (35 – 40] | 975.16769 | 1 | 0.209755 | 4.767472 | 0.6715628 - 33.84462 |
| (40 – 45] | 697.48118 | 0 | 0.281504 | 0 |  |
| (45 – 50] | 376.27652 | 1 | 0.292615 | 3.417456 | 0.4813949 - 24.26077 |
| (50 – 55] | 223.6961 | 1 | 0.325676 | 3.070534 | 0.4325262 - 21.79794 |
| (55 – 60] | 142.47228 | 1 | 0.348316 | 2.870958 | 0.4044132 - 20.38114 |
| (60 – 65] | 70.414784 | 0 | 0.26366 | 0 |  |
| (65 – 70] | 32.071184 | 0 | 0.178854 | 0 |  |
| (70 – 75] | 12.845996 | 0 | 0.108544 | 0 |  |
| (75 – 80] | 7.5646817 | 0 | 0.114285 | 0 |  |
| (80 – 85] | 3.5893224 | 0 | 0.086144 | 0 |  |
| (85 – 90] | 2.0862423 | 0 | 0.081208 | 0 |  |
| > 95 | 0.5284052 | 0 | 0.051844 | 0 |  |
| Fakhravac Total | 4325.0568 | 4 | 2.12017 | 1.8866 | 0.70809 - 5.026782 |
| **BBIBP-Corv2** | | | | | |
| (15 – 20] | 44.167009 | 0 | 0.001492 | 0 |  |
| (20 – 25] | 136.47091 | 0 | 0.006716 | 0 |  |
| (25 – 30] | 191.75359 | 0 | 0.013265 | 0 |  |
| (30 – 35] | 152.82957 | 0 | 0.019135 | 0 |  |
| (35 – 40] | 194.48597 | 0 | 0.041833 | 0 |  |
| (40 – 45] | 144.08487 | 0 | 0.058153 | 0 |  |
| (45 – 50] | 79.175907 | 0 | 0.061572 | 0 |  |
| (50 – 55] | 43.167693 | 1 | 0.062847 | 15.91159 | 2.241362 - 112.9575 |
| (55 – 60] | 22.762491 | 0 | 0.05565 | 0 |  |
| (60 – 65] | 15.764545 | 0 | 0.059029 | 0 |  |
| (65 – 70] | 7.4934976 | 0 | 0.04179 | 0 |  |
| (70 – 75] | 2.2614648 | 0 | 0.019109 | 0 |  |
| (75 – 80] | 0.82956879 | 0 | 0.012533 | 0 |  |
| (80 – 85] | 1.5085558 | 0 | 0.036205 | 0 |  |
| BBIBP-Corv2 Total | 861.65366 | 1 | 0.415037 | 2.4094 | 0.3394 - 17.10468 |
| **Overall Total** | **5186.7105** | **5** | **2.53521** | **1.972226** | **0.8208951 – 4.738332** |

## Serious Adverse Event (SAE): Hospitalizations

Table 28 Classification of Serious Adverse Events (SAEs) by cause

| Vaccine Group | Covid-19 Hosp. | Surgery | Internal Medicine Urgency | Cardiovascular  Events | Total |
| --- | --- | --- | --- | --- | --- |
| Fakhravac | 18 (0.737) | 12 (0.441) | 20 (0.736) | 11 (0.405) | 61 (2.25) |
| BBIBP-Corv2 | 4 (0.856) | 2 (0.428) | 5 (1.07) | 1(0.214) | 12 (2.57) |
| Total | 23 (0.722) | 14 (0.439) | 25 (0.785) | 12 (0.377) |  |

* per 100,000

### Serious Adverse Event (SAE) - hospitalizations due to *Covid-19*

Table 29 List of hospitalizations due to Covid-19

| **Row** | **Code** | **First Injection date** | **Second Injection date** | **SAE date** | **PCR result** | **Vaccine type** | **The consequence of the SAE** | **14 days after second injection** |
| --- | --- | --- | --- | --- | --- | --- | --- | --- |
| 1 | 1256 | 2021/9/12 | 2021/10/17 | 2021/12/2 | PCR- | Fakhravac | Discharge | 🗸 |
| 2 | 3700 | 2021/9/13 | 2021/10/4 | 2021/10/12 | PCR+ | Fakhravac | Discharge | - |
| 3 | 7512 | 2021/9/20 |  | 2021/9/22 | PCR+ | Fakhravac | Discharge | - |
| 4 | 7006 | 2021/9/20 |  | 2021/9/22 | PCR+ | Fakhravac | Discharge | - |
| 5 | 8108 | 2021/9/25 |  | 2021/9/28 | PCR+ | Fakhravac | Discharge | - |
| 6 | 9581 | 2021/10/3 | 2021/10/24 | 2021/11/20 | PCR+ | Fakhravac | Discharge | 🗸 |
| 7 | 9629 | 2021/10/9 |  | 2021/10/26 | PCR+ | Fakhravac | Discharge | - |
| 8 | 9349 | 2021/10/14 |  | 2021/10/29 | PCR- | Fakhravac | Discharge | - |
| 9 | 10097 | 2021/10/24 |  | 2021/11/4 | PCR+ | Fakhravac | Discharge | - |
| 10 | 13883 | 2021/10/24 |  | 2021/11/4 | PCR+ | Fakhravac | Discharge | - |
| 11 | 23896 | 2021/10/25 |  | 2021/11/19 | PCR- | Fakhravac | Discharge | - |
| 12 | 23902 | 2021/10/30 |  | 2021/11/18 | PCR+ | Fakhravac | Discharge | - |
| 13 | 26611 | 2021/11/11 | 2021/12/2 | 2021/12/21 | PCR+ | Fakhravac | Discharge | 🗸 |
| 14 | 28179 | 2021/9/18 | 2021/10/9 | 2021/12/22 | PCR+ | Fakhravac | Discharge | 🗸 |
| 15 | 28747 | 2021/9/30 | 2021/11/2 | 2021/11/10 | PCR+ | Fakhravac | Discharge | - |
| 16 | 19114 | 2021/10/23 |  | 2021/12/15 | PCR+ | Fakhravac | Discharge | - |
| 17 | 12466 | 2021/10/13 |  | 2021/11/5 | PCR+ | Fakhravac | Discharge | - |
| 18 | 12934 | 2021/9/29 | 2021/10/21 | 2021/11/22 | PCR- | Fakhravac | Discharge | 🗸 |
| 19 | 13381 | 2021/10/11 | 2021/11/2 | 2022/4/15 | PCR- | Fakhravac | Death | 🗸 |
| 20 | 14893 | 2021/9/15 |  | 2021/9/18 | PCR+ | BBIBP-Corv2 | Discharge | - |
| 21 | 16437 | 2021/9/30 | 2021/10/21 | 2021/10/24 | PCR+ | BBIBP-Corv2 | Discharge | - |
| 22 | 22038 | 2021/10/8 |  | 2021/11/11 | PCR- | BBIBP-Corv2 | Discharge | - |
| 23 | 1299 | 2021/10/9 |  | 2021/11/11 | PCR- | BBIBP-Corv2 | Discharge | - |

### Serious Adverse Event (SAE) - hospitalizations due to surgery

Table 30 List of hospitalizations due to surgery

| **Row** | **Code** | **First Injection date** | **Second Injection date** | **SAE date** | **PCR result** | **Vaccine type** | **The relation to intervention** | **The consequence of the SAE** |
| --- | --- | --- | --- | --- | --- | --- | --- | --- |
| 1 | 16501 | 2021/9/16 | 2021/10/7 | 2022/1/8 | Lumbar disc surgery | Fakhravac | Irrelevant | Discharge |
| 2 | 31063 | 2021/9/30 | 2021/10/21 | 2022/1/1 | LASIK eye surgery | Fakhravac | Irrelevant | Discharge |
| 3 | 31958 | 2021/10/14 | 2021/11/4 | 2021/12/3 | Hysterectomy | Fakhravac | Irrelevant | Discharge |
| 4 | 34168 | 2021/10/16 | 2021/11/11 | 2022/1/8 | Diagnostic curettage | Fakhravac | Irrelevant | Discharge |
| 5 | 22542 | 2021/10/16 | 2021/11/16 | 2021/11/29 | Diagnostic curettage | Fakhravac | Irrelevant | Discharge |
| 6 | 25655 | 2021/10/17 | 2021/11/11 | 2021/11/20 | Inguinal hernia | Fakhravac | Irrelevant | Discharge |
| 7 | 30815 | 2021/10/22 | 2021/12/15 | 2022/1/8 | Rhinoplasty | Fakhravac | Irrelevant | Discharge |
| 8 | 26238 | 2021/11/2 |  | 2021/12/8 | Gastric bypass surgery | Fakhravac | Irrelevant | Discharge |
| 9 | 13576 | 2021/11/14 |  | 2021/12/10 | Hysterectomy | Fakhravac | Irrelevant | Discharge |
| 10 | 25609 | 2021/10/17 | 2021/11/13 | 2021/12/24 | Breast cancer | Fakhravac | Irrelevant | Discharge |
| 11 | 37451 | 2021/11/7 |  | 2022/1/8 | Diagnostic curettage | Fakhravac | Irrelevant | Discharge |
| 12 | 12752 | 2021/10/29 | 2022/1/10 | 2021/12/15 | Cholecystectomy | Fakhravac | Irrelevant | Discharge |
| 13 | 9395 | 2021/9/19 | 2021/10/11 | 2022/1/8 | Arthroscopic knee surgery | BBIBP-Corv2 | Irrelevant | Discharge |
| 14 | 36979 | 2021/10/27 |  | 2021/12/4 | Rhinoplasty | BBIBP-Corv2 | Irrelevant | Discharge |

### Serious Adverse Event (SAE) - hospitalizations due to medical disorders

Table 31 List of hospitalizations due to medical disorders

| **Row** | **Code** | **First Injection date** | **Second Injection date** | **SAE date** | **PCR result** | **Vaccine type** | **The relation to intervention** | **The consequence of the SAE** |
| --- | --- | --- | --- | --- | --- | --- | --- | --- |
| 1 | 36978 | 2021/10/1 | 2021/10/29 | 2021/11/6 | Hypoglycemia | Fakhravac | Irrelevant | Death |
| 2 | 29072 | 2021/10/2 | 2021/10/23 | 2021/11/17 | Hypotension | Fakhravac | Suspicious | Discharge |
| 3 | 14288 | 2021/10/9 |  | 2021/11/21 | Allergic reaction | Fakhravac | Suspicious | Discharge |
| 4 | 23554 | 2021/10/16 | 2021/11/6 | 2021/11/21 | Renal stone | Fakhravac | Irrelevant | Discharge |
| 5 | 18123 | 2021/10/25 | 2021/11/17 | 2021/11/27 | Renal stone | Fakhravac | Irrelevant | Discharge |
| 6 | 27565 | 2021/10/29 | 2021/11/21 |  | Renal stone | Fakhravac | Irrelevant | Discharge |
| 7 | 15886 | 2021/10/29 | 2021/11/21 | 2021/10/27 | Renal stone | Fakhravac | Irrelevant | Discharge |
| 8 | 2187 | 2021/11/6 | 2021/11/27 |  | Vertigo | Fakhravac | Unlikely | Discharge |
| 9 | 9970 | 2021/11/12 |  | 2021/10/25 | Appendicitis | Fakhravac | Irrelevant | Discharge |
| 10 | 17931 | 2021/10/5 | 2021/10/27 | 2021/10/11 | herpes zoster | Fakhravac | Irrelevant | Discharge |
| 11 | 7746 | 2021/10/6 |  |  | Accident | Fakhravac | Irrelevant | Discharge |
| 12 | 36724 | 2021/10/4 | 2021/10/25 | 2021/10/20 | Diabetes | Fakhravac | Irrelevant | Discharge |
| 13 | 1128 | 2021/9/20 | 2021/10/11 |  | Psoriasis | Fakhravac | Suspicious | Discharge |
| 14 | 8427 | 2021/11/19 |  | 2021/11/4 | Endoscopy | Fakhravac | Irrelevant | Discharge |
| 15 | 18396 | 2021/9/29 | 2021/10/20 | 2021/12/21 | Encephalitis | Fakhravac | Irrelevant | Discharge |
| 16 | 12976 | 2021/11/17 |  |  | Headache | Fakhravac | Irrelevant | Discharge |
| 17 | 6544 | 2021/10/14 | 2021/11/4 |  | Allergie | Fakhravac | Irrelevant | Discharge |
| 18 | 7950 | 2021/11/30 | 2021/12/21 |  | Renal stone | Fakhravac | Irrelevant | Discharge |
| 19 | 33311 | 2021/10/9 |  | 2021/10/23 | Middle otitis | Fakhravac | Irrelevant | Discharge |
| 20 | 28200 | 2021/11/3 |  | 2021/10/2 | Accident | Fakhravac | Irrelevant | Death |
| 21 | 11691 | 2021/10/18 |  |  | Axillary lymph adenitis | BBIBP-Corv2 | Irrelevant | Discharge |
| 22 | 13882 | 2021/10/2 | 2021/10/23 | 2021/10/7 | Accident | BBIBP-Corv2 | Irrelevant | Discharge |
| 23 | 27890 | 2021/9/14 | 2021/10/2 | 2021/11/6 | Hypotension | BBIBP-Corv2 | Irrelevant | Discharge |
| 24 | 14879 | 2021/12/15 |  | 2021/11/17 | Psychiatric problem | BBIBP-Corv2 | Irrelevant | Hospitalization |
| 25 | 3678 | 2021/9/15 | 2021/10/7 | 2021/11/21 | Intra Cranial Hemorrhage | BBIBP-Corv2 | Unlikely | Death |

### Serious Adverse Event (SAE) - hospitalizations due to cardiovascular disorders

Table 32 List of hospitalizations due to cardiovascular disorders

| Row | Code | First Injection date | Second Injection date | SAE date | PCR result | Vaccine type | The relation to intervention | The consequence of the SAE |
| --- | --- | --- | --- | --- | --- | --- | --- | --- |
| 1 | 18039 | 2021/10/9 |  | 2022/1/8 | Angiography | Fakhravac | Irrelevant | Discharge |
| 2 | 24696 | 2021/10/10 | 2021/11/3 | 2022/1/8 | Cardiac arrhythmia | Fakhravac | Unlikely | Discharge |
| 3 | 26406 | 2021/10/15 | 2021/11/5 | 2022/1/8 | Angiography | Fakhravac | Irrelevant | Discharge |
| 4 | 22579 | 2021/11/1 | 2021/11/27 | 2021/12/11 | Angiography | Fakhravac | Irrelevant | Discharge |
| 5 | 12878 | 2021/11/6 | 2021/12/2 | 2021/12/6 | Angiography | Fakhravac | Irrelevant | Discharge |
| 6 | 13272 | 2021/10/17 | 2021/11/5 | 2021/12/2 | Ischemic heart disease | Fakhravac | Unlikely | Death |
| 7 | 12876 | 2021/10/30 |  | 2022/1/8 | Ischemic heart disease | Fakhravac | Unlikely | Discharge |
| 8 | 4601 | 2021/10/30 |  | 2022/1/8 | Ischemic heart disease | Fakhravac | Unlikely | Discharge |
| 9 | 10149 | 2021/10/31 |  | 2021/11/8 | Ischemic heart disease | Fakhravac | Unlikely | Death |
| 10 | 19006 | 2021/12/18 | 2021/12/30 | 2022/3/19 | Ischemic heart disease | Fakhravac | Unlikely | Death |
| 11 | 27021 | 2021/10/31 | 2021/11/24 | 2022/2/19 | Ischemic heart disease | Fakhravac | Unlikely | Death |
| 12 | 6575 | 2021/9/18 |  | 2021/9/28 | Ischemic heart disease | BBIBP-Corv2 | Unlikely | Death |

## Solicited local adverse reactions

Table 33 Pain local reaction during first week after first injection

| Characteristic | Overall, N = 24,056 | Fakhravac, Random, N = 824 | BBIBP-Corv2, Random, N = 832 | Fakhravac, Non-Random, N = 19,429 | BBIBP-Corv2, Non-Random, N = 2,971 |
| --- | --- | --- | --- | --- | --- |
| **Day 1** |  |  |  |  |  |
| Normal | 10,247 (71%, N: 14,359) | 463 (72%, N: 639) | 565 (85%, N: 663) | 8,587 (70%, N: 12,345) | 632 (89%, N: 712) |
| Grade 1 | 3,862 (27%, N: 14,359) | 163 (26%, N: 639) | 91 (14%, N: 663) | 3,536 (29%, N: 12,345) | 72 (10%, N: 712) |
| Grade 2 | 190 (1.3%, N: 14,359) | 9 (1.4%, N: 639) | 6 (0.9%, N: 663) | 168 (1.4%, N: 12,345) | 7 (1.0%, N: 712) |
| **Day 2** |  |  |  |  |  |
| Normal | 14,477 (92%, N: 15,760) | 598 (94%, N: 639) | 621 (93%, N: 665) | 12,364 (91%, N: 13,523) | 894 (96%, N: 933) |
| Grade 1 | 1,164 (7.4%, N: 15,760) | 35 (5.5%, N: 639) | 35 (5.3%, N: 665) | 1,063 (7.9%, N: 13,523) | 31 (3.3%, N: 933) |
| Grade 2 | 99 (0.6%, N: 15,760) | 5 (0.8%, N: 639) | 9 (1.4%, N: 665) | 77 (0.6%, N: 13,523) | 8 (0.9%, N: 933) |
| **Day 3** |  |  |  |  |  |
| Normal | 16,664 (97%, N: 17,107) | 617 (97%, N: 635) | 625 (96%, N: 649) | 14,202 (97%, N: 14,580) | 1,220 (98%, N: 1,243) |
| Grade 1 | 357 (2.1%, N: 17,107) | 12 (1.9%, N: 635) | 19 (2.9%, N: 649) | 309 (2.1%, N: 14,580) | 17 (1.4%, N: 1,243) |
| Grade 2 | 69 (0.4%, N: 17,107) | 5 (0.8%, N: 635) | 5 (0.8%, N: 649) | 53 (0.4%, N: 14,580) | 6 (0.5%, N: 1,243) |
| **Day 4** |  |  |  |  |  |
| Normal | 16,524 (98%, N: 16,807) | 557 (98%, N: 567) | 571 (98%, N: 585) | 14,231 (98%, N: 14,473) | 1,165 (99%, N: 1,182) |
| Grade 1 | 204 (1.2%, N: 16,807) | 4 (0.7%, N: 567) | 12 (2.1%, N: 585) | 175 (1.2%, N: 14,473) | 13 (1.1%, N: 1,182) |
| Grade 2 | 58 (0.3%, N: 16,807) | 4 (0.7%, N: 567) | 2 (0.3%, N: 585) | 49 (0.3%, N: 14,473) | 3 (0.3%, N: 1,182) |
| **Day 5** |  |  |  |  |  |
| Normal | 16,713 (99%, N: 16,948) | 533 (98%, N: 542) | 555 (98%, N: 566) | 14,400 (99%, N: 14,602) | 1,225 (99%, N: 1,238) |
| Grade 1 | 164 (1.0%, N: 16,948) | 5 (0.9%, N: 542) | 11 (1.9%, N: 566) | 140 (1.0%, N: 14,602) | 8 (0.6%, N: 1,238) |
| Grade 2 | 53 (0.3%, N: 16,948) | 2 (0.4%, N: 542) | 0 (0%, N: 566) | 47 (0.3%, N: 14,602) | 4 (0.3%, N: 1,238) |
| **Day 6** |  |  |  |  |  |
| Normal | 15,028 (99%, N: 15,198) | 518 (99%, N: 524) | 514 (97%, N: 528) | 12,985 (99%, N: 13,126) | 1,011 (99%, N: 1,020) |
| Grade 1 | 118 (0.8%, N: 15,198) | 4 (0.8%, N: 524) | 8 (1.5%, N: 528) | 101 (0.8%, N: 13,126) | 5 (0.5%, N: 1,020) |
| Grade 2 | 35 (0.2%, N: 15,198) | 2 (0.4%, N: 524) | 6 (1.1%, N: 528) | 25 (0.2%, N: 13,126) | 2 (0.2%, N: 1,020) |

* Grade 3 and 4 cases have been tracked and counted separately in the adverse events section

Table 34 Pain local reaction during first week after second injection

| Characteristic | Overall, N = 24,057 | Fakhravac, Random, N = 824 | BBIBP-Corv2, Random, N = 832 | Fakhravac, Non-Random, N = 19,430 | BBIBP-Corv2, Non-Random, N = 2,971 |
| --- | --- | --- | --- | --- | --- |
| **Day 1** |  |  |  |  |  |
| Normal | 8,153 (76%, N: 10,789) | 316 (72%, N: 440) | 380 (83%, N: 457) | 7,004 (74%, N: 9,407) | 453 (93%, N: 485) |
| Grade 1 | 2,402 (22%, N: 10,789) | 112 (25%, N: 440) | 67 (15%, N: 457) | 2,194 (23%, N: 9,407) | 29 (6.0%, N: 485) |
| Grade 2 | 173 (1.6%, N: 10,789) | 8 (1.8%, N: 440) | 10 (2.2%, N: 457) | 153 (1.6%, N: 9,407) | 2 (0.4%, N: 485) |
| **Day 2** |  |  |  |  |  |
| Normal | 11,682 (92%, N: 12,668) | 425 (91%, N: 469) | 449 (92%, N: 489) | 10,078 (92%, N: 10,967) | 730 (98%, N: 743) |
| Grade 1 | 888 (7.0%, N: 12,668) | 38 (8.1%, N: 469) | 35 (7.2%, N: 489) | 806 (7.3%, N: 10,967) | 9 (1.2%, N: 743) |
| Grade 2 | 77 (0.6%, N: 12,668) | 5 (1.1%, N: 469) | 5 (1.0%, N: 489) | 63 (0.6%, N: 10,967) | 4 (0.5%, N: 743) |
| **Day 3** |  |  |  |  |  |
| Normal | 13,988 (98%, N: 14,318) | 478 (97%, N: 494) | 480 (97%, N: 495) | 12,040 (98%, N: 12,332) | 990 (99%, N: 997) |
| Grade 1 | 283 (2.0%, N: 14,318) | 13 (2.6%, N: 494) | 13 (2.6%, N: 495) | 251 (2.0%, N: 12,332) | 6 (0.6%, N: 997) |
| Grade 2 | 36 (0.3%, N: 14,318) | 2 (0.4%, N: 494) | 2 (0.4%, N: 495) | 31 (0.3%, N: 12,332) | 1 (0.1%, N: 997) |
| **Day 4** |  |  |  |  |  |
| Normal | 14,122 (99%, N: 14,335) | 472 (97%, N: 485) | 471 (98%, N: 481) | 12,163 (99%, N: 12,344) | 1,016 (99%, N: 1,025) |
| Grade 1 | 173 (1.2%, N: 14,335) | 11 (2.3%, N: 485) | 6 (1.2%, N: 481) | 149 (1.2%, N: 12,344) | 7 (0.7%, N: 1,025) |
| Grade 2 | 30 (0.2%, N: 14,335) | 2 (0.4%, N: 485) | 2 (0.4%, N: 481) | 24 (0.2%, N: 12,344) | 2 (0.2%, N: 1,025) |
| **Day 5** |  |  |  |  |  |
| Normal | 14,738 (99%, N: 14,885) | 483 (98%, N: 491) | 493 (99%, N: 500) | 12,606 (99%, N: 12,728) | 1,156 (99%, N: 1,166) |
| Grade 1 | 113 (0.8%, N: 14,885) | 6 (1.2%, N: 491) | 4 (0.8%, N: 500) | 95 (0.7%, N: 12,728) | 8 (0.7%, N: 1,166) |
| Grade 2 | 27 (0.2%, N: 14,885) | 1 (0.2%, N: 491) | 3 (0.6%, N: 500) | 22 (0.2%, N: 12,728) | 1 (<0.1%, N: 1,166) |
| **Day 6** |  |  |  |  |  |
| Normal | 12,884 (99%, N: 13,004) | 453 (99%, N: 458) | 484 (99%, N: 489) | 10,971 (99%, N: 11,073) | 976 (99%, N: 984) |
| Grade 1 | 93 (0.7%, N: 13,004) | 3 (0.7%, N: 458) | 4 (0.8%, N: 489) | 81 (0.7%, N: 11,073) | 5 (0.5%, N: 984) |
| Grade 2 | 21 (0.2%, N: 13,004) | 2 (0.4%, N: 458) | 0 (0%, N: 489) | 16 (0.1%, N: 11,073) | 3 (0.3%, N: 984) |

* Grade 3 and 4 cases have been tracked and counted separately in the adverse events section

Table 35 Tenderness local reaction during first week after first injection

| Characteristic | Overall, N = 24,056 | Fakhravac, Random, N = 824 | BBIBP-Corv2, Random, N = 832 | Fakhravac, Non-Random, N = 19,429 | BBIBP-Corv2, Non-Random, N = 2,971 |
| --- | --- | --- | --- | --- | --- |
| **Day 1** |  |  |  |  |  |
| Normal | 7,611 (52%, N: 14,521) | 329 (51%, N: 646) | 513 (77%, N: 669) | 6,164 (49%, N: 12,485) | 605 (84%, N: 721) |
| Grade 1 | 6,910 (48%, N: 14,521) | 317 (49%, N: 646) | 156 (23%, N: 669) | 6,321 (51%, N: 12,485) | 116 (16%, N: 721) |
| **Day 2** |  |  |  |  |  |
| Normal | 13,035 (83%, N: 15,798) | 515 (80%, N: 642) | 598 (90%, N: 666) | 11,036 (81%, N: 13,557) | 886 (95%, N: 933) |
| Grade 1 | 2,763 (17%, N: 15,798) | 127 (20%, N: 642) | 68 (10%, N: 666) | 2,521 (19%, N: 13,557) | 47 (5.0%, N: 933) |
| **Day 3** |  |  |  |  |  |
| Normal | 16,449 (96%, N: 17,123) | 607 (96%, N: 635) | 631 (97%, N: 650) | 13,992 (96%, N: 14,595) | 1,219 (98%, N: 1,243) |
| Grade 1 | 674 (3.9%, N: 17,123) | 28 (4.4%, N: 635) | 19 (2.9%, N: 650) | 603 (4.1%, N: 14,595) | 24 (1.9%, N: 1,243) |
| **Day 4** |  |  |  |  |  |
| Normal | 16,552 (98%, N: 16,815) | 555 (98%, N: 567) | 575 (98%, N: 585) | 14,247 (98%, N: 14,481) | 1,175 (99%, N: 1,182) |
| Grade 1 | 263 (1.6%, N: 16,815) | 12 (2.1%, N: 567) | 10 (1.7%, N: 585) | 234 (1.6%, N: 14,481) | 7 (0.6%, N: 1,182) |
| **Day 5** |  |  |  |  |  |
| Normal | 16,776 (99%, N: 16,956) | 532 (98%, N: 543) | 559 (99%, N: 566) | 14,449 (99%, N: 14,608) | 1,236 (100%, N: 1,239) |
| Grade 1 | 180 (1.1%, N: 16,956) | 11 (2.0%, N: 543) | 7 (1.2%, N: 566) | 159 (1.1%, N: 14,608) | 3 (0.2%, N: 1,239) |
|  |  |  |  |  |  |
| **Day 6** |  |  |  |  |  |
| Normal | 15,092 (99%, N: 15,206) | 517 (99%, N: 524) | 523 (99%, N: 528) | 13,036 (99%, N: 13,134) | 1,016 (100%, N: 1,020) |
| Grade 1 | 114 (0.7%, N: 15,206) | 7 (1.3%, N: 524) | 5 (0.9%, N: 528) | 98 (0.7%, N: 13,134) | 4 (0.4%, N: 1,020) |

* Grade 3 and 4 cases have been tracked and counted separately in the adverse events section

Table 36 Tenderness local reaction during first week after second injection

| Characteristic | Overall, N = 24,056 | Fakhravac, Random, N = 824 | BBIBP-Corv2, Random, N = 832 | Fakhravac, Non-Random, N = 19,429 | BBIBP-Corv2, Non-Random, N = 2,971 |
| --- | --- | --- | --- | --- | --- |
| **Day 1** |  |  |  |  |  |
| Normal | 8,153 (76%, N: 10,789) | 316 (72%, N: 440) | 380 (83%, N: 457) | 7,004 (74%, N: 9,407) | 453 (93%, N: 485) |
| Grade 1 | 2,402 (22%, N: 10,789) | 112 (25%, N: 440) | 67 (15%, N: 457) | 2,194 (23%, N: 9,407) | 29 (6.0%, N: 485) |
| **Day 2** |  |  |  |  |  |
| Normal | 11,682 (92%, N: 12,668) | 425 (91%, N: 469) | 449 (92%, N: 489) | 10,078 (92%, N: 10,967) | 730 (98%, N: 743) |
| Grade 1 | 888 (7.0%, N: 12,668) | 38 (8.1%, N: 469) | 35 (7.2%, N: 489) | 806 (7.3%, N: 10,967) | 9 (1.2%, N: 743) |
| **Day 3** |  |  |  |  |  |
| Normal | 13,988 (98%, N: 14,318) | 478 (97%, N: 494) | 480 (97%, N: 495) | 12,040 (98%, N: 12,332) | 990 (99%, N: 997) |
| Grade 1 | 283 (2.0%, N: 14,318) | 13 (2.6%, N: 494) | 13 (2.6%, N: 495) | 251 (2.0%, N: 12,332) | 6 (0.6%, N: 997) |
| **Day 4** |  |  |  |  |  |
| Normal | 14,122 (99%, N: 14,335) | 472 (97%, N: 485) | 471 (98%, N: 481) | 12,163 (99%, N: 12,344) | 1,016 (99%, N: 1,025) |
| Grade 1 | 173 (1.2%, N: 14,335) | 11 (2.3%, N: 485) | 6 (1.2%, N: 481) | 149 (1.2%, N: 12,344) | 7 (0.7%, N: 1,025) |
| **Day 5** |  |  |  |  |  |
| Normal | 14,738 (99%, N: 14,885) | 483 (98%, N: 491) | 493 (99%, N: 500) | 12,606 (99%, N: 12,728) | 1,156 (99%, N: 1,166) |
| Grade 1 | 113 (0.8%, N: 14,885) | 6 (1.2%, N: 491) | 4 (0.8%, N: 500) | 95 (0.7%, N: 12,728) | 8 (0.7%, N: 1,166) |
| **Day 6** |  |  |  |  |  |
| Normal | 12,884 (99%, N: 13,004) | 453 (99%, N: 458) | 484 (99%, N: 489) | 10,971 (99%, N: 11,073) | 976 (99%, N: 984) |
| Grade 1 | 93 (0.7%, N: 13,004) | 3 (0.7%, N: 458) | 4 (0.8%, N: 489) | 81 (0.7%, N: 11,073) | 5 (0.5%, N: 984) |

* Grade 3 and 4 cases have been tracked and counted separately in the adverse events section

Table 37 Redness local reaction during first week after first injection

| Characteristic | Overall, N = 24,056 | Fakhravac, Random, N = 824 | BBIBP-Corv2, Random, N = 832 | Fakhravac, Non-Random, N = 19,429 | BBIBP-Corv2, Non-Random, N = 2,971 |
| --- | --- | --- | --- | --- | --- |
| **Day 1** |  |  |  |  |  |
| Normal | 14,350 (99%, N: 14,521) | 638 (99%, N: 646) | 662 (99%, N: 669) | 12,335 (99%, N: 12,485) | 715 (99%, N: 721) |
| Grade 1 | 171 (1.2%, N: 14,521) | 8 (1.2%, N: 646) | 7 (1.0%, N: 669) | 150 (1.2%, N: 12,485) | 6 (0.8%, N: 721) |
| **Day 2** |  |  |  |  |  |
| Normal | 15,701 (99%, N: 15,798) | 632 (98%, N: 642) | 659 (99%, N: 666) | 13,481 (99%, N: 13,557) | 929 (100%, N: 933) |
| Grade 1 | 97 (0.6%, N: 15,798) | 10 (1.6%, N: 642) | 7 (1.1%, N: 666) | 76 (0.6%, N: 13,557) | 4 (0.4%, N: 933) |
| **Day 3** |  |  |  |  |  |
| Normal | 17,069 (100%, N: 17,123) | 633 (100%, N: 635) | 648 (100%, N: 650) | 14,550 (100%, N: 14,595) | 1,238 (100%, N: 1,243) |
| Grade 1 | 54 (0.3%, N: 17,123) | 2 (0.3%, N: 635) | 2 (0.3%, N: 650) | 45 (0.3%, N: 14,595) | 5 (0.4%, N: 1,243) |
| **Day 4** |  |  |  |  |  |
| Normal | 16,778 (100%, N: 16,815) | 565 (100%, N: 567) | 584 (100%, N: 585) | 14,451 (100%, N: 14,481) | 1,178 (100%, N: 1,182) |
| Grade 1 | 37 (0.2%, N: 16,815) | 2 (0.4%, N: 567) | 1 (0.2%, N: 585) | 30 (0.2%, N: 14,481) | 4 (0.3%, N: 1,182) |
| **Day 5** |  |  |  |  |  |
| Normal | 16,930 (100%, N: 16,956) | 541 (100%, N: 543) | 564 (100%, N: 566) | 14,588 (100%, N: 14,608) | 1,237 (100%, N: 1,239) |
| Grade 1 | 26 (0.2%, N: 16,956) | 2 (0.4%, N: 543) | 2 (0.4%, N: 566) | 20 (0.1%, N: 14,608) | 2 (0.2%, N: 1,239) |
| **Day 6** |  |  |  |  |  |
| Normal | 15,183 (100%, N: 15,206) | 522 (100%, N: 524) | 526 (100%, N: 528) | 13,116 (100%, N: 13,134) | 1,019 (100%, N: 1,020) |
| Grade 1 | 23 (0.2%, N: 15,206) | 2 (0.4%, N: 524) | 2 (0.4%, N: 528) | 18 (0.1%, N: 13,134) | 1 (<0.1%, N: 1,020) |

* Grade 3 and 4 cases have been tracked and counted separately in the adverse events section

Table 38 Redness local reaction during first week after second injection

| Characteristic | Overall, N = 24,056 | Fakhravac, Random, N = 824 | BBIBP-Corv2, Random, N = 832 | Fakhravac, Non-Random, N = 19,429 | BBIBP-Corv2, Non-Random, N = 2,971 |
| --- | --- | --- | --- | --- | --- |
| **Day 1** |  |  |  |  |  |
| Normal | 10,741 (99%, N: 10,876) | 441 (99%, N: 444) | 458 (100%, N: 460) | 9,357 (99%, N: 9,486) | 485 (100%, N: 486) |
| Grade 1 | 135 (1.2%, N: 10,876) | 3 (0.7%, N: 444) | 2 (0.4%, N: 460) | 129 (1.4%, N: 9,486) | 1 (0.2%, N: 486) |
| **Day 2** |  |  |  |  |  |
| Normal | 12,647 (100%, N: 12,694) | 470 (100%, N: 471) | 489 (100%, N: 489) | 10,945 (100%, N: 10,990) | 743 (100%, N: 744) |
| Grade 1 | 47 (0.4%, N: 12,694) | 1 (0.2%, N: 471) | 0 (0%, N: 489) | 45 (0.4%, N: 10,990) | 1 (0.1%, N: 744) |
| **Day 3** |  |  |  |  |  |
| Normal | 14,299 (100%, N: 14,329) | 494 (100%, N: 494) | 494 (100%, N: 496) | 12,316 (100%, N: 12,342) | 995 (100%, N: 997) |
| Grade 1 | 30 (0.2%, N: 14,329) | 0 (0%, N: 494) | 2 (0.4%, N: 496) | 26 (0.2%, N: 12,342) | 2 (0.2%, N: 997) |
| **Day 4** |  |  |  |  |  |
| Normal | 14,324 (100%, N: 14,340) | 485 (100%, N: 485) | 480 (100%, N: 481) | 12,333 (100%, N: 12,348) | 1,026 (100%, N: 1,026) |
| Grade 1 | 16 (0.1%, N: 14,340) | 0 (0%, N: 485) | 1 (0.2%, N: 481) | 15 (0.1%, N: 12,348) | 0 (0%, N: 1,026) |
| **Day 5** |  |  |  |  |  |
| Normal | 14,881 (100%, N: 14,893) | 491 (100%, N: 491) | 500 (100%, N: 500) | 12,723 (100%, N: 12,735) | 1,167 (100%, N: 1,167) |
| Grade 1 | 12 (<0.1%, N: 14,893) | 0 (0%, N: 491) | 0 (0%, N: 500) | 12 (<0.1%, N: 12,735) | 0 (0%, N: 1,167) |
| **Day 6** |  |  |  |  |  |
| Normal | 12,996 (100%, N: 13,006) | 458 (100%, N: 458) | 489 (100%, N: 489) | 11,065 (100%, N: 11,075) | 984 (100%, N: 984) |
| Grade 1 | 10 (<0.1%, N: 13,006) | 0 (0%, N: 458) | 0 (0%, N: 489) | 10 (<0.1%, N: 11,075) | 1. (0%, N: 984) |

* Grade 3 and 4 cases have been tracked and counted separately in the adverse events section

Table 39 Induration local reaction during first week after first injection

| Characteristic | Overall, N = 24,056 | Fakhravac, Random, N = 824 | BBIBP-Corv2, Random, N = 832 | Fakhravac, Non-Random, N = 19,429 | BBIBP-Corv2, Non-Random, N = 2,971 |
| --- | --- | --- | --- | --- | --- |
| **Day 1** |  |  |  |  |  |
| Normal | 13,427 (92%, N: 14,521) | 598 (93%, N: 646) | 644 (96%, N: 669) | 11,480 (92%, N: 12,485) | 705 (98%, N: 721) |
| Grade 1 | 1,094 (7.5%, N: 14,521) | 48 (7.4%, N: 646) | 25 (3.7%, N: 669) | 1,005 (8.0%, N: 12,485) | 16 (2.2%, N: 721) |
| **Day 2** |  |  |  |  |  |
| Normal | 15,432 (98%, N: 15,798) | 624 (97%, N: 642) | 657 (99%, N: 666) | 13,226 (98%, N: 13,557) | 925 (99%, N: 933) |
| Grade 1 | 366 (2.3%, N: 15,798) | 18 (2.8%, N: 642) | 9 (1.4%, N: 666) | 331 (2.4%, N: 13,557) | 8 (0.9%, N: 933) |
| **Day 3** |  |  |  |  |  |
| Normal | 17,010 (99%, N: 17,123) | 633 (100%, N: 635) | 646 (99%, N: 650) | 14,494 (99%, N: 14,595) | 1,237 (100%, N: 1,243) |
| Grade 1 | 113 (0.7%, N: 17,123) | 2 (0.3%, N: 635) | 4 (0.6%, N: 650) | 101 (0.7%, N: 14,595) | 6 (0.5%, N: 1,243) |
| **Day 4** |  |  |  |  |  |
| Normal | 16,747 (100%, N: 16,815) | 562 (99%, N: 567) | 583 (100%, N: 585) | 14,422 (100%, N: 14,481) | 1,180 (100%, N: 1,182) |
| Grade 1 | 68 (0.4%, N: 16,815) | 5 (0.9%, N: 567) | 2 (0.3%, N: 585) | 59 (0.4%, N: 14,481) | 2 (0.2%, N: 1,182) |
| **Day 5** |  |  |  |  |  |
| Normal | 16,925 (100%, N: 16,956) | 542 (100%, N: 543) | 565 (100%, N: 566) | 14,579 (100%, N: 14,608) | 1,239 (100%, N: 1,239) |
| Grade 1 | 31 (0.2%, N: 16,956) | 1 (0.2%, N: 543) | 1 (0.2%, N: 566) | 29 (0.2%, N: 14,608) | 0 (0%, N: 1,239) |
| **Day 6** |  |  |  |  |  |
| Normal | 15,177 (100%, N: 15,206) | 523 (100%, N: 524) | 527 (100%, N: 528) | 13,107 (100%, N: 13,134) | 1,020 (100%, N: 1,020) |
| Grade 1 | 29 (0.2%, N: 15,206) | 1 (0.2%, N: 524) | 1 (0.2%, N: 528) | 27 (0.2%, N: 13,134) | 0 (0%, N: 1,020) |

* Grade 3 and 4 cases have been tracked and counted separately in the adverse events section

Table 40 Induration local reaction during first week after second injection

| Characteristic | Overall, N = 24,056 | Fakhravac, Random, N = 824 | BBIBP-Corv2, Random, N = 832 | Fakhravac, Non-Random, N = 19,429 | BBIBP-Corv2, Non-Random, N = 2,971 |
| --- | --- | --- | --- | --- | --- |
| **Day 1** |  |  |  |  |  |
| Normal | 10,219 (94%, N: 10,876) | 412 (93%, N: 444) | 453 (98%, N: 460) | 8,872 (94%, N: 9,486) | 482 (99%, N: 486) |
| Grade 1 | 657 (6.0%, N: 10,876) | 32 (7.2%, N: 444) | 7 (1.5%, N: 460) | 614 (6.5%, N: 9,486) | 4 (0.8%, N: 486) |
| **Day 2** |  |  |  |  |  |
| Normal | 12,465 (98%, N: 12,694) | 461 (98%, N: 471) | 485 (99%, N: 489) | 10,776 (98%, N: 10,990) | 743 (100%, N: 744) |
| Grade 1 | 229 (1.8%, N: 12,694) | 10 (2.1%, N: 471) | 4 (0.8%, N: 489) | 214 (1.9%, N: 10,990) | 1 (0.1%, N: 744) |
| **Day 3** |  |  |  |  |  |
| Normal | 14,259 (100%, N: 14,329) | 493 (100%, N: 494) | 493 (99%, N: 496) | 12,278 (99%, N: 12,342) | 995 (100%, N: 997) |
| Grade 1 | 70 (0.5%, N: 14,329) | 1 (0.2%, N: 494) | 3 (0.6%, N: 496) | 64 (0.5%, N: 12,342) | 2 (0.2%, N: 997) |
| **Day 4** |  |  |  |  |  |
| Normal | 14,298 (100%, N: 14,340) | 484 (100%, N: 485) | 481 (100%, N: 481) | 12,307 (100%, N: 12,348) | 1,026 (100%, N: 1,026) |
| Grade 1 | 42 (0.3%, N: 14,340) | 1 (0.2%, N: 485) | 0 (0%, N: 481) | 41 (0.3%, N: 12,348) | 0 (0%, N: 1,026) |
| **Day 5** |  |  |  |  |  |
| Normal | 14,866 (100%, N: 14,893) | 490 (100%, N: 491) | 500 (100%, N: 500) | 12,709 (100%, N: 12,735) | 1,167 (100%, N: 1,167) |
| Grade 1 | 27 (0.2%, N: 14,893) | 1 (0.2%, N: 491) | 0 (0%, N: 500) | 26 (0.2%, N: 12,735) | 0 (0%, N: 1,167) |
| **Day 6** |  |  |  |  |  |
| Normal | 12,987 (100%, N: 13,006) | 458 (100%, N: 458) | 488 (100%, N: 489) | 11,057 (100%, N: 11,075) | 984 (100%, N: 984) |
| Grade 1 | 19 (0.1%, N: 13,006) | 0 (0%, N: 458) | 1 (0.2%, N: 489) | 18 (0.2%, N: 11,075) | 0 (0%, N: 984) |

* Grade 3 and 4 cases have been tracked and counted separately in the adverse events section

## Solicited systemic adverse reactions

Table 41 Vomiting systemic reaction during first week after first injection

| Characteristic | Overall, N = 24,056 | Fakhravac, Random, N = 824 | BBIBP-Corv2, Random, N = 832 | Fakhravac, Non-Random, N = 19,429 | BBIBP-Corv2, Non-Random, N = 2,971 |
| --- | --- | --- | --- | --- | --- |
| **Day 1** |  |  |  |  |  |
| Normal | 11,007 (99%, N: 11,136) | 418 (100%, N: 420) | 436 (99%, N: 440) | 9,593 (99%, N: 9,708) | 560 (99%, N: 568) |
| Grade 1 | 104 (0.9%, N: 11,136) | 1 (0.2%, N: 420) | 4 (0.9%, N: 440) | 93 (1.0%, N: 9,708) | 6 (1.1%, N: 568) |
| Grade 2 | 23 (0.2%, N: 11,136) | 1 (0.2%, N: 420) | 0 (0%, N: 440) | 20 (0.2%, N: 9,708) | 2 (0.4%, N: 568) |
| **Day 2** |  |  |  |  |  |
| Normal | 13,249 (99%, N: 13,358) | 413 (100%, N: 415) | 447 (100%, N: 449) | 11,567 (99%, N: 11,660) | 822 (99%, N: 834) |
| Grade 1 | 89 (0.7%, N: 13,358) | 2 (0.5%, N: 415) | 2 (0.4%, N: 449) | 76 (0.7%, N: 11,660) | 9 (1.1%, N: 834) |
| Grade 2 | 15 (0.1%, N: 13,358) | 0 (0%, N: 415) | 0 (0%, N: 449) | 13 (0.1%, N: 11,660) | 2 (0.2%, N: 834) |
| **Day 3** |  |  |  |  |  |
| Normal | 15,257 (99%, N: 15,342) | 431 (99%, N: 436) | 461 (99%, N: 464) | 13,203 (99%, N: 13,272) | 1,162 (99%, N: 1,170) |
| Grade 1 | 66 (0.4%, N: 15,342) | 4 (0.9%, N: 436) | 3 (0.6%, N: 464) | 52 (0.4%, N: 13,272) | 7 (0.6%, N: 1,170) |
| Grade 2 | 15 (<0.1%, N: 15,342) | 0 (0%, N: 436) | 0 (0%, N: 464) | 14 (0.1%, N: 13,272) | 1 (<0.1%, N: 1,170) |
| **Day 4** |  |  |  |  |  |
| Normal | 15,084 (99%, N: 15,168) | 371 (99%, N: 375) | 389 (99%, N: 393) | 13,208 (99%, N: 13,278) | 1,116 (99%, N: 1,122) |
| Grade 1 | 65 (0.4%, N: 15,168) | 1 (0.3%, N: 375) | 4 (1.0%, N: 393) | 55 (0.4%, N: 13,278) | 5 (0.4%, N: 1,122) |
| Grade 2 | 13 (<0.1%, N: 15,168) | 3 (0.8%, N: 375) | 0 (0%, N: 393) | 9 (<0.1%, N: 13,278) | 1 (<0.1%, N: 1,122) |
| **Day 5** |  |  |  |  |  |
| Normal | 15,460 (100%, N: 15,536) | 362 (100%, N: 363) | 379 (99%, N: 381) | 13,553 (100%, N: 13,621) | 1,166 (100%, N: 1,171) |
| Grade 1 | 58 (0.4%, N: 15,536) | 1 (0.3%, N: 363) | 2 (0.5%, N: 381) | 50 (0.4%, N: 13,621) | 5 (0.4%, N: 1,171) |
| Grade 2 | 16 (0.1%, N: 15,536) | 0 (0%, N: 363) | 0 (0%, N: 381) | 16 (0.1%, N: 13,621) | 0 (0%, N: 1,171) |
| **Day 6** |  |  |  |  |  |
| Normal | 13,571 (100%, N: 13,631) | 350 (99%, N: 352) | 366 (99%, N: 368) | 11,906 (100%, N: 11,958) | 949 (100%, N: 953) |
| Grade 1 | 41 (0.3%, N: 13,631) | 1 (0.3%, N: 352) | 1 (0.3%, N: 368) | 37 (0.3%, N: 11,958) | 2 (0.2%, N: 953) |
| Grade 2 | 13 (<0.1%, N: 13,631) | 1 (0.3%, N: 352) | 1 (0.3%, N: 368) | 9 (<0.1%, N: 11,958) | 2 (0.2%, N: 953) |

* Grade 3 and 4 cases have been tracked and counted separately in the adverse events section

Table 42 Vomiting systemic reaction during first week after second injection

| Characteristic | Overall, N = 24,056 | Fakhravac, Random, N = 824 | BBIBP-Corv2, Random, N = 832 | Fakhravac, Non-Random, N = 19,429 | BBIBP-Corv2, Non-Random, N = 2,971 |
| --- | --- | --- | --- | --- | --- |
| **Day 1** |  |  |  |  |  |
| Normal | 9,356 (99%, N: 9,461) | 353 (99%, N: 355) | 393 (99%, N: 395) | 8,159 (99%, N: 8,257) | 451 (99%, N: 454) |
| Grade 1 | 89 (0.9%, N: 9,461) | 2 (0.6%, N: 355) | 2 (0.5%, N: 395) | 83 (1.0%, N: 8,257) | 2 (0.4%, N: 454) |
| Grade 2 | 12 (0.1%, N: 9,461) | 0 (0%, N: 355) | 0 (0%, N: 395) | 12 (0.1%, N: 8,257) | 0 (0%, N: 454) |
| **Day 2** |  |  |  |  |  |
| Normal | 11,629 (99%, N: 11,712) | 399 (100%, N: 401) | 425 (100%, N: 427) | 10,080 (99%, N: 10,158) | 725 (100%, N: 726) |
| Grade 1 | 68 (0.6%, N: 11,712) | 1 (0.2%, N: 401) | 2 (0.5%, N: 427) | 64 (0.6%, N: 10,158) | 1 (0.1%, N: 726) |
| Grade 2 | 10 (<0.1%, N: 11,712) | 1 (0.2%, N: 401) | 0 (0%, N: 427) | 9 (<0.1%, N: 10,158) | 0 (0%, N: 726) |
| **Day 3** |  |  |  |  |  |
| Normal | 13,629 (100%, N: 13,697) | 432 (99%, N: 435) | 451 (99%, N: 456) | 11,776 (100%, N: 11,831) | 970 (99%, N: 975) |
| Grade 1 | 54 (0.4%, N: 13,697) | 2 (0.5%, N: 435) | 4 (0.9%, N: 456) | 46 (0.4%, N: 11,831) | 2 (0.2%, N: 975) |
| Grade 2 | 12 (<0.1%, N: 13,697) | 1 (0.2%, N: 435) | 1 (0.2%, N: 456) | 8 (<0.1%, N: 11,831) | 2 (0.2%, N: 975) |
| **Day 4** |  |  |  |  |  |
| Normal | 13,708 (100%, N: 13,759) | 427 (99%, N: 430) | 428 (100%, N: 429) | 11,839 (100%, N: 11,884) | 1,014 (100%, N: 1,016) |
| Grade 1 | 35 (0.3%, N: 13,759) | 1 (0.2%, N: 430) | 1 (0.2%, N: 429) | 32 (0.3%, N: 11,884) | 1 (<0.1%, N: 1,016) |
| Grade 2 | 13 (<0.1%, N: 13,759) | 2 (0.5%, N: 430) | 0 (0%, N: 429) | 11 (<0.1%, N: 11,884) | 0 (0%, N: 1,016) |
| **Day 5** |  |  |  |  |  |
| Normal | 14,347 (100%, N: 14,404) | 442 (99%, N: 446) | 441 (100%, N: 443) | 12,317 (100%, N: 12,366) | 1,147 (100%, N: 1,149) |
| Grade 1 | 41 (0.3%, N: 14,404) | 2 (0.4%, N: 446) | 2 (0.5%, N: 443) | 36 (0.3%, N: 12,366) | 1 (<0.1%, N: 1,149) |
| Grade 2 | 13 (<0.1%, N: 14,404) | 2 (0.4%, N: 446) | 0 (0%, N: 443) | 11 (<0.1%, N: 12,366) | 0 (0%, N: 1,149) |
| **Day 6** |  |  |  |  |  |
| Normal | 12,222 (100%, N: 12,264) | 387 (99%, N: 389) | 421 (99%, N: 425) | 10,453 (100%, N: 10,489) | 961 (100%, N: 961) |
| Grade 1 | 29 (0.2%, N: 12,264) | 1 (0.3%, N: 389) | 3 (0.7%, N: 425) | 25 (0.2%, N: 10,489) | 0 (0%, N: 961) |
| Grade 2 | 10 (<0.1%, N: 12,264) | 1 (0.3%, N: 389) | 1 (0.2%, N: 425) | 8 (<0.1%, N: 10,489) | 0 (0%, N: 961) |

* Grade 3 and 4 cases have been tracked and counted separately in the adverse events section

Table 43 Diarrhea systemic reaction during first week after first injection

| Characteristic | Overall, N = 24,056 | Fakhravac, Random, N = 824 | BBIBP-Corv2, Random, N = 832 | Fakhravac, Non-Random, N = 19,429 | BBIBP-Corv2, Non-Random, N = 2,971 |
| --- | --- | --- | --- | --- | --- |
| **Day 1** |  |  |  |  |  |
| Normal | 11,007 (100%, N: 11,025) | 418 (100%, N: 418) | 436 (100%, N: 437) | 9,593 (100%, N: 9,610) | 560 (100%, N: 560) |
| Grade 1 | 14 (0.1%, N: 11,025) | 0 (0%, N: 418) | 1 (0.2%, N: 437) | 13 (0.1%, N: 9,610) | 0 (0%, N: 560) |
| Grade 2 | 4 (<0.1%, N: 11,025) | 0 (0%, N: 418) | 0 (0%, N: 437) | 4 (<0.1%, N: 9,610) | 0 (0%, N: 560) |
| **Day 2** |  |  |  |  |  |
| Normal | 13,249 (100%, N: 13,266) | 413 (100%, N: 413) | 447 (100%, N: 447) | 11,567 (100%, N: 11,583) | 822 (100%, N: 823) |
| Grade 1 | 11 (<0.1%, N: 13,266) | 0 (0%, N: 413) | 0 (0%, N: 447) | 11 (<0.1%, N: 11,583) | 0 (0%, N: 823) |
| Grade 2 | 5 (<0.1%, N: 13,266) | 0 (0%, N: 413) | 0 (0%, N: 447) | 4 (<0.1%, N: 11,583) | 1 (0.1%, N: 823) |
| **Day 3** |  |  |  |  |  |
| Normal | 15,257 (100%, N: 15,278) | 431 (100%, N: 431) | 461 (100%, N: 463) | 13,203 (100%, N: 13,222) | 1,162 (100%, N: 1,162) |
| Grade 1 | 17 (0.1%, N: 15,278) | 0 (0%, N: 431) | 2 (0.4%, N: 463) | 15 (0.1%, N: 13,222) | 0 (0%, N: 1,162) |
| Grade 2 | 4 (<0.1%, N: 15,278) | 0 (0%, N: 431) | 0 (0%, N: 463) | 4 (<0.1%, N: 13,222) | 0 (0%, N: 1,162) |
| **Day 4** |  |  |  |  |  |
| Normal | 15,084 (100%, N: 15,098) | 371 (100%, N: 371) | 389 (100%, N: 390) | 13,208 (100%, N: 13,221) | 1,116 (100%, N: 1,116) |
| Grade 1 | 11 (<0.1%, N: 15,098) | 0 (0%, N: 371) | 1 (0.3%, N: 390) | 10 (<0.1%, N: 13,221) | 0 (0%, N: 1,116) |
| Grade 2 | 2 (<0.1%, N: 15,098) | 0 (0%, N: 371) | 0 (0%, N: 390) | 2 (<0.1%, N: 13,221) | 0 (0%, N: 1,116) |
| **Day 5** |  |  |  |  |  |
| Normal | 15,460 (100%, N: 15,475) | 362 (100%, N: 362) | 379 (100%, N: 379) | 13,553 (100%, N: 13,568) | 1,166 (100%, N: 1,166) |
| Grade 1 | 12 (<0.1%, N: 15,475) | 0 (0%, N: 362) | 0 (0%, N: 379) | 12 (<0.1%, N: 13,568) | 0 (0%, N: 1,166) |
| Grade 2 | 3 (<0.1%, N: 15,475) | 0 (0%, N: 362) | 0 (0%, N: 379) | 3 (<0.1%, N: 13,568) | 0 (0%, N: 1,166) |
| **Day 6** |  |  |  |  |  |
| Normal | 13,571 (100%, N: 13,581) | 350 (100%, N: 351) | 366 (100%, N: 366) | 11,906 (100%, N: 11,914) | 949 (100%, N: 950) |
| Grade 1 | 7 (<0.1%, N: 13,581) | 1 (0.3%, N: 351) | 0 (0%, N: 366) | 5 (<0.1%, N: 11,914) | 1 (0.1%, N: 950) |
| Grade 2 | 3 (<0.1%, N: 13,581) | 0 (0%, N: 351) | 0 (0%, N: 366) | 3 (<0.1%, N: 11,914) | 0 (0%, N: 950) |

* Grade 3 and 4 cases have been tracked and counted separately in the adverse events section

Table 44 Diarrhea systemic reaction during first week after second injection

| Characteristic | Overall, N = 24,056 | Fakhravac, Random, N = 824 | BBIBP-Corv2, Random, N = 832 | Fakhravac, Non-Random, N = 19,429 | BBIBP-Corv2, Non-Random, N = 2,971 |
| --- | --- | --- | --- | --- | --- |
| **Day 1** |  |  |  |  |  |
| Normal | 9,356 (100%, N: 9,373) | 353 (100%, N: 353) | 393 (100%, N: 393) | 8,159 (100%, N: 8,175) | 451 (100%, N: 452) |
| Grade 1 | 12 (0.1%, N: 9,373) | 0 (0%, N: 353) | 0 (0%, N: 393) | 11 (0.1%, N: 8,175) | 1 (0.2%, N: 452) |
| Grade 2 | 3 (<0.1%, N: 9,373) | 0 (0%, N: 353) | 0 (0%, N: 393) | 3 (<0.1%, N: 8,175) | 0 (0%, N: 452) |
| **Day 2** |  |  |  |  |  |
| Normal | 11,629 (100%, N: 11,646) | 399 (100%, N: 399) | 425 (100%, N: 426) | 10,080 (100%, N: 10,096) | 725 (100%, N: 725) |
| Grade 1 | 13 (0.1%, N: 11,646) | 0 (0%, N: 399) | 1 (0.2%, N: 426) | 12 (0.1%, N: 10,096) | 0 (0%, N: 725) |
| Grade 2 | 3 (<0.1%, N: 11,646) | 0 (0%, N: 399) | 0 (0%, N: 426) | 3 (<0.1%, N: 10,096) | 0 (0%, N: 725) |
| **Day 3** |  |  |  |  |  |
| Normal | 13,629 (100%, N: 13,639) | 432 (100%, N: 432) | 451 (100%, N: 452) | 11,776 (100%, N: 11,785) | 970 (100%, N: 970) |
| Grade 1 | 8 (<0.1%, N: 13,639) | 0 (0%, N: 432) | 1 (0.2%, N: 452) | 7 (<0.1%, N: 11,785) | 0 (0%, N: 970) |
| Grade 2 | 1 (<0.1%, N: 13,639) | 0 (0%, N: 432) | 0 (0%, N: 452) | 1 (<0.1%, N: 11,785) | 0 (0%, N: 970) |
| **Day 4** |  |  |  |  |  |
| Normal | 13,708 (100%, N: 13,718) | 427 (100%, N: 427) | 428 (100%, N: 428) | 11,839 (100%, N: 11,849) | 1,014 (100%, N: 1,014) |
| Grade 1 | 7 (<0.1%, N: 13,718) | 0 (0%, N: 427) | 0 (0%, N: 428) | 7 (<0.1%, N: 11,849) | 0 (0%, N: 1,014) |
| Grade 2 | 2 (<0.1%, N: 13,718) | 0 (0%, N: 427) | 0 (0%, N: 428) | 2 (<0.1%, N: 11,849) | 0 (0%, N: 1,014) |
| **Day 5** |  |  |  |  |  |
| Normal | 14,347 (100%, N: 14,359) | 442 (100%, N: 442) | 441 (100%, N: 441) | 12,317 (100%, N: 12,329) | 1,147 (100%, N: 1,147) |
| Grade 1 | 7 (<0.1%, N: 14,359) | 0 (0%, N: 442) | 0 (0%, N: 441) | 7 (<0.1%, N: 12,329) | 0 (0%, N: 1,147) |
| Grade 2 | 3 (<0.1%, N: 14,359) | 0 (0%, N: 442) | 0 (0%, N: 441) | 3 (<0.1%, N: 12,329) | 0 (0%, N: 1,147) |
| **Day 6** |  |  |  |  |  |
| Normal | 12,222 (100%, N: 12,232) | 387 (100%, N: 387) | 421 (100%, N: 422) | 10,453 (100%, N: 10,462) | 961 (100%, N: 961) |
| Grade 1 | 6 (<0.1%, N: 12,232) | 0 (0%, N: 387) | 0 (0%, N: 422) | 6 (<0.1%, N: 10,462) | 0 (0%, N: 961) |
| Grade 2 | 2 (<0.1%, N: 12,232) | 0 (0%, N: 387) | 1 (0.2%, N: 422) | 1 (<0.1%, N: 10,462) | 0 (0%, N: 961) |

* Grade 3 and 4 cases have been tracked and counted separately in the adverse events section

Table 45 Headache systemic reaction during first week after first injection

| Characteristic | Overall, N = 24,056 | Fakhravac, Random, N = 824 | BBIBP-Corv2, Random, N = 832 | Fakhravac, Non-Random, N = 19,429 | BBIBP-Corv2, Non-Random, N = 2,971 |
| --- | --- | --- | --- | --- | --- |
| **Day 1** |  |  |  |  |  |
| Normal | 11,007 (99%, N: 11,089) | 418 (100%, N: 418) | 436 (99%, N: 441) | 9,593 (99%, N: 9,665) | 560 (99%, N: 565) |
| Grade 1 | 44 (0.4%, N: 11,089) | 0 (0%, N: 418) | 3 (0.7%, N: 441) | 37 (0.4%, N: 9,665) | 4 (0.7%, N: 565) |
| Grade 2 | 33 (0.3%, N: 11,089) | 0 (0%, N: 418) | 2 (0.5%, N: 441) | 31 (0.3%, N: 9,665) | 0 (0%, N: 565) |
| **Day 2** |  |  |  |  |  |
| Normal | 13,249 (99%, N: 13,318) | 413 (100%, N: 414) | 447 (100%, N: 449) | 11,567 (99%, N: 11,626) | 822 (99%, N: 829) |
| Grade 1 | 46 (0.3%, N: 13,318) | 1 (0.2%, N: 414) | 2 (0.4%, N: 449) | 39 (0.3%, N: 11,626) | 4 (0.5%, N: 829) |
| Grade 2 | 20 (0.2%, N: 13,318) | 0 (0%, N: 414) | 0 (0%, N: 449) | 18 (0.2%, N: 11,626) | 2 (0.2%, N: 829) |
| **Day 3** |  |  |  |  |  |
| Normal | 15,257 (100%, N: 15,309) | 431 (99%, N: 434) | 461 (99%, N: 464) | 13,203 (100%, N: 13,245) | 1,162 (100%, N: 1,166) |
| Grade 1 | 28 (0.2%, N: 15,309) | 2 (0.5%, N: 434) | 1 (0.2%, N: 464) | 23 (0.2%, N: 13,245) | 2 (0.2%, N: 1,166) |
| Grade 2 | 15 (<0.1%, N: 15,309) | 1 (0.2%, N: 434) | 1 (0.2%, N: 464) | 12 (<0.1%, N: 13,245) | 1 (<0.1%, N: 1,166) |
| **Day 4** |  |  |  |  |  |
| Normal | 15,084 (100%, N: 15,140) | 371 (99%, N: 375) | 389 (100%, N: 390) | 13,208 (100%, N: 13,257) | 1,116 (100%, N: 1,118) |
| Grade 1 | 30 (0.2%, N: 15,140) | 2 (0.5%, N: 375) | 0 (0%, N: 390) | 26 (0.2%, N: 13,257) | 2 (0.2%, N: 1,118) |
| Grade 2 | 21 (0.1%, N: 15,140) | 2 (0.5%, N: 375) | 1 (0.3%, N: 390) | 18 (0.1%, N: 13,257) | 0 (0%, N: 1,118) |
| **Day 5** |  |  |  |  |  |
| Normal | 15,460 (100%, N: 15,500) | 362 (100%, N: 362) | 379 (100%, N: 380) | 13,553 (100%, N: 13,591) | 1,166 (100%, N: 1,167) |
| Grade 1 | 19 (0.1%, N: 15,500) | 0 (0%, N: 362) | 1 (0.3%, N: 380) | 17 (0.1%, N: 13,591) | 1 (<0.1%, N: 1,167) |
| Grade 2 | 16 (0.1%, N: 15,500) | 0 (0%, N: 362) | 0 (0%, N: 380) | 16 (0.1%, N: 13,591) | 0 (0%, N: 1,167) |
| **Day 6** |  |  |  |  |  |
| Normal | 13,571 (100%, N: 13,596) | 350 (100%, N: 351) | 366 (100%, N: 367) | 11,906 (100%, N: 11,928) | 949 (100%, N: 950) |
| Grade 1 | 9 (<0.1%, N: 13,596) | 0 (0%, N: 351) | 1 (0.3%, N: 367) | 8 (<0.1%, N: 11,928) | 0 (0%, N: 950) |
| Grade 2 | 9 (<0.1%, N: 13,596) | 1 (0.3%, N: 351) | 0 (0%, N: 367) | 8 (<0.1%, N: 11,928) | 0 (0%, N: 950) |

* Grade 3 and 4 cases have been tracked and counted separately in the adverse events section

Table 46 Headache systemic reaction during first week after second injection

| Characteristic | Overall, N = 24,056 | Fakhravac, Random, N = 824 | BBIBP-Corv2, Random, N = 832 | Fakhravac, Non-Random, N = 19,429 | BBIBP-Corv2, Non-Random, N = 2,971 |
| --- | --- | --- | --- | --- | --- |
| **Day 1** |  |  |  |  |  |
| Normal | 9,356 (99%, N: 9,422) | 353 (100%, N: 354) | 393 (100%, N: 394) | 8,159 (99%, N: 8,222) | 451 (100%, N: 452) |
| Grade 1 | 35 (0.4%, N: 9,422) | 0 (0%, N: 354) | 0 (0%, N: 394) | 34 (0.4%, N: 8,222) | 1 (0.2%, N: 452) |
| Grade 2 | 25 (0.3%, N: 9,422) | 1 (0.3%, N: 354) | 1 (0.3%, N: 394) | 23 (0.3%, N: 8,222) | 0 (0%, N: 452) |
| **Day 2** |  |  |  |  |  |
| Normal | 11,629 (100%, N: 11,673) | 399 (100%, N: 400) | 425 (100%, N: 426) | 10,080 (100%, N: 10,121) | 725 (100%, N: 726) |
| Grade 1 | 20 (0.2%, N: 11,673) | 0 (0%, N: 400) | 0 (0%, N: 426) | 20 (0.2%, N: 10,121) | 0 (0%, N: 726) |
| Grade 2 | 18 (0.2%, N: 11,673) | 1 (0.2%, N: 400) | 1 (0.2%, N: 426) | 16 (0.2%, N: 10,121) | 0 (0%, N: 726) |
| **Day 3** |  |  |  |  |  |
| Normal | 13,629 (100%, N: 13,665) | 432 (99%, N: 435) | 451 (99%, N: 455) | 11,776 (100%, N: 11,803) | 970 (100%, N: 972) |
| Grade 1 | 15 (0.1%, N: 13,665) | 2 (0.5%, N: 435) | 0 (0%, N: 455) | 12 (0.1%, N: 11,803) | 1 (0.1%, N: 972) |
| Grade 2 | 16 (0.1%, N: 13,665) | 1 (0.2%, N: 435) | 4 (0.9%, N: 455) | 10 (<0.1%, N: 11,803) | 1 (0.1%, N: 972) |
| **Day 4** |  |  |  |  |  |
| Normal | 13,708 (100%, N: 13,738) | 427 (99%, N: 430) | 428 (100%, N: 429) | 11,839 (100%, N: 11,864) | 1,014 (100%, N: 1,015) |
| Grade 1 | 12 (<0.1%, N: 13,738) | 2 (0.5%, N: 430) | 1 (0.2%, N: 429) | 9 (<0.1%, N: 11,864) | 0 (0%, N: 1,015) |
| Grade 2 | 14 (0.1%, N: 13,738) | 1 (0.2%, N: 430) | 0 (0%, N: 429) | 12 (0.1%, N: 11,864) | 1 (<0.1%, N: 1,015) |
| **Day 5** |  |  |  |  |  |
| Normal | 14,347 (100%, N: 14,376) | 442 (99%, N: 445) | 441 (100%, N: 443) | 12,317 (100%, N: 12,340) | 1,147 (100%, N: 1,148) |
| Grade 1 | 12 (<0.1%, N: 14,376) | 1 (0.2%, N: 445) | 1 (0.2%, N: 443) | 10 (<0.1%, N: 12,340) | 0 (0%, N: 1,148) |
| Grade 2 | 15 (0.1%, N: 14,376) | 2 (0.4%, N: 445) | 1 (0.2%, N: 443) | 11 (<0.1%, N: 12,340) | 1 (<0.1%, N: 1,148) |
| **Day 6** |  |  |  |  |  |
| Normal | 12,222 (100%, N: 12,242) | 387 (99%, N: 389) | 421 (100%, N: 423) | 10,453 (100%, N: 10,469) | 961 (100%, N: 961) |
| Grade 1 | 10 (<0.1%, N: 12,242) | 1 (0.3%, N: 389) | 0 (0%, N: 423) | 9 (<0.1%, N: 10,469) | 0 (0%, N: 961) |
| Grade 2 | 8 (<0.1%, N: 12,242) | 0 (0%, N: 389) | 2 (0.5%, N: 423) | 6 (<0.1%, N: 10,469) | 0 (0%, N: 961) |

* Grade 3 and 4 cases have been tracked and counted separately in the adverse events section

Table 47 Fatigue systemic reaction during first week after first injection

| Characteristic | Overall, N = 24,056 | Fakhravac, Random, N = 824 | BBIBP-Corv2, Random, N = 832 | Fakhravac, Non-Random, N = 19,429 | BBIBP-Corv2, Non-Random, N = 2,971 |
| --- | --- | --- | --- | --- | --- |
| **Day 1** |  |  |  |  |  |
| Normal | 11,007 (99%, N: 11,096) | 418 (100%, N: 418) | 436 (99%, N: 440) | 9,593 (99%, N: 9,671) | 560 (99%, N: 567) |
| Grade 1 | 38 (0.3%, N: 11,096) | 0 (0%, N: 418) | 1 (0.2%, N: 440) | 35 (0.4%, N: 9,671) | 2 (0.4%, N: 567) |
| Grade 2 | 46 (0.4%, N: 11,096) | 0 (0%, N: 418) | 3 (0.7%, N: 440) | 38 (0.4%, N: 9,671) | 5 (0.9%, N: 567) |
| **Day 2** |  |  |  |  |  |
| Normal | 13,249 (99%, N: 13,322) | 413 (100%, N: 414) | 447 (100%, N: 449) | 11,567 (99%, N: 11,631) | 822 (99%, N: 828) |
| Grade 1 | 31 (0.2%, N: 13,322) | 1 (0.2%, N: 414) | 1 (0.2%, N: 449) | 28 (0.2%, N: 11,631) | 1 (0.1%, N: 828) |
| Grade 2 | 33 (0.2%, N: 13,322) | 0 (0%, N: 414) | 0 (0%, N: 449) | 28 (0.2%, N: 11,631) | 5 (0.6%, N: 828) |
| **Day 3** |  |  |  |  |  |
| Normal | 15,257 (100%, N: 15,309) | 431 (99%, N: 436) | 461 (100%, N: 463) | 13,203 (100%, N: 13,244) | 1,162 (100%, N: 1,166) |
| Grade 1 | 16 (0.1%, N: 15,309) | 1 (0.2%, N: 436) | 0 (0%, N: 463) | 14 (0.1%, N: 13,244) | 1 (<0.1%, N: 1,166) |
| Grade 2 | 27 (0.2%, N: 15,309) | 3 (0.7%, N: 436) | 1 (0.2%, N: 463) | 20 (0.2%, N: 13,244) | 3 (0.3%, N: 1,166) |
| **Day 4** |  |  |  |  |  |
| Normal | 15,084 (100%, N: 15,130) | 371 (99%, N: 375) | 389 (99%, N: 391) | 13,208 (100%, N: 13,247) | 1,116 (100%, N: 1,117) |
| Grade 1 | 19 (0.1%, N: 15,130) | 0 (0%, N: 375) | 0 (0%, N: 391) | 18 (0.1%, N: 13,247) | 1 (<0.1%, N: 1,117) |
| Grade 2 | 19 (0.1%, N: 15,130) | 3 (0.8%, N: 375) | 2 (0.5%, N: 391) | 14 (0.1%, N: 13,247) | 0 (0%, N: 1,117) |
| **Day 5** |  |  |  |  |  |
| Normal | 15,460 (100%, N: 15,493) | 362 (100%, N: 363) | 379 (100%, N: 379) | 13,553 (100%, N: 13,583) | 1,166 (100%, N: 1,168) |
| Grade 1 | 9 (<0.1%, N: 15,493) | 0 (0%, N: 363) | 0 (0%, N: 379) | 7 (<0.1%, N: 13,583) | 2 (0.2%, N: 1,168) |
| Grade 2 | 20 (0.1%, N: 15,493) | 1 (0.3%, N: 363) | 0 (0%, N: 379) | 19 (0.1%, N: 13,583) | 0 (0%, N: 1,168) |
| **Day 6** |  |  |  |  |  |
| Normal | 13,571 (100%, N: 13,598) | 350 (100%, N: 351) | 366 (100%, N: 366) | 11,906 (100%, N: 11,929) | 949 (100%, N: 952) |
| Grade 1 | 9 (<0.1%, N: 13,598) | 1 (0.3%, N: 351) | 0 (0%, N: 366) | 7 (<0.1%, N: 11,929) | 1 (0.1%, N: 952) |
| Grade 2 | 14 (0.1%, N: 13,598) | 0 (0%, N: 351) | 0 (0%, N: 366) | 12 (0.1%, N: 11,929) | 2 (0.2%, N: 952) |

* Grade 3 and 4 cases have been tracked and counted separately in the adverse events section

Table 48 Fatigue systemic reaction during first week after second injection

| Characteristic | Overall, N = 24,056 | Fakhravac, Random, N = 824 | BBIBP-Corv2, Random, N = 832 | Fakhravac, Non-Random, N = 19,429 | BBIBP-Corv2, Non-Random, N = 2,971 |
| --- | --- | --- | --- | --- | --- |
| **Day 1** |  |  |  |  |  |
| Normal | 9,356 (99%, N: 9,435) | 353 (99%, N: 355) | 393 (99%, N: 395) | 8,159 (99%, N: 8,233) | 451 (100%, N: 452) |
| Grade 1 | 30 (0.3%, N: 9,435) | 1 (0.3%, N: 355) | 1 (0.3%, N: 395) | 27 (0.3%, N: 8,233) | 1 (0.2%, N: 452) |
| Grade 2 | 44 (0.5%, N: 9,435) | 1 (0.3%, N: 355) | 1 (0.3%, N: 395) | 42 (0.5%, N: 8,233) | 0 (0%, N: 452) |
| **Day 2** |  |  |  |  |  |
| Normal | 11,629 (100%, N: 11,678) | 399 (100%, N: 400) | 425 (100%, N: 426) | 10,080 (100%, N: 10,126) | 725 (100%, N: 726) |
| Grade 1 | 17 (0.1%, N: 11,678) | 1 (0.2%, N: 400) | 0 (0%, N: 426) | 16 (0.2%, N: 10,126) | 0 (0%, N: 726) |
| Grade 2 | 27 (0.2%, N: 11,678) | 0 (0%, N: 400) | 1 (0.2%, N: 426) | 25 (0.2%, N: 10,126) | 1 (0.1%, N: 726) |
| **Day 3** |  |  |  |  |  |
| Normal | 13,629 (100%, N: 13,666) | 432 (99%, N: 435) | 451 (100%, N: 452) | 11,776 (100%, N: 11,807) | 970 (100%, N: 972) |
| Grade 1 | 15 (0.1%, N: 13,666) | 2 (0.5%, N: 435) | 0 (0%, N: 452) | 13 (0.1%, N: 11,807) | 0 (0%, N: 972) |
| Grade 2 | 20 (0.1%, N: 13,666) | 1 (0.2%, N: 435) | 1 (0.2%, N: 452) | 16 (0.1%, N: 11,807) | 2 (0.2%, N: 972) |
| **Day 4** |  |  |  |  |  |
| Normal | 13,708 (100%, N: 13,737) | 427 (100%, N: 429) | 428 (100%, N: 428) | 11,839 (100%, N: 11,866) | 1,014 (100%, N: 1,014) |
| Grade 1 | 9 (<0.1%, N: 13,737) | 1 (0.2%, N: 429) | 0 (0%, N: 428) | 8 (<0.1%, N: 11,866) | 0 (0%, N: 1,014) |
| Grade 2 | 17 (0.1%, N: 13,737) | 1 (0.2%, N: 429) | 0 (0%, N: 428) | 16 (0.1%, N: 11,866) | 0 (0%, N: 1,014) |
| **Day 5** |  |  |  |  |  |
| Normal | 14,347 (100%, N: 14,374) | 442 (100%, N: 444) | 441 (100%, N: 442) | 12,317 (100%, N: 12,341) | 1,147 (100%, N: 1,147) |
| Grade 1 | 13 (<0.1%, N: 14,374) | 1 (0.2%, N: 444) | 0 (0%, N: 442) | 12 (<0.1%, N: 12,341) | 0 (0%, N: 1,147) |
| Grade 2 | 13 (<0.1%, N: 14,374) | 1 (0.2%, N: 444) | 0 (0%, N: 442) | 12 (<0.1%, N: 12,341) | 0 (0%, N: 1,147) |
| **Day 6** |  |  |  |  |  |
| Normal | 12,222 (100%, N: 12,247) | 387 (99%, N: 389) | 421 (100%, N: 423) | 10,453 (100%, N: 10,474) | 961 (100%, N: 961) |
| Grade 1 | 15 (0.1%, N: 12,247) | 1 (0.3%, N: 389) | 1 (0.2%, N: 423) | 13 (0.1%, N: 10,474) | 0 (0%, N: 961) |
| Grade 2 | 9 (<0.1%, N: 12,247) | 1 (0.3%, N: 389) | 0 (0%, N: 423) | 8 (<0.1%, N: 10,474) | 0 (0%, N: 961) |

* Grade 3 and 4 cases have been tracked and counted separately in the adverse events section

Table 49 Muscle pain systemic reaction during first week after first injection

| Characteristic | Overall, N = 24,056 | Fakhravac, Random, N = 824 | BBIBP-Corv2, Random, N = 832 | Fakhravac, Non-Random, N = 19,429 | BBIBP-Corv2, Non-Random, N = 2,971 |
| --- | --- | --- | --- | --- | --- |
| **Day 1** |  |  |  |  |  |
| Normal | 11,007 (100%, N: 11,039) | 418 (100%, N: 419) | 436 (100%, N: 438) | 9,593 (100%, N: 9,621) | 560 (100%, N: 561) |
| Grade 1 | 17 (0.2%, N: 11,039) | 1 (0.2%, N: 419) | 1 (0.2%, N: 438) | 14 (0.1%, N: 9,621) | 1 (0.2%, N: 561) |
| Grade 2 | 14 (0.1%, N: 11,039) | 0 (0%, N: 419) | 1 (0.2%, N: 438) | 13 (0.1%, N: 9,621) | 0 (0%, N: 561) |
| **Day 2** |  |  |  |  |  |
| Normal | 13,249 (100%, N: 13,274) | 413 (100%, N: 413) | 447 (100%, N: 447) | 11,567 (100%, N: 11,592) | 822 (100%, N: 822) |
| Grade 1 | 8 (<0.1%, N: 13,274) | 0 (0%, N: 413) | 0 (0%, N: 447) | 8 (<0.1%, N: 11,592) | 0 (0%, N: 822) |
| Grade 2 | 12 (<0.1%, N: 13,274) | 0 (0%, N: 413) | 0 (0%, N: 447) | 12 (0.1%, N: 11,592) | 0 (0%, N: 822) |
| **Day 3** |  |  |  |  |  |
| Normal | 15,257 (100%, N: 15,277) | 431 (100%, N: 431) | 461 (100%, N: 462) | 13,203 (100%, N: 13,221) | 1,162 (100%, N: 1,163) |
| Grade 1 | 6 (<0.1%, N: 15,277) | 0 (0%, N: 431) | 0 (0%, N: 462) | 6 (<0.1%, N: 13,221) | 0 (0%, N: 1,163) |
| Grade 2 | 10 (<0.1%, N: 15,277) | 0 (0%, N: 431) | 1 (0.2%, N: 462) | 8 (<0.1%, N: 13,221) | 1 (<0.1%, N: 1,163) |
| **Day 4** |  |  |  |  |  |
| Normal | 15,084 (100%, N: 15,103) | 371 (100%, N: 372) | 389 (100%, N: 390) | 13,208 (100%, N: 13,223) | 1,116 (100%, N: 1,118) |
| Grade 1 | 9 (<0.1%, N: 15,103) | 0 (0%, N: 372) | 0 (0%, N: 390) | 7 (<0.1%, N: 13,223) | 2 (0.2%, N: 1,118) |
| Grade 2 | 7 (<0.1%, N: 15,103) | 1 (0.3%, N: 372) | 0 (0%, N: 390) | 6 (<0.1%, N: 13,223) | 0 (0%, N: 1,118) |
| **Day 5** |  |  |  |  |  |
| Normal | 15,460 (100%, N: 15,474) | 362 (100%, N: 362) | 379 (100%, N: 379) | 13,553 (100%, N: 13,565) | 1,166 (100%, N: 1,168) |
| Grade 1 | 6 (<0.1%, N: 15,474) | 0 (0%, N: 362) | 0 (0%, N: 379) | 4 (<0.1%, N: 13,565) | 2 (0.2%, N: 1,168) |
| Grade 2 | 7 (<0.1%, N: 15,474) | 0 (0%, N: 362) | 0 (0%, N: 379) | 7 (<0.1%, N: 13,565) | 0 (0%, N: 1,168) |
| **Day 6** |  |  |  |  |  |
| Normal | 13,571 (100%, N: 13,595) | 350 (100%, N: 350) | 366 (100%, N: 367) | 11,906 (100%, N: 11,927) | 949 (100%, N: 951) |
| Grade 1 | 9 (<0.1%, N: 13,595) | 0 (0%, N: 350) | 0 (0%, N: 367) | 8 (<0.1%, N: 11,927) | 1 (0.1%, N: 951) |
| Grade 2 | 10 (<0.1%, N: 13,595) | 0 (0%, N: 350) | 1 (0.3%, N: 367) | 9 (<0.1%, N: 11,927) | 0 (0%, N: 951) |

* Grade 3 and 4 cases have been tracked and counted separately in the adverse events section

Table 50 Muscle pain systemic reaction during first week after second injection

| Characteristic | Overall, N = 24,056 | Fakhravac, Random, N = 824 | BBIBP-Corv2, Random, N = 832 | Fakhravac, Non-Random, N = 19,429 | BBIBP-Corv2, Non-Random, N = 2,971 |
| --- | --- | --- | --- | --- | --- |
| **Day 1** |  |  |  |  |  |
| Normal | 9,356 (99%, N: 9,435) | 353 (99%, N: 355) | 393 (99%, N: 395) | 8,159 (99%, N: 8,233) | 451 (100%, N: 452) |
| Grade 1 | 34 (0.3%, N: 9,435) | 1 (0.3%, N: 355) | 1 (0.3%, N: 395) | 24 (0.3%, N: 8,233) | 2 (0.2%, N: 452) |
| Grade 2 | 40 (0.5%, N: 9,435) | 1 (0.3%, N: 355) | 1 (0.3%, N: 395) | 45 (0.5%, N: 8,233) | 0 (0%, N: 452) |
| **Day 2** |  |  |  |  |  |
| Normal | 11,629 (100%, N: 11,678) | 399 (100%, N: 400) | 425 (100%, N: 426) | 10,080 (100%, N: 10,126) | 725 (100%, N: 726) |
| Grade 1 | 16 (0.1%, N: 11,678) | 1 (0.2%, N: 400) | 0 (0%, N: 426) | 13 (0.2%, N: 10,126) | 0 (0%, N: 726) |
| Grade 2 | 29 (0.2%, N: 11,678) | 0 (0%, N: 400) | 1 (0.2%, N: 426) | 15 (0.2%, N: 10,126) | 1 (0.1%, N: 726) |
| **Day 3** |  |  |  |  |  |
| Normal | 13,629 (100%, N: 13,666) | 432 (99%, N: 435) | 451 (100%, N: 452) | 11,776 (100%, N: 11,807) | 970 (100%, N: 972) |
| Grade 1 | 15 (0.1%, N: 13,666) | 2 (0.5%, N: 435) | 0 (0%, N: 452) | 15 (0.1%, N: 11,807) | 0 (0%, N: 972) |
| Grade 2 | 21 (0.1%, N: 13,666) | 1 (0.2%, N: 435) | 1 (0.2%, N: 452) | 16 (0.1%, N: 11,807) | 2 (0.2%, N: 972) |
| **Day 4** |  |  |  |  |  |
| Normal | 13,708 (100%, N: 13,737) | 427 (100%, N: 429) | 428 (100%, N: 428) | 11,839 (100%, N: 11,866) | 1,014 (100%, N: 1,014) |
| Grade 1 | 9 (<0.1%, N: 13,737) | 1 (0.2%, N: 429) | 0 (0%, N: 428) | 8 (<0.1%, N: 11,866) | 0 (0%, N: 1,014) |
| Grade 2 | 17 (0.1%, N: 13,737) | 1 (0.2%, N: 429) | 0 (0%, N: 428) | 17 (0.1%, N: 11,866) | 0 (0%, N: 1,014) |
| **Day 5** |  |  |  |  |  |
| Normal | 14,347 (100%, N: 14,374) | 442 (100%, N: 444) | 441 (100%, N: 442) | 12,317 (100%, N: 12,341) | 1,147 (100%, N: 1,147) |
| Grade 1 | 13 (<0.1%, N: 14,374) | 1 (0.2%, N: 444) | 0 (0%, N: 442) | 15 (<0.1%, N: 12,341) | 0 (0%, N: 1,147) |
| Grade 2 | 14 (<0.1%, N: 14,374) | 1 (0.2%, N: 444) | 0 (0%, N: 442) | 12 (<0.1%, N: 12,341) | 0 (0%, N: 1,147) |
| **Day 6** |  |  |  |  |  |
| Normal | 12,221 (100%, N: 12,246) | 387 (99%, N: 389) | 421 (100%, N: 423) | 10,452 (100%, N: 10,473) | 961 (100%, N: 961) |
| Grade 1 | 15 (0.1%, N: 12,246) | 1 (0.3%, N: 389) | 1 (0.2%, N: 423) | 14 (0.1%, N: 10,473) | 0 (0%, N: 961) |
| Grade 2 | 9 (<0.1%, N: 12,246) | 1 (0.3%, N: 389) | 0 (0%, N: 423) | 8 (<0.1%, N: 10,473) | 0 (0%, N: 961) |

* Grade 3 and 4 cases have been tracked and counted separately in the adverse events section

## List of all adverse events during four month active follow-up

Table 51 Final classification of adverse events that are judged by the follow-up team to be somehow related to the vaccines recieved during four month active follow-up in study groups

| Final ICD-10 | Fakhravac Random | BBIBP-Corv2 Random | Fakhravac Non_Random | BBIBP-Corv2 Non_Random | Total |
| --- | --- | --- | --- | --- | --- |
| I95: Hypotension |  |  |  | 1 | 1 |
| I95.2: Hypotension due to drugs |  |  | 1 |  | 1 |
| L29.9: Pruritus, unspecified |  |  | 1 |  | 1 |
| L50.9: Urticaria, unspecified | 1 |  |  |  | 1 |
| M25.5: Pain in joint |  |  | 1 |  | 1 |
| N92: Excessive, frequent and irregular menstruation |  |  | 1 |  | 1 |
| N94.6: Dysmenorrhoea, unspecified |  |  | 1 |  | 1 |
| R22.9: Localized swelling, mass and lump, unspecified |  | 1 |  |  | 1 |
| R23.3: Spontaneous ecchymoses |  |  | 1 |  | 1 |
| R50.8: Other specified fever |  |  | 1 |  | 1 |
| R55: Syncope and collapse |  |  |  | 1 | 1 |
| R60: Oedema, not elsewhere classified |  |  | 1 |  | 1 |
| R60.9: Oedema, unspecified |  |  | 1 |  | 1 |
| T78.2: Anaphylactic shock, unspecified |  |  | 1 |  | 1 |
| B00: Herpesviral [herpes simplex] infections |  |  | 1 |  | 1 |
| FAL: False Alarm |  |  | 1 | 1 | 2 |
| L29: Pruritus |  |  | 1 | 1 | 2 |
| R21: Rash and other nonspecific skin eruption | 1 |  | 1 |  | 2 |
| T78.4: Allergy, unspecified |  |  | 1 | 1 | 2 |
| R42: Dizziness and giddiness(rate*) |  | 1 (0.73) | 8 (0.31) |  | 9 (0.28) |
| R50: Fever of other and unknown origin (rate*) | 3 (2.17) | 3 (2.2) | 3 (0.12) |  | 9 (0.28) |
| L50: Urticaria (rate*) |  |  | 12 (0.47) |  | 12 (0.38) |
| R51: Headache (rate*) | 5 (3.62) | 8 (5.87) | 71 (2.75) | 4 (1.21) | 88 (2.76) |
| FAG: False Grade (rate*) | 11 (7.97) | 9 (6.6) | 107 (4.15) | 5 (1.51) | 132 (4.15) |
| R52: Pain, not elsewhere classified (rate*) | 23 (16.66) | 14 (10.27) | 415 (16.1) | 11 (3.33) | 463 (14.55) |
| Total (rate*) | 44 (31.86) | 36 (26.42) | 631 (24.48) | 25 (7.56) | 736 (23.12) |
| Total Person-Time | 138084 | 136271 | 2577784 | 330651 | 3182790 |

*rate per 100000 person-day

Table 52 All allergic related adverse events during four month active follow-up in study groups

|  | Fakhravac Random | BBIBP-Corv2 Random | Fakhravac Non_Random | BBIBP-Corv2 Non_Random | Total  Fakhravac | Total BBIBP-Corv2 |
| --- | --- | --- | --- | --- | --- | --- |
| All allergic related events (rate*)  L29.9: Pruritus, unspecified  L50.9: Urticaria, unspecified  R60: Oedema, not elsewhere classified  R60.9: Oedema, unspecified  T78.2: Anaphylactic shock, unspecified  L29: Pruritus  R21: Rash and other nonspecific skin eruption  T78.4: Allergy, unspecified  L50: Urticaria | 2 (1.44) | 0 (0) | 19 (0.74) | 2 (0.60) | 21 (0.77) | 2 (0.43) |
| Total Person-Time | 138084 | 136271 | 2577784 | 330651 | 2715868 | 466922 |

Table 53 Final classification of all adverse events by the first three letters of the ICD-10 code during four month active follow-up in study groups

| Final ICD-10 | Fakhravac Random | BBIBP-Corv2 Random | Fakhravac Non_Random | BBIBP-Corv2 Non_Random | Total |
| --- | --- | --- | --- | --- | --- |
| A04: Other bacterial intestinal infections |  |  | 1 |  | 1 |
| A05: Other bacterial foodborne intoxications, not elsewhere classified |  |  | 1 |  | 1 |
| B07: Viral warts |  |  | 1 |  | 1 |
| B37: Candidiasis |  |  | 1 |  | 1 |
| B98: Other specified infectious agents as the cause of diseases classified to other chapters |  |  | 1 |  | 1 |
| D34: Benign neoplasm of thyroid gland |  |  | 1 |  | 1 |
| D35: Benign neoplasm of other and unspecified endocrine glands | 1 |  |  |  | 1 |
| D72: Other disorders of white blood cells |  |  | 1 |  | 1 |
| E10-E14: Diabetes mellitus |  |  | 1 |  | 1 |
| E14: Unspecified diabetes mellitus |  |  | 1 |  | 1 |
| E23: Hypofunction and other disorders of pituitary gland |  |  | 1 |  | 1 |
| E34: Other endocrine disorders |  |  |  | 1 | 1 |
| E66: Obesity |  |  | 1 |  | 1 |
| E78: Disorders of lipoprotein metabolism and other lipidaemias |  |  | 1 |  | 1 |
| F39: Unspecified mood [affective] disorder |  |  |  | 1 | 1 |
| F45: Somatoform disorders |  |  | 1 |  | 1 |
| F52: Sexual dysfunction, not caused by organic disorder or disease |  |  | 1 |  | 1 |
| F60: Specific personality disorders |  |  | 1 |  | 1 |
| F99: Mental disorder, not otherwise specified |  |  |  | 1 | 1 |
| G00: Bacterial meningitis, not elsewhere classified |  |  | 1 |  | 1 |
| G25: Other extrapyramidal and movement disorders |  |  | 1 |  | 1 |
| G40: Epilepsy |  |  |  | 1 | 1 |
| G45: Transient cerebral ischaemic attacks and related syndromes |  |  | 1 |  | 1 |
| G51: Facial nerve disorders |  |  | 1 |  | 1 |
| H34: Retinal vascular occlusions |  |  | 1 |  | 1 |
| H52: Disorders of refraction and accommodation |  |  | 1 |  | 1 |
| H53: Visual disturbances |  |  | 1 |  | 1 |
| H62: Disorders of external ear in diseases classified elsewhere |  |  | 1 |  | 1 |
| H91: Other hearing loss |  |  | 1 |  | 1 |
| H93: Other disorders of ear, not elsewhere classified |  |  | 1 |  | 1 |
| I15: Secondary hypertension |  |  | 1 |  | 1 |
| I26: Pulmonary embolism |  | 1 |  |  | 1 |
| I61: Intracerebral haemorrhage |  | 1 |  |  | 1 |
| I86: Varicose veins of other sites |  |  | 1 |  | 1 |
| I88: Nonspecific lymphadenitis |  |  |  | 1 | 1 |
| J09: Influenza due to certain identified influenza virus |  |  | 1 |  | 1 |
| J15: Bacterial pneumonia, not elsewhere classified |  |  | 1 |  | 1 |
| J34: Other disorders of nose and nasal sinuses |  |  | 1 |  | 1 |
| J40: Bronchitis, not specified as acute or chronic |  |  | 1 |  | 1 |
| K00: Disorders of tooth development and eruption | 1 |  |  |  | 1 |
| K04: Diseases of pulp and periapical tissues |  |  | 1 |  | 1 |
| K06: Other disorders of gingiva and edentulous alveolar ridge |  |  | 1 |  | 1 |
| K11: Diseases of salivary glands |  | 1 |  |  | 1 |
| K31: Other diseases of stomach and duodenum |  |  | 1 |  | 1 |
| K40: Inguinal hernia |  |  | 1 |  | 1 |
| K42: Umbilical hernia |  |  | 1 |  | 1 |
| K50: Crohn disease [regional enteritis] |  |  | 1 |  | 1 |
| K50-K52: Noninfective enteritis and colitis |  |  |  | 1 | 1 |
| K60: Fissure and fistula of anal and rectal regions |  |  | 1 |  | 1 |
| K61: Abscess of anal and rectal regions |  |  | 1 |  | 1 |
| K70: Alcoholic liver disease |  |  | 1 |  | 1 |
| K74: Fibrosis and cirrhosis of liver |  |  |  | 1 | 1 |
| K76: Other diseases of liver |  |  | 1 |  | 1 |
| L03: Cellulitis |  |  | 1 |  | 1 |
| L05: Pilonidal cyst |  |  | 1 |  | 1 |
| L23: Allergic contact dermatitis |  |  | 1 |  | 1 |
| L30: Other dermatitis |  |  | 1 |  | 1 |
| L40: Psoriasis | 1 |  |  |  | 1 |
| L42: Pityriasis rosea |  |  | 1 |  | 1 |
| L64: Androgenic alopecia |  |  |  | 1 | 1 |
| M13: Other arthritis |  | 1 |  |  | 1 |
| M17: Gonarthrosis [arthrosis of knee] |  |  | 1 |  | 1 |
| M46: Other inflammatory spondylopathies |  |  |  | 1 | 1 |
| N13: Obstructive and reflux uropathy |  |  | 1 |  | 1 |
| N40: Hyperplasia of prostate |  |  | 1 |  | 1 |
| N48: Other disorders of penis |  |  | 1 |  | 1 |
| N50: Other disorders of male genital organs |  |  | 1 |  | 1 |
| N61: Inflammatory disorders of breast |  |  | 1 |  | 1 |
| N63: Unspecified lump in breast |  |  | 1 |  | 1 |
| N80: Endometriosis |  |  | 1 |  | 1 |
| N83: Noninflammatory disorders of ovary, fallopian tube and broad ligament |  |  | 1 |  | 1 |
| O00-O99: Pregnancy, childbirth and the puerperium |  |  | 1 |  | 1 |
| R12: Heartburn |  |  | 1 |  | 1 |
| R14: Flatulence and related conditions |  |  | 1 |  | 1 |
| R18: Ascites |  |  |  | 1 | 1 |
| R22: Localized swelling, mass and lump of skin and subcutaneous tissue |  | 1 |  |  | 1 |
| R41: Other symptoms and signs involving cognitive functions and awareness |  | 1 |  |  | 1 |
| R96: Other sudden death, cause unknown |  |  | 1 |  | 1 |
| S02: Fracture of skull and facial bones |  |  | 1 |  | 1 |
| S27: Injury of other and unspecified intrathoracic organs |  |  |  | 1 | 1 |
| S36: Injury of intra-abdominal organs |  |  | 1 |  | 1 |
| S52: Fracture of forearm |  | 1 |  |  | 1 |
| S60: Superficial injury of wrist and hand |  |  | 1 |  | 1 |
| S76: Injury of muscle and tendon at hip and thigh level |  |  | 1 |  | 1 |
| S93: Dislocation, sprain and strain of joints and ligaments at ankle and foot level |  |  |  | 1 | 1 |
| T14: Injury of unspecified body region |  |  | 1 |  | 1 |
| T23: Burn and corrosion of wrist and hand |  |  | 1 |  | 1 |
| T88: Other complications of surgical and medical care, not elsewhere classified |  |  | 1 |  | 1 |
| V03: Pedestrian injured in collision with car, pick-up truck or van |  |  | 1 |  | 1 |
| V29: Motorcycle rider injured in other and unspecified transport accidents |  |  | 1 |  | 1 |
| X47: Accidental poisoning by and exposure to other gases and vapours |  |  | 1 |  | 1 |
| Y58: Bacterial vaccines |  |  | 1 |  | 1 |
| Z10: Routine general health check-up of defined subpopulation |  |  | 1 |  | 1 |
| Z30: Contraceptive management |  |  | 1 |  | 1 |
| Z33: Pregnant state, incidental |  |  | 1 |  | 1 |
| Z34: Supervision of normal pregnancy |  |  | 1 |  | 1 |
| Z41: Procedures for purposes other than remedying health state |  | 1 |  |  | 1 |
| Z47: Other orthopaedic follow-up care |  | 1 |  |  | 1 |
| Z51: Other medical care |  |  | 1 |  | 1 |
| Z86: Personal history of certain other diseases |  |  |  | 1 | 1 |
| Z97: Presence of other devices |  |  | 1 |  | 1 |
| A08: Viral and other specified intestinal infections |  |  | 2 |  | 2 |
| B00: Herpesviral [herpes simplex] infections |  | 1 | 1 |  | 2 |
| E00-E07: Disorders of thyroid gland |  |  |  | 2 | 2 |
| E16: Other disorders of pancreatic internal secretion |  |  | 2 |  | 2 |
| F32: Depressive episode |  |  | 2 |  | 2 |
| H11: Other disorders of conjunctiva |  | 2 |  |  | 2 |
| H26: Other cataract |  |  | 2 |  | 2 |
| H40: Glaucoma |  |  | 2 |  | 2 |
| H54: Visual impairment including blindness (binocular or monocular) |  |  | 2 |  | 2 |
| H61: Other disorders of external ear |  |  | 2 |  | 2 |
| H67: Otitis media in diseases classified elsewhere |  |  | 1 | 1 | 2 |
| H92: Otalgia and effusion of ear |  | 1 | 1 |  | 2 |
| I50: Heart failure |  |  | 2 |  | 2 |
| J03: Acute tonsillitis |  |  | 2 |  | 2 |
| J11: Influenza, virus not identified | 1 |  | 1 |  | 2 |
| K25: Gastric ulcer |  |  | 2 |  | 2 |
| K58: Irritable bowel syndrome |  |  | 2 |  | 2 |
| K64: Haemorrhoids and perianal venous thrombosis |  |  | 2 |  | 2 |
| K81: Cholecystitis |  |  | 2 |  | 2 |
| K92: Other diseases of digestive system |  |  | 1 | 1 | 2 |
| L04: Acute lymphadenitis |  |  | 2 |  | 2 |
| L93: Lupus erythematosus |  |  | 1 | 1 | 2 |
| L98: Other disorders of skin and subcutaneous tissue, not elsewhere classified |  |  |  | 2 | 2 |
| M10: Gout |  |  | 2 |  | 2 |
| M51: Other intervertebral disc disorders | 1 |  | 1 |  | 2 |
| M79: Other soft tissue disorders, not elsewhere classified |  |  | 2 |  | 2 |
| M95: Other acquired deformities of musculoskeletal system and connective tissue |  |  | 1 | 1 | 2 |
| N30: Cystitis | 1 |  | 1 |  | 2 |
| N41: Inflammatory diseases of prostate |  |  | 2 |  | 2 |
| N64: Other disorders of breast |  |  | 2 |  | 2 |
| N91: Absent, scanty and rare menstruation |  |  | 2 |  | 2 |
| R11: Nausea and vomiting | 1 |  | 1 |  | 2 |
| R30: Pain associated with micturition |  |  | 2 |  | 2 |
| R49: Voice disturbances |  |  | 2 |  | 2 |
| S82: Fracture of lower leg, including ankle | 1 |  | 1 |  | 2 |
| S92: Fracture of foot, except ankle |  |  | 2 |  | 2 |
| D50: Iron deficiency anaemia | 1 |  | 2 |  | 3 |
| D64: Other anaemias |  |  | 3 |  | 3 |
| E10: Type 1 diabetes mellitus |  |  | 3 |  | 3 |
| E28: Ovarian dysfunction |  |  | 3 |  | 3 |
| F43: Reaction to severe stress, and adjustment disorders |  |  | 3 |  | 3 |
| G43: Migraine | 1 |  | 2 |  | 3 |
| H04: Disorders of lacrimal system |  |  | 3 |  | 3 |
| H57: Other disorders of eye and adnexa |  |  | 3 |  | 3 |
| I11: Hypertensive heart disease |  |  | 2 | 1 | 3 |
| I20: Angina pectoris |  |  | 2 | 1 | 3 |
| I25: Chronic ischaemic heart disease |  | 1 | 2 |  | 3 |
| J45: Asthma | 1 |  | 2 |  | 3 |
| K02: Dental caries | 1 |  | 2 |  | 3 |
| K03: Other diseases of hard tissues of teeth |  |  | 3 |  | 3 |
| K08: Other disorders of teeth and supporting structures |  |  | 3 |  | 3 |
| K12: Stomatitis and related lesions |  |  | 3 |  | 3 |
| K80: Cholelithiasis |  |  | 3 |  | 3 |
| M19: Other arthrosis |  |  | 3 |  | 3 |
| N76: Other inflammation of vagina and vulva |  |  | 3 |  | 3 |
| N84: Polyp of female genital tract |  |  | 3 |  | 3 |
| N85: Other noninflammatory disorders of uterus, except cervix |  |  | 3 |  | 3 |
| R23: Other skin changes |  |  | 2 | 1 | 3 |
| R31: Unspecified haematuria |  |  | 3 |  | 3 |
| R35: Polyuria |  |  | 3 |  | 3 |
| R59: Enlarged lymph nodes |  |  | 3 |  | 3 |
| V40-V49: Car occupant injured in transport accident |  | 1 | 2 |  | 3 |
| Y83: Surgical operation and other surgical procedures as the cause of abnormal reaction of the patient, or of later complication, without mention of misadventure at the time of the procedure |  |  | 3 |  | 3 |
| Z32: Pregnancy examination and test |  |  | 3 |  | 3 |
| B02: Zoster [herpes zoster] |  |  | 4 |  | 4 |
| H81: Disorders of vestibular function | 1 |  | 2 | 1 | 4 |
| I49: Other cardiac arrhythmias |  |  | 4 |  | 4 |
| J18: Pneumonia, organism unspecified |  |  | 4 |  | 4 |
| K05: Gingivitis and periodontal diseases |  |  | 4 |  | 4 |
| K35: Acute appendicitis |  |  | 4 |  | 4 |
| L02: Cutaneous abscess, furuncle and carbuncle |  |  | 3 | 1 | 4 |
| L70: Acne |  |  | 4 |  | 4 |
| N60: Benign mammary dysplasia | 1 |  | 3 |  | 4 |
| N95: Menopausal and other perimenopausal disorders |  |  | 4 |  | 4 |
| R60: Oedema, not elsewhere classified |  |  | 4 |  | 4 |
| S05: Injury of eye and orbit |  |  | 4 |  | 4 |
| XV: Pregnancy, childbirth and the puerperium |  |  | 4 |  | 4 |
| Z90: Acquired absence of organs, not elsewhere classified |  |  | 4 |  | 4 |
| E11: Type 2 diabetes mellitus |  |  | 5 |  | 5 |
| H10: Conjunctivitis |  | 1 | 4 |  | 5 |
| I46: Cardiac arrest |  | 1 | 4 |  | 5 |
| J30: Vasomotor and allergic rhinitis | 1 |  | 3 | 1 | 5 |
| R55: Syncope and collapse |  |  | 3 | 2 | 5 |
| R73: Elevated blood glucose level |  |  | 4 | 1 | 5 |
| U08: Personal history of COVID-19 |  |  | 5 |  | 5 |
| E03: Other hypothyroidism |  |  | 6 |  | 6 |
| E05: Thyrotoxicosis [hyperthyroidism] | 1 |  | 5 |  | 6 |
| N23: Unspecified renal colic | 1 | 1 | 4 |  | 6 |
| N93: Other abnormal uterine and vaginal bleeding |  | 2 | 4 |  | 6 |
| N94: Pain and other conditions associated with female genital organs and menstrual cycle |  | 1 | 5 |  | 6 |
| H60: Otitis externa |  | 2 | 5 |  | 7 |
| I10-I15: Hypertensive diseases |  | 1 | 6 |  | 7 |
| I21: Acute myocardial infarction |  |  | 6 | 1 | 7 |
| K21: Gastro-oesophageal reflux disease |  | 1 | 6 |  | 7 |
| R04: Haemorrhage from respiratory passages |  |  | 6 | 1 | 7 |
| R25: Abnormal involuntary movements |  | 1 | 6 |  | 7 |
| K52: Other noninfective gastroenteritis and colitis | 2 |  | 6 |  | 8 |
| Z01: Other special examinations and investigations of persons without complaint or reported diagnosis | 1 |  | 7 |  | 8 |
| F41: Other anxiety disorders |  |  | 7 | 2 | 9 |
| J32: Chronic sinusitis |  |  | 8 | 1 | 9 |
| R21: Rash and other nonspecific skin eruption | 1 |  | 8 |  | 9 |
| R43: Disturbances of smell and taste |  |  | 7 | 2 | 9 |
| Follow-up ongoing: Follow-up ongoing |  | 1 | 9 |  | 10 |
| J10: Influenza due to other identified influenza virus |  |  | 10 |  | 10 |
| M25: Other joint disorders, not elsewhere classified |  |  | 7 | 3 | 10 |
| K59: Other functional intestinal disorders |  |  | 11 |  | 11 |
| Z00: General examination and investigation of persons without complaint and reported diagnosis (rate*) | 1 (0.72) | 1 (0.73) | 9 (0.35) |  | 11 (0.35) |
| N39: Other disorders of urinary system (rate*) |  | 1 (0.73) | 10 (0.39) | 1 (0.3) | 12 (0.38) |
| R20: Disturbances of skin sensation (rate*) |  |  | 12 (0.47) | 1 (0.3) | 13 (0.41) |
| H66: Suppurative and unspecified otitis media (rate*) |  |  | 12 (0.47) | 2 (0.6) | 14 (0.44) |
| J01: Acute sinusitis (rate*) | 2 (1.45) |  | 12 (0.47) | 1 (0.3) | 15 (0.47) |
| J02: Acute pharyngitis (rate*) | 3 (2.17) | 4 (2.94) | 7 (0.27) | 1 (0.3) | 15 (0.47) |
| R50: Fever of other and unknown origin (rate*) | 3 (2.17) | 3 (2.2) | 8 (0.31) | 1 (0.3) | 15 (0.47) |
| N20: Calculus of kidney and ureter (rate*) |  | 1 (0.73) | 15 (0.58) |  | 16 (0.5) |
| T78: Adverse effects, not elsewhere classified (rate*) |  |  | 14 (0.54) | 2 (0.6) | 16 (0.5) |
| L65: Other nonscarring hair loss (rate*) | 1 (0.72) |  | 16 (0.62) |  | 17 (0.53) |
| R03: Abnormal blood-pressure reading, without diagnosis (rate*) |  | 4 (2.94) | 10 (0.39) | 3 (0.91) | 17 (0.53) |
| L29: Pruritus (rate*) |  |  | 17 (0.66) | 1 (0.3) | 18 (0.57) |
| N92: Excessive, frequent and irregular menstruation (rate*) |  | 1 (0.73) | 18 (0.7) |  | 19 (0.6) |
| R05: Cough (rate*) | 2 (1.45) | 2 (1.47) | 13 (0.5) | 2 (0.6) | 19 (0.6) |
| I95: Hypotension (rate*) |  | 1 (0.73) | 17 (0.66) | 2 (0.6) | 20 (0.63) |
| R06: Abnormalities of breathing (rate*) | 2 (1.45) | 1 (0.73) | 13 (0.5) | 4 (1.21) | 20 (0.63) |
| K29: Gastritis and duodenitis (rate*) | 2 (1.45) |  | 20 (0.78) |  | 22 (0.69) |
| L50: Urticaria (rate*) | 1 (0.72) | 3 (2.2) | 22 (0.85) |  | 26 (0.82) |
| M54: Dorsalgia (rate*) | 5 (3.62) | 2 (1.47) | 30 (1.16) | 5 (1.51) | 42 (1.32) |
| R10: Abdominal and pelvic pain (rate*) | 1 (0.72) | 3 (2.2) | 34 (1.32) | 4 (1.21) | 42 (1.32) |
| A09: Other gastroenteritis and colitis of infectious and unspecified origin (rate*) | 1 (0.72) | 2 (1.47) | 38 (1.47) | 4 (1.21) | 45 (1.41) |
| I10: Essential (primary) hypertension (rate*) | 1 (0.72) |  | 41 (1.59) | 4 (1.21) | 46 (1.45) |
| R00: Abnormalities of heart beat (rate*) | 1 (0.72) | 3 (2.2) | 43 (1.67) | 2 (0.6) | 49 (1.54) |
| R42: Dizziness and giddiness (rate*) | 8 (5.79) | 3 (2.2) | 75 (2.91) | 7 (2.12) | 93 (2.92) |
| R07: Pain in throat and chest (rate*) | 2 (1.45) | 4 (2.94) | 104 (4.03) | 6 (1.81) | 116 (3.64) |
| R51: Headache (rate*) | 11 (7.97) | 16 (11.74) | 189 (7.33) | 15 (4.54) | 231 (7.26) |
| FAG: False Grade (rate*) | 18 (13.04) | 20 (14.68) | 220 (8.53) | 12 (3.63) | 270 (8.48) |
| R52: Pain, not elsewhere classified (rate*) | 25 (18.1) | 16 (11.74) | 515 (19.98) | 14 (4.23) | 570 (17.91) |
| Z03: Medical observation and evaluation for suspected diseases and conditions (rate*) | 64 (46.35) | 84 (61.64) | 827 (32.08) | 79 (23.89) | 1054 (33.12) |
| U07: Emergency use of U07 (rate*) | 100 (72.42) | 120 (88.06) | 946 (36.7) | 102 (30.85) | 1268 (39.84) |
| FAL: False alarm (rate*) | 63 (45.62) | 72 (52.84) | 1072 (41.59) | 91 (27.52) | 1298 (40.78) |
| J00: Acute nasopharyngitis [common cold] (rate*) | 139 (100.66) | 153 (112.28) | 3369 (130.69) | 212 (64.12) | 3873 (121.69) |
| Total (rate*) | 477 (345.44) | 547 (401.41) | 8173 (317.06) | 618 (186.9) | 9815 (308.38) |
| Total Person-Time | 138084 | 136271 | 2577784 | 330651 | 3182790 |

*rate per 100000 person-day

Table 54 Final classification of all adverse events during four month active follow-up in study groups

| Final ICD-10 | Fakhravac Random | BBIBP-Corv2 Random | Fakhravac Non_Random | BBIBP-Corv2 Non_Random | Total |
| --- | --- | --- | --- | --- | --- |
| A04.7: Enterocolitis due to Clostridium difficile |  |  | 1 |  | 1 |
| A05.0: Foodborne staphylococcal intoxication |  |  | 1 |  | 1 |
| B00: Herpesviral [herpes simplex] infections |  |  | 1 |  | 1 |
| B00.2: Herpesviral gingivostomatitis and pharyngotonsillitis |  | 1 |  |  | 1 |
| B07: Viral warts |  |  | 1 |  | 1 |
| B37.3: Candidiasis of vulva and vagina |  |  | 1 |  | 1 |
| B98.0: Helicobacter pylori [H.pylori] as the cause of diseases classified to other chapters |  |  | 1 |  | 1 |
| D34: Benign neoplasm of thyroid gland |  |  | 1 |  | 1 |
| D35.2: Benign neoplasm: Pituitary gland | 1 |  |  |  | 1 |
| D64.9: Anaemia, unspecified |  |  | 1 |  | 1 |
| D72.8: Other specified disorders of white blood cells |  |  | 1 |  | 1 |
| E05.0: Thyrotoxicosis with diffuse goitre |  |  | 1 |  | 1 |
| E10-E14: Diabetes mellitus |  |  | 1 |  | 1 |
| E14: Unspecified diabetes mellitus |  |  | 1 |  | 1 |
| E23.7: Disorder of pituitary gland, unspecified |  |  | 1 |  | 1 |
| E28.3: Primary ovarian failure |  |  | 1 |  | 1 |
| E34.9: Endocrine disorder, unspecified |  |  |  | 1 | 1 |
| E66: Obesity |  |  | 1 |  | 1 |
| E78.5: Hyperlipidaemia, unspecified |  |  | 1 |  | 1 |
| F39: Unspecified mood [affective] disorder |  |  |  | 1 | 1 |
| F41: Other anxiety disorders |  |  | 1 |  | 1 |
| F45.9: Somatoform disorder, unspecified |  |  | 1 |  | 1 |
| F52.2: Failure of genital response |  |  | 1 |  | 1 |
| F60.6: Anxious [avoidant] personality disorder |  |  | 1 |  | 1 |
| F99: Mental disorder, not otherwise specified |  |  |  | 1 | 1 |
| G00: Bacterial meningitis, not elsewhere classified |  |  | 1 |  | 1 |
| G25.8: Other specified extrapyramidal and movement disorders |  |  | 1 |  | 1 |
| G40.9: Epilepsy, unspecified |  |  |  | 1 | 1 |
| G45.9: Transient cerebral ischaemic attack, unspecified |  |  | 1 |  | 1 |
| G51.0: Bell palsy |  |  | 1 |  | 1 |
| H04: Disorders of lacrimal system |  |  | 1 |  | 1 |
| H34.8: Other retinal vascular occlusions |  |  | 1 |  | 1 |
| H52.1: Myopia |  |  | 1 |  | 1 |
| H53.8: Other visual disturbances |  |  | 1 |  | 1 |
| H54.6: Moderate visual impairment, monocular |  |  | 1 |  | 1 |
| H54.9: Unspecified visual impairment (binocular) |  |  | 1 |  | 1 |
| H60.3: Other infective otitis externa |  | 1 |  |  | 1 |
| H62.2*: Otitis externa in mycoses |  |  | 1 |  | 1 |
| H81.0: Ménière disease |  |  |  | 1 | 1 |
| H91.2: Sudden idiopathic hearing loss |  |  | 1 |  | 1 |
| H92: Otalgia and effusion of ear |  | 1 |  |  | 1 |
| H92.0: Otalgia |  |  | 1 |  | 1 |
| H93.1: Tinnitus |  |  | 1 |  | 1 |
| I11.0: Hypertensive heart disease with (congestive) heart failure |  |  | 1 |  | 1 |
| I15.9: Secondary hypertension, unspecified |  |  | 1 |  | 1 |
| I20: Angina pectoris |  |  | 1 |  | 1 |
| I20.0: Unstable angina |  |  | 1 |  | 1 |
| I20.8: Other forms of angina pectoris |  |  |  | 1 | 1 |
| I25.9: Chronic ischaemic heart disease, unspecified |  |  | 1 |  | 1 |
| I26: Pulmonary embolism |  | 1 |  |  | 1 |
| I46.1: Sudden cardiac death, so described |  |  | 1 |  | 1 |
| I49: Other cardiac arrhythmias |  |  | 1 |  | 1 |
| I50: Heart failure |  |  | 1 |  | 1 |
| I50.0: Congestive heart failure |  |  | 1 |  | 1 |
| I61: Intracerebral haemorrhage |  | 1 |  |  | 1 |
| I86.1: Scrotal varices |  |  | 1 |  | 1 |
| I88: Nonspecific lymphadenitis |  |  |  | 1 | 1 |
| J02.9: Acute pharyngitis, unspecified |  |  |  | 1 | 1 |
| J03.8: Acute tonsillitis due to other specified organisms |  |  | 1 |  | 1 |
| J03.9: Acute tonsillitis, unspecified |  |  | 1 |  | 1 |
| J09: Influenza due to certain identified influenza virus |  |  | 1 |  | 1 |
| J15.9: Bacterial pneumonia, unspecified |  |  | 1 |  | 1 |
| J30.2: Other seasonal allergic rhinitis |  |  | 1 |  | 1 |
| J30.4: Allergic rhinitis, unspecified |  |  | 1 |  | 1 |
| J34.1: Cyst and mucocele of nose and nasal sinus |  |  | 1 |  | 1 |
| J40: Bronchitis, not specified as acute or chronic |  |  | 1 |  | 1 |
| J45: Asthma |  |  | 1 |  | 1 |
| K00: Disorders of tooth development and eruption | 1 |  |  |  | 1 |
| K02.9: Dental caries, unspecified | 1 |  |  |  | 1 |
| K04.7: Periapical abscess without sinus |  |  | 1 |  | 1 |
| K05: Gingivitis and periodontal diseases |  |  | 1 |  | 1 |
| K05.0: Acute gingivitis |  |  | 1 |  | 1 |
| K06: Other disorders of gingiva and edentulous alveolar ridge |  |  | 1 |  | 1 |
| K11.7: Disturbances of salivary secretion |  | 1 |  |  | 1 |
| K29.7: Gastritis, unspecified |  |  | 1 |  | 1 |
| K31: Other diseases of stomach and duodenum |  |  | 1 |  | 1 |
| K40: Inguinal hernia |  |  | 1 |  | 1 |
| K42: Umbilical hernia |  |  | 1 |  | 1 |
| K50: Crohn disease [regional enteritis] |  |  | 1 |  | 1 |
| K50-K52: Noninfective enteritis and colitis |  |  |  | 1 | 1 |
| K58: Irritable bowel syndrome |  |  | 1 |  | 1 |
| K58.8: Other and unspecified irritable bowel syndrome |  |  | 1 |  | 1 |
| K60.2: Anal fissure, unspecified |  |  | 1 |  | 1 |
| K61.0: Anal abscess |  |  | 1 |  | 1 |
| K70.0: Alcoholic fatty liver |  |  | 1 |  | 1 |
| K74: Fibrosis and cirrhosis of liver |  |  |  | 1 | 1 |
| K76.0: Fatty (change of) liver, not elsewhere classified |  |  | 1 |  | 1 |
| K80.5: Calculus of bile duct without cholangitis or cholecystitis |  |  | 1 |  | 1 |
| K81: Cholecystitis |  |  | 1 |  | 1 |
| K81.0: Acute cholecystitis |  |  | 1 |  | 1 |
| K92.1: Melaena |  |  |  | 1 | 1 |
| K92.2: Gastrointestinal haemorrhage, unspecified |  |  | 1 |  | 1 |
| L02: Cutaneous abscess, furuncle and carbuncle |  |  | 1 |  | 1 |
| L02.9: Cutaneous abscess, furuncle and carbuncle, unspecified |  |  | 1 |  | 1 |
| L03.3: Cellulitis of trunk |  |  | 1 |  | 1 |
| L05: Pilonidal cyst |  |  | 1 |  | 1 |
| L23: Allergic contact dermatitis |  |  | 1 |  | 1 |
| L30.9: Dermatitis, unspecified |  |  | 1 |  | 1 |
| L40: Psoriasis | 1 |  |  |  | 1 |
| L42: Pityriasis rosea |  |  | 1 |  | 1 |
| L64: Androgenic alopecia |  |  |  | 1 | 1 |
| M13.1: Monoarthritis, not elsewhere classified |  | 1 |  |  | 1 |
| M17: Gonarthrosis [arthrosis of knee] |  |  | 1 |  | 1 |
| M46.4: Discitis, unspecified |  |  |  | 1 | 1 |
| M51: Other intervertebral disc disorders | 1 |  |  |  | 1 |
| M51.1†: Lumbar and other intervertebral disc disorders with radiculopathy |  |  | 1 |  | 1 |
| M79.1: Myalgia |  |  | 1 |  | 1 |
| M79.6: Pain in limb |  |  | 1 |  | 1 |
| N13.3: Other and unspecified hydronephrosis |  |  | 1 |  | 1 |
| N30: Cystitis | 1 |  |  |  | 1 |
| N30.0: Acute cystitis |  |  | 1 |  | 1 |
| N39.3: Stress incontinence |  |  | 1 |  | 1 |
| N40: Hyperplasia of prostate |  |  | 1 |  | 1 |
| N41: Inflammatory diseases of prostate |  |  | 1 |  | 1 |
| N41.1: Chronic prostatitis |  |  | 1 |  | 1 |
| N48.8: Other specified disorders of penis |  |  | 1 |  | 1 |
| N50.1: Vascular disorders of male genital organs |  |  | 1 |  | 1 |
| N60.8: Other benign mammary dysplasias | 1 |  |  |  | 1 |
| N61: Inflammatory disorders of breast |  |  | 1 |  | 1 |
| N63: Unspecified lump in breast |  |  | 1 |  | 1 |
| N64.4: Mastodynia |  |  | 1 |  | 1 |
| N64.5: Other signs and symptoms in breast |  |  | 1 |  | 1 |
| N80: Endometriosis |  |  | 1 |  | 1 |
| N83.0: Follicular cyst of ovary |  |  | 1 |  | 1 |
| N84.1: Polyp of cervix uteri |  |  | 1 |  | 1 |
| N91: Absent, scanty and rare menstruation |  |  | 1 |  | 1 |
| N91.2: Amenorrhoea, unspecified |  |  | 1 |  | 1 |
| N94: Pain and other conditions associated with female genital organs and menstrual cycle |  |  | 1 |  | 1 |
| N95.0: Postmenopausal bleeding |  |  | 1 |  | 1 |
| O00-O99: Pregnancy, childbirth and the puerperium |  |  | 1 |  | 1 |
| R03.1: Nonspecific low blood-pressure reading |  |  | 1 |  | 1 |
| R06: Abnormalities of breathing |  |  |  | 1 | 1 |
| R07.0: Pain in throat |  |  | 1 |  | 1 |
| R10.2: Pelvic and perineal pain |  |  | 1 |  | 1 |
| R12: Heartburn |  |  | 1 |  | 1 |
| R14: Flatulence and related conditions |  |  | 1 |  | 1 |
| R18: Ascites |  |  |  | 1 | 1 |
| R22.9: Localized swelling, mass and lump, unspecified |  | 1 |  |  | 1 |
| R23.3: Spontaneous ecchymoses |  |  | 1 |  | 1 |
| R30.0: Dysuria |  |  | 1 |  | 1 |
| R30.9: Painful micturition, unspecified |  |  | 1 |  | 1 |
| R41: Other symptoms and signs involving cognitive functions and awareness |  | 1 |  |  | 1 |
| R43: Disturbances of smell and taste |  |  | 1 |  | 1 |
| R50.8: Other specified fever |  |  | 1 |  | 1 |
| R59.0: Localized enlarged lymph nodes |  |  | 1 |  | 1 |
| R59.1: Generalized enlarged lymph nodes |  |  | 1 |  | 1 |
| R59.9: Enlarged lymph nodes, unspecified |  |  | 1 |  | 1 |
| R60: Oedema, not elsewhere classified |  |  | 1 |  | 1 |
| R60.9: Oedema, unspecified |  |  | 1 |  | 1 |
| R96: Other sudden death, cause unknown |  |  | 1 |  | 1 |
| S02.2: Fracture of nasal bones |  |  | 1 |  | 1 |
| S27.9: Injury of unspecified intrathoracic organ |  |  |  | 1 | 1 |
| S36.3: Injury of stomach |  |  | 1 |  | 1 |
| S52: Fracture of forearm |  | 1 |  |  | 1 |
| S60: Superficial injury of wrist and hand |  |  | 1 |  | 1 |
| S76.2: Injury of adductor muscle and tendon of thigh |  |  | 1 |  | 1 |
| S82.4: Fracture of fibula alone |  |  | 1 |  | 1 |
| S82.5: Fracture of medial malleolus | 1 |  |  |  | 1 |
| S92.3: Fracture of metatarsal bone |  |  | 1 |  | 1 |
| S92.9: Fracture of foot, unspecified |  |  | 1 |  | 1 |
| S93.2: Rupture of ligaments at ankle and foot level |  |  |  | 1 | 1 |
| T14.9: Injury, unspecified |  |  | 1 |  | 1 |
| T23: Burn and corrosion of wrist and hand |  |  | 1 |  | 1 |
| T78.2: Anaphylactic shock, unspecified |  |  | 1 |  | 1 |
| T88.7: Unspecified adverse effect of drug or medicament |  |  | 1 |  | 1 |
| V03: Pedestrian injured in collision with car, pick-up truck or van |  |  | 1 |  | 1 |
| V29.3: Motorcycle rider [any] injured in unspecified nontraffic accident |  |  | 1 |  | 1 |
| X47.4: Accidental poisoning by and exposure to carbon monoxide from unspecified sources |  |  | 1 |  | 1 |
| Y58.4: Tetanus vaccine |  |  | 1 |  | 1 |
| Y83.2: Surgical operation with anastomosis, bypass or graft |  |  | 1 |  | 1 |
| Y83.6: Removal of other organ (partial) (total) |  |  | 1 |  | 1 |
| Y83.9: Surgical procedure, unspecified |  |  | 1 |  | 1 |
| Z10: Routine general health check-up of defined subpopulation |  |  | 1 |  | 1 |
| Z30.2: Sterilization |  |  | 1 |  | 1 |
| Z33: Pregnant state, incidental |  |  | 1 |  | 1 |
| Z34: Supervision of normal pregnancy |  |  | 1 |  | 1 |
| Z41.1: Other plastic surgery for unacceptable cosmetic appearance |  | 1 |  |  | 1 |
| Z47: Other orthopaedic follow-up care |  | 1 |  |  | 1 |
| Z51.2: Other chemotherapy |  |  | 1 |  | 1 |
| Z86.5: Personal history of other mental and behavioural disorders |  |  |  | 1 | 1 |
| Z97.5: Presence of (intrauterine) contraceptive device |  |  | 1 |  | 1 |
| A08.4: Viral intestinal infection, unspecified |  |  | 2 |  | 2 |
| D64: Other anaemias |  |  | 2 |  | 2 |
| E00-E07: Disorders of thyroid gland |  |  |  | 2 | 2 |
| E03.9: Hypothyroidism, unspecified |  |  | 2 |  | 2 |
| E16.2: Hypoglycaemia, unspecified |  |  | 2 |  | 2 |
| E28.2: Polycystic ovarian syndrome |  |  | 2 |  | 2 |
| F32: Depressive episode |  |  | 2 |  | 2 |
| H04.1: Other disorders of lacrimal gland |  |  | 2 |  | 2 |
| H11.9: Disorder of conjunctiva, unspecified |  | 2 |  |  | 2 |
| H26.9: Cataract, unspecified |  |  | 2 |  | 2 |
| H40: Glaucoma |  |  | 2 |  | 2 |
| H60.9: Otitis externa, unspecified |  |  | 2 |  | 2 |
| H61.2: Impacted cerumen |  |  | 2 |  | 2 |
| H67: Otitis media in diseases classified elsewhere |  |  | 1 | 1 | 2 |
| I11: Hypertensive heart disease |  |  | 1 | 1 | 2 |
| I25: Chronic ischaemic heart disease |  | 1 | 1 |  | 2 |
| I95.2: Hypotension due to drugs |  | 1 | 1 |  | 2 |
| J10.1: Influenza with other respiratory manifestations, other influenza virus identified |  |  | 2 |  | 2 |
| J11: Influenza, virus not identified | 1 |  | 1 |  | 2 |
| J32.1: Chronic frontal sinusitis |  |  | 2 |  | 2 |
| J45.0: Predominantly allergic asthma | 1 |  | 1 |  | 2 |
| K02: Dental caries |  |  | 2 |  | 2 |
| K05.2: Acute periodontitis |  |  | 2 |  | 2 |
| K21.9: Gastro-oesophageal reflux disease without oesophagitis |  |  | 2 |  | 2 |
| K25: Gastric ulcer |  |  | 2 |  | 2 |
| K52: Other noninfective gastroenteritis and colitis |  |  | 2 |  | 2 |
| K64: Haemorrhoids and perianal venous thrombosis |  |  | 2 |  | 2 |
| K80.2: Calculus of gallbladder without cholecystitis |  |  | 2 |  | 2 |
| L02.4: Cutaneous abscess, furuncle and carbuncle of limb |  |  | 1 | 1 | 2 |
| L04: Acute lymphadenitis |  |  | 2 |  | 2 |
| L29.9: Pruritus, unspecified |  |  | 2 |  | 2 |
| L50.0: Allergic urticaria |  |  | 2 |  | 2 |
| L93: Lupus erythematosus |  |  | 1 | 1 | 2 |
| L98.9: Disorder of skin and subcutaneous tissue, unspecified |  |  |  | 2 | 2 |
| M10: Gout |  |  | 2 |  | 2 |
| M95.0: Acquired deformity of nose |  |  | 1 | 1 | 2 |
| N84: Polyp of female genital tract |  |  | 2 |  | 2 |
| N92.6: Irregular menstruation, unspecified |  |  | 2 |  | 2 |
| R11: Nausea and vomiting | 1 |  | 1 |  | 2 |
| R23.2: Flushing |  |  | 1 | 1 | 2 |
| R49.0: Dysphonia |  |  | 2 |  | 2 |
| R60.0: Localized oedema |  |  | 2 |  | 2 |
| Z00.4: General psychiatric examination, not elsewhere classified | 1 |  | 1 |  | 2 |
| D50: Iron deficiency anaemia | 1 |  | 2 |  | 3 |
| E10: Type 1 diabetes mellitus |  |  | 3 |  | 3 |
| F41.9: Anxiety disorder, unspecified |  |  | 3 |  | 3 |
| F43.0: Acute stress reaction |  |  | 3 |  | 3 |
| G43: Migraine | 1 |  | 2 |  | 3 |
| H57.1: Ocular pain |  |  | 3 |  | 3 |
| H81.1: Benign paroxysmal vertigo | 1 |  | 2 |  | 3 |
| I49.9: Cardiac arrhythmia, unspecified |  |  | 3 |  | 3 |
| J30: Vasomotor and allergic rhinitis | 1 |  | 1 | 1 | 3 |
| K03: Other diseases of hard tissues of teeth |  |  | 3 |  | 3 |
| K08.8: Other specified disorders of teeth and supporting structures |  |  | 3 |  | 3 |
| K12.0: Recurrent oral aphthae |  |  | 3 |  | 3 |
| L50.9: Urticaria, unspecified | 1 | 2 |  |  | 3 |
| M19.9: Arthrosis, unspecified |  |  | 3 |  | 3 |
| N60.0: Solitary cyst of breast |  |  | 3 |  | 3 |
| N76.0: Acute vaginitis |  |  | 3 |  | 3 |
| N85.0: Endometrial glandular hyperplasia |  |  | 3 |  | 3 |
| N92.0: Excessive and frequent menstruation with regular cycle |  |  | 3 |  | 3 |
| N95: Menopausal and other perimenopausal disorders |  |  | 3 |  | 3 |
| R07.1: Chest pain on breathing |  |  | 3 |  | 3 |
| R20.8: Other and unspecified disturbances of skin sensation |  |  | 2 | 1 | 3 |
| R31: Unspecified haematuria |  |  | 3 |  | 3 |
| R35: Polyuria |  |  | 3 |  | 3 |
| V40-V49: Car occupant injured in transport accident |  | 1 | 2 |  | 3 |
| Z01.2: Dental examination |  |  | 3 |  | 3 |
| Z32.1: Pregnancy confirmed |  |  | 3 |  | 3 |
| B02: Zoster [herpes zoster] |  |  | 4 |  | 4 |
| E03: Other hypothyroidism |  |  | 4 |  | 4 |
| H60: Otitis externa |  | 1 | 3 |  | 4 |
| H66.9: Otitis media, unspecified |  |  | 4 |  | 4 |
| I46: Cardiac arrest |  | 1 | 3 |  | 4 |
| J10: Influenza due to other identified influenza virus |  |  | 4 |  | 4 |
| J10.8: Influenza with other manifestations, other influenza virus identified |  |  | 4 |  | 4 |
| J18: Pneumonia, organism unspecified |  |  | 4 |  | 4 |
| K29.9: Gastroduodenitis, unspecified |  |  | 4 |  | 4 |
| K35: Acute appendicitis |  |  | 4 |  | 4 |
| L70: Acne |  |  | 4 |  | 4 |
| R03.0: Elevated blood-pressure reading, without diagnosis of hypertension |  | 2 | 2 |  | 4 |
| R06.7: Sneezing | 1 | 1 | 1 | 1 | 4 |
| S05: Injury of eye and orbit |  |  | 4 |  | 4 |
| XV: Pregnancy, childbirth and the puerperium |  |  | 4 |  | 4 |
| Z90.7: Acquired absence of genital organ(s) |  |  | 4 |  | 4 |
| E05: Thyrotoxicosis [hyperthyroidism] | 1 |  | 4 |  | 5 |
| E11: Type 2 diabetes mellitus |  |  | 5 |  | 5 |
| F41.0: Panic disorder [episodic paroxysmal anxiety] |  |  | 3 | 2 | 5 |
| H10: Conjunctivitis |  | 1 | 4 |  | 5 |
| K21: Gastro-oesophageal reflux disease |  | 1 | 4 |  | 5 |
| K59.0: Constipation |  |  | 5 |  | 5 |
| L65.9: Nonscarring hair loss, unspecified | 1 |  | 4 |  | 5 |
| N94.6: Dysmenorrhoea, unspecified |  | 1 | 4 |  | 5 |
| R00.0: Tachycardia, unspecified |  |  | 5 |  | 5 |
| R07.3: Other chest pain | 1 |  | 4 |  | 5 |
| R55: Syncope and collapse |  |  | 3 | 2 | 5 |
| R73.9: Hyperglycaemia, unspecified |  |  | 4 | 1 | 5 |
| U08: Personal history of COVID-19 |  |  | 5 |  | 5 |
| Z01: Other special examinations and investigations of persons without complaint or reported diagnosis | 1 |  | 4 |  | 5 |
| K52.9: Noninfective gastroenteritis and colitis, unspecified | 2 |  | 4 |  | 6 |
| K59.1: Functional diarrhoea |  |  | 6 |  | 6 |
| L65: Other nonscarring hair loss |  |  | 6 |  | 6 |
| L65.8: Other specified nonscarring hair loss |  |  | 6 |  | 6 |
| M54.2: Cervicalgia | 1 |  | 5 |  | 6 |
| N23: Unspecified renal colic | 1 | 1 | 4 |  | 6 |
| N92.1: Excessive and frequent menstruation with irregular cycle |  | 1 | 5 |  | 6 |
| N93: Other abnormal uterine and vaginal bleeding |  | 2 | 4 |  | 6 |
| I10-I15: Hypertensive diseases |  | 1 | 6 |  | 7 |
| I21: Acute myocardial infarction |  |  | 6 | 1 | 7 |
| J32: Chronic sinusitis |  |  | 6 | 1 | 7 |
| R04.0: Epistaxis |  |  | 6 | 1 | 7 |
| R25.2: Cramp and spasm |  | 1 | 6 |  | 7 |
| N92: Excessive, frequent and irregular menstruation |  |  | 8 |  | 8 |
| R07: Pain in throat and chest |  | 1 | 7 |  | 8 |
| R43.0: Anosmia |  |  | 6 | 2 | 8 |
| R21: Rash and other nonspecific skin eruption | 1 |  | 8 |  | 9 |
| Z00.0: General medical examination |  | 1 | 8 |  | 9 |
| Follow-up ongoing: Follow-up ongoing |  | 1 | 9 |  | 10 |
| H66: Suppurative and unspecified otitis media |  |  | 8 | 2 | 10 |
| M25.5: Pain in joint |  |  | 7 | 3 | 10 |
| R10.4: Other and unspecified abdominal pain (rate*) | 1 (0.72) |  | 7 (0.27) | 2 (0.6) | 10 (0.31) |
| R20.2: Paraesthesia of skin (rate*) |  |  | 10 (0.39) |  | 10 (0.31) |
| N39.0: Urinary tract infection, site not specified (rate*) |  | 1 (0.73) | 9 (0.35) | 1 (0.3) | 11 (0.35) |
| A09.9: Gastroenteritis and colitis of unspecified origin (rate*) |  |  | 12 (0.47) |  | 12 (0.38) |
| R03: Abnormal blood-pressure reading, without diagnosis (rate*) |  | 2 (1.47) | 7 (0.27) | 3 (0.91) | 12 (0.38) |
| A09: Other gastroenteritis and colitis of infectious and unspecified origin (rate*) | 1 (0.72) | 1 (0.73) | 11 (0.43) | 1 (0.3) | 14 (0.44) |
| J02: Acute pharyngitis (rate*) | 3 (2.17) | 4 (2.94) | 7 (0.27) |  | 14 (0.44) |
| R50: Fever of other and unknown origin (rate*) | 3 (2.17) | 3 (2.2) | 7 (0.27) | 1 (0.3) | 14 (0.44) |
| J01: Acute sinusitis (rate*) | 2 (1.45) |  | 12 (0.47) | 1 (0.3) | 15 (0.47) |
| R06.0: Dyspnoea (rate*) | 1 (0.72) |  | 12 (0.47) | 2 (0.6) | 15 (0.47) |
| R10: Abdominal and pelvic pain (rate*) |  |  | 15 (0.58) |  | 15 (0.47) |
| T78.4: Allergy, unspecified (rate*) |  |  | 13 (0.5) | 2 (0.6) | 15 (0.47) |
| L29: Pruritus (rate*) |  |  | 15 (0.58) | 1 (0.3) | 16 (0.5) |
| N20.0: Calculus of kidney (rate*) |  | 1 (0.73) | 15 (0.58) |  | 16 (0.5) |
| R10.1: Pain localized to upper abdomen (rate*) |  | 3 (2.2) | 11 (0.43) | 2 (0.6) | 16 (0.5) |
| K29: Gastritis and duodenitis (rate*) | 2 (1.45) |  | 15 (0.58) |  | 17 (0.53) |
| I95: Hypotension (rate*) |  |  | 16 (0.62) | 2 (0.6) | 18 (0.57) |
| A09.0: Other and unspecified gastroenteritis and colitis of infectious origin (rate*) |  | 1 (0.73) | 15 (0.58) | 3 (0.91) | 19 (0.6) |
| R05: Cough (rate*) | 2 (1.45) | 2 (1.47) | 13 (0.5) | 2 (0.6) | 19 (0.6) |
| L50: Urticaria (rate*) |  | 1 (0.73) | 20 (0.78) |  | 21 (0.66) |
| M54.5: Low back pain (rate*) | 4 (2.9) | 2 (1.47) | 25 (0.97) | 5 (1.51) | 36 (1.13) |
| R00.2: Palpitations (rate*) | 1 (0.72) | 3 (2.2) | 38 (1.47) | 2 (0.6) | 44 (1.38) |
| I10: Essential (primary) hypertension (rate*) | 1 (0.72) |  | 41 (1.59) | 4 (1.21) | 46 (1.45) |
| R42: Dizziness and giddiness (rate*) | 8 (5.79) | 3 (2.2) | 75 (2.91) | 7 (2.12) | 93 (2.92) |
| R07.4: Chest pain, unspecified (rate*) | 1 (0.72) | 3 (2.2) | 89 (3.45) | 6 (1.81) | 99 (3.11) |
| U07.2: Emergency use of U07.2 (rate*) | 11 (7.97) | 7 (5.14) | 144 (5.59) | 22 (6.65) | 184 (5.78) |
| R51: Headache (rate*) | 11 (7.97) | 16 (11.74) | 189 (7.33) | 15 (4.54) | 231 (7.26) |
| FAG: False Grade (rate*) | 18 (13.04) | 20 (14.68) | 220 (8.53) | 12 (3.63) | 270 (8.48) |
| R52: Pain, not elsewhere classified (rate*) | 25 (18.1) | 16 (11.74) | 515 (19.98) | 14 (4.23) | 570 (17.91) |
| Z03.8: Observation for other suspected diseases and conditions (rate*) | 64 (46.35) | 84 (61.64) | 827 (32.08) | 79 (23.89) | 1054 (33.12) |
| U07.1: Emergency use of U07.1 (rate*) | 89 (64.45) | 113 (82.92) | 802 (31.11) | 80 (24.19) | 1084 (34.06) |
| FAL: False alarm (rate*) | 63 (45.62) | 72 (52.84) | 1072 (41.59) | 91 (27.52) | 1298 (40.78) |
| J00: Acute nasopharyngitis [common cold] (rate*) | 139 (100.66) | 153 (112.28) | 3369 (130.69) | 212 (64.12) | 3873 (121.69) |
| Total (rate*) | 477 (345.44) | 547 (401.41) | 8173 (317.06) | 618 (186.9) | 9815 (308.38) |
| Total Person-Time | 138084 | 136271 | 2577784 | 330651 | 3182790 |

*rate per 100000 person-day

### Withdrawal from the study after receiving the vaccine

Table 55 Reasons for withdrawal from the study by study arm

|  | Fakhravac Random | | | BBIBP-Corv2 Random | | | Fakhravac Non-Random | | | BBIBP-Corv2 Non-Random | | | Total |
| --- | --- | --- | --- | --- | --- | --- | --- | --- | --- | --- | --- | --- | --- |
| Reason for leaving the study | After first vaccine | After second vaccine | 14 days after second vaccine | After first vaccine | After second vaccine | 14 days after second vaccine | After first vaccine | After second vaccine | 14 days after second vaccine | After first vaccine | After second vaccine | 14 days after second vaccine |  |
| Serious reaction to previous vaccine dose |  |  |  |  |  |  | 1 |  |  |  |  |  | 1 |
| Pregnancy |  |  |  |  |  |  | 10 | 2 | 5 | 2 |  | 1 | 20 |
| Overt biochemical and hematologic changes in the subject that threatens his/her health judged by the principle investigator |  |  |  |  |  |  | 1 |  |  |  |  |  | 1 |
| Receiving another vaccine | 4 |  | 18 | 1 |  | 28 | 12 | 2 | 76 | 6 | 2 | 73 | 222 |
| Withdrawing consent/not willing to continue in the study | 10 |  |  | 6 |  | 1 | 126 | 7 | 9 | 35 |  | 2 | 196 |
| Other reasons for protocol deviation (did not responded to follow up calls) | 23 |  |  | 31 | 1 |  | 1795 | 24 | 10 | 559 | 2 | 5 | 2450 |
| Death |  |  |  | 1 |  | 1 | 2 | 1 | 4 |  |  |  | 9 |
| Covid-19 Diagnosed (PCR pos/neg) | 16 | 19 | 65 | 11 | 19 | 85 | 85 | 44 | 789 | 16 | 3 | 83 | 1235 |
| Total | 53 | 19 | 83 | 50 | 20 | 115 | 2032 | 80 | 893 | 618 | 7 | 164 | 4134 |
